# On uniform stability and numerical simulations of complex valued neural networks involving generalized Caputo fractional order

**DOI:** 10.1038/s41598-024-53670-4

**Published:** 2024-02-19

**Authors:** Sumati Kumari Panda, Thabet Abdeljawad, A. M. Nagy

**Affiliations:** 1 Department of Mathematics, GMR Institute of Technology, Rajam, Andhra Pradesh 532127 India; 2 Department of Mathematics and Sciences, Prince Sultan University, Riyadh, 11586 Saudi Arabia; 3 Department of Medical Research, China Medical University, Taichung, 40402 Taiwan; 4 Department of Mathematics and Applied Mathematics, Sefako Makgatho Health Sciences University, Garankuwa, Medusa, 0204 South Africa; 5 Department of Mathematics, College of Science, Kuwait University, 13060 Safat, Kuwait; 6 Department of Mathematics, Faculty of Science, Benha University, Benha, 13518 Egypt

**Keywords:** Applied mathematics, Pure mathematics

## Abstract

The dynamics and existence results of generalized Caputo fractional derivatives have been studied by several authors. Uniform stability and equilibrium in fractional-order neural networks with generalized Caputo derivatives in real-valued settings, however, have not been extensively studied. In contrast to earlier studies, we first investigate the uniform stability and equilibrium results for complex-valued neural networks within the framework of a generalized Caputo fractional derivative. We investigate the intermittent behavior of complex-valued neural networks in generalized Caputo fractional-order contexts. Numerical results are supplied to demonstrate the viability and accuracy of the presented results. At the end of the article, a few open questions are posed.

## Introduction

The history of fractional calculus is actually quite similar to that of classical calculus, hence it is not a novel subject^1^. The notions of fractional calculus have developed significantly-even frantically-since the advent of the fractional derivative, with the majority of the contributions coming from pure mathematicians rather than applied mathematicians. Consequently, there has been a significant rise in the variety of fractional integral operators over the past few decades (check^2–4^ for more details). The contributions of the fractional order derivative is common in both science and engineering fields. Take a look at this, for instance:

- The authors of^5^ have expanded the glucose molecule’s graph representation and taken into consideration contemporary modeling of the fractional derivatives on each graph edge. By taking multiple constraints on the existing operators, the authors have provided two different insights in the context of integral boundary value requirements.
- In light of fractional derivatives, the authors of^6^ examined a wide range of physical structures, including thermal transmission, controllers with PID tuning, and network fabrication via stochastic algorithms.

Caputo fractional-order systems and/or generalized Caputo fractional-order systems are often used in modeling and computation^7–11^.

A particular class of computations stimulated by data platforms referred to as artificial neural networks (simply, we called, ANNs) aims to imitate the functions and operations of the human brain. With the advancement of artificial intelligence (AI), in particular deep learning, ANN-based machine intelligence algorithms have substantial usage in various fields that permeate our everyday lives. Digital devices can now make realistic-sounding speech and pitches for music for practical purposes by using tools like automated facial recognition. They can also divide the speech of several speakers into separate waves of sound for each individual speaker using this tool, as shown in^12^. Complex numbers are frequently used in a wide range of practical uses, including recognition of speech, processing of pictures, automation, and communication systems. This demonstrates the possibilities for utilizing numbers which are complex-valued to personify parameters like weights in various applications for artificial neural networks. Adopting a complex-valued system compared to a real-valued system makes more sense in light of the crucial nature of amplitude and dimensions in learning theory^13^. CVNNs that process data utilizing multiple variables and parameters^14^. Evidently, there are facts to suggest that CVNNs can be helpful in sectors where the data that is being encountered is depicted in a complicated way, either by design or by nature. Real-valued and/or convolutional neural networks have been the subject of extensive studies since the mid-1980s. However, compared to neural networks that are real-valued, research on CVNNs has been comparatively under-reported during the rise of deep learning. In CVNN analysis, deep neural networks and particular signal-processing challenges like circuit normalization have gotten the most interest. The reason for this is that a complex-valued activating term is unable to be both complex-differentiable and bounded. The criteria that a complex-valued activation be jointly bounded and complex-differentiable might have failed to be gratified, and stimulation that is distinct with regard to both imaginary and real elements may instead be recommended in their position, according to^15^. The development of this field of study is still in its earliest phases.

Fractional-order neural networks, or FONN for short, have gained prominence in recent years in both scientific and computational research. FONN yields extremely accurate results and noteworthy improvements by combining neural networks with fractional derivatives. Fractional-order neural network models have drawn more attention in several fields and are now a significant research area. FONN is well-known for being a practical modeling, testing, and educational tool for systems with dynamics in a variety of disciplines, including neural technology, nanophysics, and engineering. New advancements in the field of FONN are driven by challenges and revelations from scientific and technical research. The FONN framework outperforms the traditional integer-order neural network framework for evaluating the memory and inheritance components of various protocols that are in neural systems.

We are aware that the memory and accumulated characteristics of numerous things and activities can be effectively described by fractional-order derivatives. It’s also important to remember that algorithms with fractional-orders have limitless memory. However, it has also been demonstrated by actual neuron investigations. On the basis of such attributes, synthetic links to neural networks have been created using nonlinear analysis. The authors of^16^ recently established the conditions needed for uniform stability as well as the existence of the uniqueness. Regarding the testing of models, it additionally becomes vital to take into account the possible importance of neural systems in the setting of fractional order. Consequently, though it may be a fractional derivative or integral, it is a significant advancement to incorporate a memory concept into neural networks. Therefore, research on fractional-order neural networks is important. Due to the extensive use of complex signals in neural network functions, such as those in^17–19^ and the references therein, understanding CVNNs is crucial for developing useful tools. However, there are few studies on generalized Caputo fractional-order neural networks in a real field (see, for example^20–24^).

On the other hand, *metric spaces* have recently undergone substantial generalizations, and relevant fixed-point theorems have utilized by many scientific domains, such as mathematical modeling domains, applied physics, control theory, and nonlinear analysis (see, for example^25–40^).

Motivated by the above studies, we examine how complex-valued neural networks behave intermittently under generalized Caputo fractional-order contexts. When working with fractional-order structures, neural network methods are distinct from those used for traditional integer-order settings. In this article, we first investigate the uniform stability and equilibrium results for complex-valued neural networks within the framework of a generalized Caputo fractional derivative. Moreover, it is worth noticing that the contribution of the fractional order derivative is that, with the help of the fractional derivative order, the neural network can determine its activation function.

The complex-valued metric space was originally developed by Azam et al.^40^. Assume that $\zeta ,\xi \in \mathbb {C}$ and that $\mathbb {C}$ is the collection of complex numbers. According to the listed below, define a partial order $\precsim$ on $\mathbb {C}$.

$$\begin{aligned} \zeta \precsim \xi \ \ \Leftrightarrow \ \ \textbf{Re}(\zeta )\le \textbf{Re}(\xi ) \ \ \text {and} \ \ \textbf{Im}(\zeta )\le \textbf{Im}(\xi ). \end{aligned}$$

It follows that $\zeta \precsim \xi$, if one of the below mentioned assertions is gratified:

($$\mathscr {C}_1$$)
$$\textbf{Re}(\zeta )=\textbf{Re}(\xi )$$ and $$\textbf{Im}(\zeta )<\textbf{Im}(\xi )$$,
($$\mathscr {C}_2$$)
$$\textbf{Re}(\zeta )<\textbf{Re}(\xi )$$ and $$\textbf{Im}(\zeta )=\textbf{Im}(\xi )$$,
($$\mathscr {C}_3$$)
$$\textbf{Re}(\zeta )<\textbf{Re}(\xi )$$ and $$\textbf{Im}(\zeta )<\textbf{Im}(\xi )$$,
($$\mathscr {C}_4$$)
$$\textbf{Re}(\zeta )=\textbf{Re}(\xi )$$ and $$\textbf{Im}(\zeta )=\textbf{Im}(\xi )$$.

Particularly, we call $\zeta \precnsim \xi$ if $\zeta \ne \xi$ and among one of $(\mathscr {C}_2),(\mathscr {C}_3)$ and $(\mathscr {C}_4)$ is gratified and we termed as $\zeta \prec \xi$ if only $(\mathscr {C}_3)$ is gratified. The below mentioned assertions gratify:

1. If $$0\precsim \zeta \precnsim \xi$$, then $$|\zeta |<|\xi |$$.
2. If $\zeta \precsim \xi$ and $\xi \prec \varsigma$, then $\zeta \prec \varsigma$.

### Definition 1.1

^40^Let $\mathscr {M}$ be a nonempty set. The mapping ${d}:\mathscr {M}\times \mathscr {M}\rightarrow \mathbb {C}$, gratifies

1. $$0\precsim {d}(\texttt {x},\texttt {y}) \ \text {and} \ {d}(\texttt {x},\texttt {y})=0\Leftrightarrow \texttt {x}=\texttt {y} \ \forall \ \texttt {x},\texttt {y}\in \mathscr {M}$$;
2. $${d}(\texttt {x},\texttt {y})={d}(\texttt {y},\texttt {x}), \ \forall \ \texttt {x},\texttt {y}\in \mathscr {M}$$;
3. $${d}(\texttt {x},\texttt {y})\precsim {d}(\texttt {x},\mathbbm {z})+{d}(\mathbbm {z},\texttt {y}), \ \forall \ \texttt {x},\texttt {y},\mathbb {z}\in \mathscr {M}$$.

Then *d* is called a complex-valued metric on $\mathscr {M}$, and $(\mathscr {M},{d})$ is called a complex-valued metric space.

### Theorem 1.1

^40^ Let $(\mathscr {M},{d})$ be a complex-valued metric space and let mappings $\mathscr {A},\mathscr {B}:\mathscr {M}\rightarrow \mathscr {M}$ gratify

$$\begin{aligned} {d}(\mathscr {A}{} \texttt {x},\mathscr {B}{} \texttt {y})\precsim \nu {d}(\texttt {x},\texttt {y})+\frac{\varrho {d}(\texttt {x},\mathscr {A}{} \texttt {y}){d}(\texttt {y},\mathscr {B}{} \texttt {y})}{1+{d}(\texttt {x},\texttt {y})} \end{aligned}$$

where $\texttt {x},\texttt {y}\in \mathscr {M}$ and $\nu ,\varrho$ are nonnegative reals having the condition $\nu +\varrho <1$. Then $\mathscr {A}$ and $\mathscr {B}$ have a unique common fixed point.

If we take $\varrho =0$ in Theorem.1.1, we get *contractive mapping theorem* in the setting of complex-valued metric space.

### Theorem 1.2

Let $(\mathscr {M},{d})$ be a complex-valued metric space and let mapping $\mathscr {W}:\mathscr {M}\rightarrow \mathscr {M}$ gratify

$$\begin{aligned} {d}(\mathscr {W}{} \texttt {x},\mathscr {W}{} \texttt {y})\precsim \nu {d}(\texttt {x},\texttt {y}) \end{aligned}$$

where $\texttt {x},\texttt {y}\in \mathscr {M}$ and $0<\nu <1$. Then $\mathscr {W}$ has a unique fixed point.

In this study, we take into account the following time-delayed fractional-order CVNNs system:

1.1 $$\begin{aligned} ^{C }\mathscr {D}_{{\mathfrak {t}}_{0}}^{\gamma ,\mu }{z}_{k}({\mathfrak {t}})=-c_{k}{z}_{k}({\mathfrak {t}})+{\displaystyle \sum }_{\mathfrak {j}=1}^{n}\mathfrak {s}_{k\mathfrak {j}}\varphi _{\mathfrak {j}}({z}_{\mathfrak {j}}({\mathfrak {t}}))+{\sum }_{\mathfrak {j}=1}^{n}\mathfrak {p}_{k\mathfrak {j}}\varphi _{\mathfrak {j}}({z}_{\mathfrak {j}}({\mathfrak {t}}-{\tau }))+\Upsilon _{k}; \ k=1,2,3,\ldots n \end{aligned}$$

The compatible vector form is as follows:

1.2 $$\begin{aligned} ^{C }\mathscr {D}_{{\mathfrak {t}}_{0}}^{\gamma ,\mu }{z}({\mathfrak {t}})=-\mathscr {C}{z}({\mathfrak {t}})+\mathcal {G}\varphi ({z}({\mathfrak {t}}))+\mathcal {H}\varphi ({z}({\mathfrak {t}}-{\tau }))+\Upsilon , \end{aligned}$$

for ${\mathfrak {t}}\in [{\mathfrak {t}}_{0},{\mathfrak {t}}_{\varphi }]$, where $^{C }\mathscr {D}_{{\mathfrak {t}}_{0}}^{\gamma ,\mu }$ is the generalized Caputo fractional derivative with parameters $0<\gamma <1$ and $\mu >0$ as in^41,42^. Moreover, ${z}({\mathfrak {t}})=({z}_{1}({\mathfrak {t}}),{z}_{2}({\mathfrak {t}}),{z}_{3}({\mathfrak {t}}),\ldots {z}_{n}({\mathfrak {t}}))^{\mathbb {T}}\in \mathbb {C}^{n}$ is the state vector, and $\varphi ({z}({\mathfrak {t}}))=(\varphi _{1}({z}_{1}({\mathfrak {t}})),\varphi _{2}({z}_{2}({\mathfrak {t}})),$

$\varphi _{3}({z}_{3}({\mathfrak {t}}))\ldots \varphi _{n}({z}_{n}({\mathfrak {t}})))^{\mathbb {T}}: \mathbb {C}^{n}\rightarrow \mathbb {C}^{n}$ is the neuron activation function. $\mathscr {C}={\mathbbm {d}}{\mathbbm {i}}{\mathbbm {a}}{\mathbbm {g}}(c_{1}, c_{2}, c_{3},\ldots c_{n})\in \mathbbm {R}^{n\times n}$ with $c_{k}>0$ where $k=1,2,3,\cdots , n$ is the self-feedback connection weighted matrix. $\mathcal {G}=(\mathfrak {s}_{k\mathfrak {j}})_{n\times n}\in \mathbb {C}^{n\times n}$ and $\mathcal {H}=(\mathfrak {p}_{k\mathfrak {j}})_{n\times n}\in \mathbb {C}^{n\times n}$ represent the connection between the $\mathfrak {j}^{th}$ neuron and the $k^{th}$ neuron at time ${\mathfrak {t}}$ and constant time delay ${\mathfrak {t}}-{\tau }$ respectively. $\Upsilon =(\Upsilon _{1},\Upsilon _{2},\Upsilon _{3},\ldots \Upsilon _{n})^{\mathbb {T}}\in \mathbb {C}^{n}$ is the external input vector.

In this instance, the initial conditions connected to (1.1) have the following form:

1.3 $$\begin{aligned} {z}_{k}(\mathfrak {q})=\Psi _{k}(\mathfrak {q})+\mathfrak {i}\Theta _{k}(\mathfrak {q}), \ \mathfrak {q}\in [-{\tau },0], \ k=1,2,3\ldots n, \end{aligned}$$

here $\Psi _{k}(\mathfrak {q}),\Theta _{k}(\mathfrak {q})\in \mathbb {C}([-{\tau },0],\mathbb {R}^{n}), k=1,2,3\ldots n$. Moreover, $\mathbb {C}([-{\tau },0],\mathbb {R}^{n})$ refers to the complex-valued metric space which consists continuous *n*-real vector functions provided on $[-{\tau },0]$,

$$\begin{aligned} {||\Psi ({\mathfrak {t}})||={\sup }_{{\mathfrak {t}}\in (-{\tau },0]}{\sum }_{k=1}^{n}{e }^{{\mathfrak {t}}(k-1)}|\Psi _{k}({\mathfrak {t}})|} \end{aligned}$$

and

$$\begin{aligned} {||\Theta ({\mathfrak {t}})||={\sup }_{{\mathfrak {t}}\in (-{\tau },0]}{\sum }_{k=1}^{n}{e }^{{\mathfrak {t}}(k-1)}|\Theta _{k}({\mathfrak {t}})|.} \end{aligned}$$

### Definition 1.2

The solution of the system (1.2) is considered to be stable if for any $\epsilon >0$, there is a $\vartheta ({\mathfrak {t}}_{0},\epsilon )>0$ in such a way that ${\mathfrak {t}}\ge {\mathfrak {t}}_{0}\ge 0$, $||\varkappa ({\mathfrak {t}})-\lambda ({\mathfrak {t}})||<\vartheta$ that suggests $||\chi _{1}({\mathfrak {t}},{\mathfrak {t}}_{0},\varkappa )-\chi ({\mathfrak {t}},{\mathfrak {t}}_{0},\lambda )||<\epsilon$. If the aforementioned $\vartheta$ is not dependent on ${\mathfrak {t}}_{0}$, it will become uniformly stable.

We adopt the subsequent assumptions in order to obtain the main results.

**Assumption** $$\mathcal {A}$$:

$$\begin{aligned} \vec {c}= & {} \min (|1-c_{\max }|,c_{\min }); \ c_{\min }=\min _{\forall \mathfrak {j}}\{c_{\mathfrak {j}}^{2}\}; \ c_{\max }=\frac{{\mathfrak {t}}^{\gamma \mu }}{{e }^{\gamma }\Gamma (\gamma +1)}\max _{\forall \mathfrak {j}}\{c_{\mathfrak {j}}\};\\ ||\mathfrak {a}^{\star }||= & {} {\sum }_{\mathfrak {j}=1}^{n}|\mathfrak {a}_{\mathfrak {j}}^{\star }|={\sum }_{\mathfrak {j}=1}^{n}\max _{\forall \ell }\{|\mathfrak {s}_{\mathfrak {j}\ell }|\theta _{\ell }\}; \ ||\mathfrak {b}^{\star }||={\sum }_{\mathfrak {j}=1}^{n}|\mathfrak {b}_{\mathfrak {j}}^{\star }|={\sum }_{\mathfrak {j}=1}^{n}\max _{\forall \ell }\{|\mathfrak {p}_{\mathfrak {j}\ell }|\theta _{\ell }\};\\ ||\mathfrak {a}_{1}^{\star }+\mathfrak {a}_{3}^{\star }||= & {} \frac{{\mathfrak {t}}^{\gamma \mu }}{{e }^{\gamma }\Gamma (\gamma +1)}{\sum }_{\mathfrak {j}=1}^{n}[\mathfrak {a}_{1\mathfrak {j}}^{\star }+\mathfrak {a}_{3\mathfrak {j}}^{\star }]; \ \ ||\mathfrak {b}_{1}^{\star }+\mathfrak {b}_{3}^{\star }||=\frac{{\mathfrak {t}}^{\gamma \mu }}{{e }^{\gamma }\Gamma (\gamma +1)}{\sum }_{\mathfrak {j}=1}^{n}[\mathfrak {b}_{1\mathfrak {j}}^{\star }+\mathfrak {b}_{3\mathfrak {j}}^{\star }]\\ ||\mathfrak {a}_{2}^{\star }+\mathfrak {a}_{4}^{\star }||= & {} \frac{{\mathfrak {t}}^{\gamma \mu }}{{e }^{\gamma }\Gamma (\gamma +1)}{\sum }_{\mathfrak {j}=1}^{n}[\mathfrak {a}_{2\mathfrak {j}}^{\star }+\mathfrak {a}_{4\mathfrak {j}}^{\star }]; \ \ ||\mathfrak {b}_{2}^{\star }+\mathfrak {b}_{4}^{\star }||=\frac{{\mathfrak {t}}^{\gamma \mu }}{{e }^{\gamma }\Gamma (\gamma +1)}{\sum }_{\mathfrak {j}=1}^{n}[\mathfrak {b}_{2\mathfrak {j}}^{\star }+\mathfrak {b}_{4\mathfrak {j}}^{\star }]\\ ||\mathfrak {q}_{1}^{\star }+\mathfrak {q}_{3}^{\star }||= & {} \frac{{\mathfrak {t}}^{\gamma \mu }}{{e }^{\gamma }\Gamma (\gamma +1)}{\sum }_{\mathfrak {j}=1}^{n}[\mathfrak {q}_{1\mathfrak {j}}^{\star }+\mathfrak {q}_{3\mathfrak {j}}^{\star }]; \ \ ||\mathfrak {q}_{2}^{\star }+\mathfrak {q}_{4}^{\star }||=\frac{{\mathfrak {t}}^{\gamma \mu }}{{e }^{\gamma }\Gamma (\gamma +1)}{\sum }_{\mathfrak {j}=1}^{n}[\mathfrak {q}_{2\mathfrak {j}}^{\star }+\mathfrak {q}_{4\mathfrak {j}}^{\star }];\\ ||{\mathfrak {t}}_{2}^{\star }+{\mathfrak {t}}_{4}^{\star }||= & {} \frac{{\mathfrak {t}}^{\gamma \mu }}{{e }^{\gamma }\Gamma (\gamma +1)}{\sum }_{\mathfrak {j}=1}^{n}[{\mathfrak {t}}_{2\mathfrak {j}}^{\star }+{\mathfrak {t}}_{4\mathfrak {j}}^{\star }]; \ \ ||{\mathfrak {t}}_{1}^{\star }+{\mathfrak {t}}_{3}^{\star }||=\frac{{\mathfrak {t}}^{\gamma \mu }}{{e }^{\gamma }\Gamma (\gamma +1)}{\sum }_{\mathfrak {j}=1}^{n}[{\mathfrak {t}}_{1\mathfrak {j}}^{\star }+{\mathfrak {t}}_{3\mathfrak {j}}^{\star }] \end{aligned}$$

**Assumption** $\mathcal {B}$: Let ${z}=\texttt {x}+\mathfrak {i}{} \texttt {y}$ where $\mathfrak {i}$ denotes the imagenary unit, that is $\mathfrak {i}=\sqrt{-1}$. Moreover, $\varphi _{\mathfrak {j}}(u)$ and $\varphi _{\mathfrak {j}}(u(\rho -{\tau }))$ by separating it into its real as well as imagined portions, it could potentially be demonstrated as

$$\begin{aligned} \varphi _{\mathfrak {j}}(u)= & {} \varphi _{\mathfrak {j}}^{R}(\texttt {x},\texttt {y})+\mathfrak {i}\varphi _{\mathfrak {j}}^{I}(\texttt {x},\texttt {y});\\ \varphi _{\mathfrak {j}}(u(\rho -{\tau }))= & {} \varphi _{\mathfrak {j}}^{R}(\texttt {x}(\rho -{\tau }),\texttt {y}(\rho -{\tau }))+\mathfrak {i}\varphi _{\mathfrak {j}}^{I}(\texttt {x}(\rho -{\tau }),\texttt {y}(\rho -{\tau })); \end{aligned}$$

where $\varphi _{\mathfrak {j}}^{R}(.,.)$ is maps from $\mathbbm {R}^{2}$ to $\mathbbm {R}$; $\varphi _{\mathfrak {j}}^{I}(.,.)$ maps from $\mathbbm {R}^{2}$ to $\mathbbm {R}$; $\varphi _{\mathfrak {j}}^{R}(.,.)$ maps from $\mathbbm {R}^{2}$ to $\mathbbm {R}$ and $\varphi _{\mathfrak {j}}^{I}(.,.)$ maps from $\mathbbm {R}^{2}$ to $\mathbbm {R}$. To make formulas simpler, $\texttt {x}(\rho -{\tau })$ and $\texttt {y}(\rho -{\tau })$ despite being stated as $\texttt {x}_{{\tau }}$ and $\texttt {y}_{{\tau }}$ appropriately.

- In addition, the partial derivatives of $\varphi _{\mathfrak {j}}(.,.)$ regarded to $\texttt {x},\texttt {y}:\partial \varphi _{\mathfrak {j}}^{R}/\partial \texttt {x}, \partial \varphi _{\mathfrak {j}}^{R}/\partial \texttt {y}, \partial \varphi _{\mathfrak {j}}^{I}/\partial \texttt {x}$ and $\partial \varphi _{\mathfrak {j}}^{I}/\partial \texttt {y}$ are exist and they are continuous.
- $\partial \varphi _{\mathfrak {j}}^{R}/\partial \texttt {x}, \partial \varphi _{\mathfrak {j}}^{R}/\partial \texttt {y}, \partial \varphi _{\mathfrak {j}}^{I}/\partial \texttt {x}$ and $\partial \varphi _{\mathfrak {j}}^{I}/\partial \texttt {y}$ are bounded, *i*.*e*., 
  $$\begin{aligned}{} & {} \Big |\frac{\partial \varphi _{\mathfrak {j}}^{R}}{\partial \texttt {x}}\Big |\le \theta _{\mathfrak {j}}^{RR}, \ \ \ \Big |\frac{\partial \varphi _{\mathfrak {j}}^{R}}{\partial \texttt {y}}\Big |\le \theta _{\mathfrak {j}}^{RI},\\{} & {} \Big |\frac{\partial \varphi _{\mathfrak {j}}^{I}}{\partial \texttt {x}}\Big |\le \theta _{\mathfrak {j}}^{IR}, \ \ \ \Big |\frac{\partial \varphi _{\mathfrak {j}}^{I}}{\partial \texttt {y}}\Big |\le \theta _{\mathfrak {j}}^{II}, \end{aligned}$$
  where $\theta _{\mathfrak {j}}^{RR},\theta _{\mathfrak {j}}^{RI},\theta _{\mathfrak {j}}^{IR}$ and $\theta _{\mathfrak {j}}^{II}$ are non-negative constant numbers.
- In addition, the partial derivatives(with time delay) $\partial \varphi _{\mathfrak {j}}^{R}/\partial \texttt {x}, \partial \varphi _{\mathfrak {j}}^{R}/\partial \texttt {y}, \partial \varphi _{\mathfrak {j}}^{I}/\partial \texttt {x}$ and $\partial \varphi _{\mathfrak {j}}^{I}/\partial \texttt {y}$, they’re deemed as bounded. In other words, additionally, there are some numbers that are consistently non-negative. $\omega _{\mathfrak {j}}^{RR},\omega _{\mathfrak {j}}^{RI},\omega _{\mathfrak {j}}^{IR}$ and $\omega _{\mathfrak {j}}^{II}$ so that
  $$\begin{aligned}{} & {} \Big |\frac{\partial \varphi _{\mathfrak {j}}^{R}}{\partial \texttt {x}}\Big |\le \omega _{\mathfrak {j}}^{RR}, \ \ \ \Big |\frac{\partial \varphi _{\mathfrak {j}}^{R}}{\partial \texttt {y}}\Big |\le \omega _{\mathfrak {j}}^{RI},\\{} & {} \Big |\frac{\partial \varphi _{\mathfrak {j}}^{I}}{\partial \texttt {x}}\Big |\le \omega _{\mathfrak {j}}^{IR}, \ \ \ \Big |\frac{\partial \varphi _{\mathfrak {j}}^{I}}{\partial \texttt {y}}\Big |\le \omega _{\mathfrak {j}}^{II}. \end{aligned}$$
  We can infer the following from the mean value hypothesis regarding multi-variable features:
  1.4 $$\begin{aligned}{} & {} |\varphi _{\mathfrak {j}}^{R}(\texttt {x}^{\prime },\texttt {y}^{\prime })-\varphi _{\mathfrak {j}}^{R}(\texttt {x},\texttt {y})|\le \theta _{\mathfrak {j}}^{RR}|\texttt {x}^{\prime }-\texttt {x}|+\theta _{\mathfrak {j}}^{RI}|\texttt {y}^{\prime }-\texttt {y}|; \nonumber \\{} & {} |\varphi _{\mathfrak {j}}^{I}(\texttt {x}^{\prime },\texttt {y}^{\prime })-\varphi _{\mathfrak {j}}^{I}(\texttt {x},\texttt {y})|\le \theta _{\mathfrak {j}}^{IR}|\texttt {x}^{\prime }-\texttt {x}|+\theta _{\mathfrak {j}}^{II}|\texttt {y}^{\prime }-\texttt {y}|; \nonumber \\{} & {} |\varphi _{\mathfrak {j}}^{R}(\texttt {x}^{\prime }_{{\tau }},\texttt {y}^{\prime }_{{\tau }})-\varphi _{\mathfrak {j}}^{R}(\texttt {x}_{{\tau }},\texttt {y}_{{\tau }})|\le \omega _{\mathfrak {j}}^{RR}|\texttt {x}^{\prime }_{{\tau }}-\texttt {x}_{{\tau }}|+\omega _{\mathfrak {j}}^{RI}|\texttt {y}^{\prime }_{{\tau }}-\texttt {y}_{{\tau }}|; \nonumber \\{} & {} |\varphi _{\mathfrak {j}}^{I}(\texttt {x}^{\prime }_{{\tau }},\texttt {y}^{\prime }_{{\tau }})-\varphi _{\mathfrak {j}}^{I}(\texttt {x}_{{\tau }},\texttt {y}_{{\tau }})|\le \omega _{\mathfrak {j}}^{IR}|\texttt {x}^{\prime }_{{\tau }}-\texttt {x}_{{\tau }}|+\omega _{\mathfrak {j}}^{II}|\texttt {y}^{\prime }_{{\tau }}-\texttt {y}_{{\tau }}|, \end{aligned}$$
  where $$\texttt {x},\texttt {x}^{\prime },\texttt {y},\texttt {y}^{\prime }\in \mathbbm {R}^{w}.$$

**Assumption** $\mathcal {C}$: $\varphi _{\mathfrak {j}}(.)$ gratify the Lipschitz assertions, that is, for any $l,k\in \mathcal {C},$ there is non-negative constant $\theta _{\mathfrak {j}}$ such a way that

1.5 $$\begin{aligned} ||\varphi _{\mathfrak {j}}(l)-\varphi _{\mathfrak {j}}(k)||\le \theta _{\mathfrak {j}}||l-k||. \end{aligned}$$

## Uniform stability result of generalized Caputo fractional-order complex-valued neural networks

In the current section, we construct a necessary condition for the generalized Caputo fractional-order complex-valued neural networks with time delays to be uniformly stable. The existence and uniqueness results are then obtained through the contraction mapping theorem in complex-valued metric space.

### Theorem 2.1

If Assumption $\mathcal {A}$ and $\mathcal {B}$ hold, then (1.1) is uniformly stable.

### Proof

By splitting the real and imaginary components of the generalized Caputo fractional-order complex-valued neural network, we obtain,

2.1 $$\begin{aligned} ^{C }\mathscr {D}_{{\mathfrak {t}}_{0}}^{\gamma ,\mu }{} \texttt {x}({\mathfrak {t}})&=-\mathscr {C}{} \texttt {x}({\mathfrak {t}})+\mathcal {G}^{R}\varphi ^{R}(\texttt {x},\texttt {y})-\mathcal {G}^{I}\varphi ^{I}(\texttt {x},\texttt {y})\nonumber \\&\quad +\mathcal {H}^{R}\varphi ^{R}(\texttt {x}({\mathfrak {t}}-{\tau }),\texttt {y}({\mathfrak {t}}-{\tau }))-\mathcal {H}^{I}\varphi ^{I}(\texttt {x}({\mathfrak {t}}-{\tau }),\texttt {y}({\mathfrak {t}}-{\tau }))+\Upsilon ^{R} \end{aligned}$$

2.2 $$\begin{aligned} ^{C }\mathscr {D}_{{\mathfrak {t}}_{0}}^{\gamma ,\mu }{} \texttt {y}({\mathfrak {t}})&=-\mathscr {C}{} \texttt {y}({\mathfrak {t}})+\mathcal {G}^{I}\varphi ^{R}(\texttt {x},\texttt {y})-\mathcal {G}^{R}\varphi ^{I}(\texttt {x},\texttt {y})\nonumber \\&\quad +\mathcal {H}^{I}\varphi ^{R}(\texttt {x}({\mathfrak {t}}-{\tau }),\texttt {y}({\mathfrak {t}}-{\tau }))-\mathcal {H}^{R}\varphi ^{I}(\texttt {x}({\mathfrak {t}}-{\tau }),\texttt {y}({\mathfrak {t}}-{\tau }))+\Upsilon ^{I} \end{aligned}$$

Equations (2.1) and (2.2) can be rewritten as:

2.3 $$\begin{aligned} ^{C }\mathscr {D}_{{\mathfrak {t}}_{0}}^{\gamma ,\mu }{} \texttt {x}_{\mathfrak {j}}({\mathfrak {t}})&=-c_{\mathfrak {j}}{} \texttt {x}_{\mathfrak {j}}({\mathfrak {t}})+{\sum }_{\ell =1}^{n}\mathfrak {s}_{\mathfrak {j}\ell }^{R}\varphi _{\ell }^{R}(\texttt {x}_{\ell },\texttt {y}_{\ell })-{\sum }_{\ell =1}^{n}\mathfrak {s}_{\mathfrak {j}\ell }^{I}\varphi _{\ell }^{I}(\texttt {x}_{\ell },\texttt {y}_{\ell })\nonumber \\&\quad +{\sum }_{\ell =1}^{n}\mathfrak {p}_{\mathfrak {j}\ell }^{R}\varphi _{\ell }^{R}(\texttt {x}_{\ell {\tau }},\texttt {y}_{\ell {\tau }})-{\sum }_{\ell =1}^{n}\mathfrak {p}_{\mathfrak {j}\ell }^{I}\varphi _{\ell }^{I}(\texttt {x}_{\ell {\tau }},\texttt {y}_{\ell {\tau }})+\Upsilon _{\mathfrak {j}}^{R} \end{aligned}$$

2.4 $$\begin{aligned} ^{C }\mathscr {D}_{{\mathfrak {t}}_{0}}^{\gamma ,\mu }{} \texttt {y}_{\mathfrak {j}}({\mathfrak {t}})&=-c_{\mathfrak {j}}{} \texttt {y}_{\mathfrak {j}}({\mathfrak {t}})+{\sum }_{\ell =1}^{n}\mathfrak {s}_{\mathfrak {j}\ell }^{I}\varphi _{\ell }^{R}(\texttt {x}_{\ell },\texttt {y}_{\ell })+{\sum }_{\ell =1}^{n}\mathfrak {s}_{\mathfrak {j}\ell }^{R}\varphi _{\ell }^{I}(\texttt {x}_{\ell },\texttt {y}_{\ell })\nonumber \\&\quad +{\sum }_{\ell =1}^{n}\mathfrak {p}_{\mathfrak {j}\ell }^{I}\varphi _{\ell }^{R}(\texttt {x}_{\ell {\tau }},\texttt {y}_{\ell {\tau }})+{\sum }_{\ell =1}^{n}\mathfrak {p}_{\mathfrak {j}\ell }^{R}\varphi _{\ell }^{I}(\texttt {x}_{\ell {\tau }},\texttt {y}_{\ell {\tau }})+\Upsilon _{\mathfrak {j}}^{I} \end{aligned}$$

Let us assume ${z}=\texttt {x}+\mathfrak {i}{} \texttt {y}$ and ${z}^{\prime }=\texttt {x}^{\prime }+\mathfrak {i}{} \texttt {y}^{\prime }$ having the conditions $\texttt {y}^{\prime }\ne \texttt {y}$, $\texttt {x}^{\prime }\ne \texttt {x}$. The two possible solutions of 1.1 are ${z}^{\prime }({\mathfrak {t}})=({z}_{1}({\mathfrak {t}}),{z}_{2}({\mathfrak {t}}),{z}_{3}({\mathfrak {t}})\ldots {z}_{n}({\mathfrak {t}}))$ and ${z}^{\prime }({\mathfrak {t}})=({z}^{\prime }_{1}({\mathfrak {t}}),{z}^{\prime }_{2}({\mathfrak {t}}),{z}^{\prime }_{3}({\mathfrak {t}})\ldots {z}^{\prime }_{n}({\mathfrak {t}}))$ having distinct initial conditions ${z}_{\hbar }^{\prime }(\mathfrak {q})=\Psi _{\hbar }^{\prime }(\mathfrak {q})+\mathfrak {i}\Theta _{\hbar }^{\prime }(\mathfrak {q})$, where $\Psi _{\hbar }^{\prime }(\mathfrak {q}),\Theta _{\hbar }^{\prime }(\mathfrak {q})\in \mathbb {C}([-{\tau },0],\mathbb {R}^{n})$, ${z}_{\hbar }(\mathfrak {q})=\Psi _{\hbar }(\mathfrak {q})+\mathfrak {i}\Theta _{\hbar }(\mathfrak {q})$, where $\Psi _{\hbar }(\mathfrak {q}),\Theta _{\hbar }(\mathfrak {q})\in \mathbb {C}([-{\tau },0],\mathbb {R}^{n}), \ \hbar \in n$. We have

2.5 $$\begin{aligned}&^{C }\mathscr {D}_{{\mathfrak {t}}_{0}}^{\gamma ,\mu }(\texttt {x}^{\prime }_{\mathfrak {j}}({\mathfrak {t}})-\texttt {x}_{\mathfrak {j}}({\mathfrak {t}}))\nonumber \\&\quad =-c_{\mathfrak {j}}(\texttt {x}^{\prime }_{\mathfrak {j}}({\mathfrak {t}})-\texttt {x}_{\mathfrak {j}}({\mathfrak {t}}))+{\sum }_{\ell =1}^{n}\mathfrak {s}_{\mathfrak {j}\ell }^{R}\bigg [\varphi _{\ell }^{R}(\texttt {x}_{\ell }^{\prime },\texttt {y}_{\ell }^{\prime })-\varphi _{\ell }^{R}(\texttt {x}_{\ell },\texttt {y}_{\ell })\bigg ]-{\sum }_{\ell =1}^{n}\mathfrak {s}_{\mathfrak {j}\ell }^{I}\bigg [\varphi _{\ell }^{I}(\texttt {x}_{\ell }^{\prime },\texttt {y}_{\ell }^{\prime })-\varphi _{\ell }^{I}(\texttt {x}_{\ell },\texttt {y}_{\ell })\bigg ]\nonumber \\&\quad +{\sum }_{\ell =1}^{n}\mathfrak {p}_{\mathfrak {j}\ell }^{R}\bigg [\varphi _{\ell }^{R}(\texttt {x}_{\ell {\tau }}^{\prime },\texttt {y}_{\ell {\tau }}^{\prime })-\varphi _{\ell }^{R}(\texttt {x}_{\ell {\tau }},\texttt {y}_{\ell {\tau }})\bigg ]-{\sum }_{\ell =1}^{n}\mathfrak {p}_{\mathfrak {j}\ell }^{I}\bigg [\varphi _{\ell }^{I}(\texttt {x}_{\ell {\tau }}^{\prime },\texttt {y}_{\ell {\tau }}^{\prime })-\varphi _{\ell }^{I}(\texttt {x}_{\ell {\tau }},\texttt {y}_{\ell {\tau }})\bigg ] \end{aligned}$$

The integral equation below is equal to the previous equation (2.5),

2.6 $$\begin{aligned} \texttt {x}^{\prime }_{\mathfrak {j}}({\mathfrak {t}})-\texttt {x}_{\mathfrak {j}}({\mathfrak {t}})&=\Psi ^{\prime }_{\mathfrak {j}}(0)-\Psi _{\mathfrak {j}}(0)+\frac{\mu ^{1-\gamma }}{\Gamma (\gamma )}\int _{0}^{{\mathfrak {t}}}\mathfrak {q}^{\mu -1}({\mathfrak {t}}^{\mu }-\mathfrak {q}^{\mu })^{\gamma -1}\nonumber \\&\quad \times \biggl [-c_{\mathfrak {j}}(\texttt {x}^{\prime }_{\mathfrak {j}}(\mathfrak {q})-\texttt {x}_{\mathfrak {j}}(\mathfrak {q}))+{\sum }_{\ell =1}^{n}\mathfrak {s}_{\mathfrak {j}\ell }^{R}\bigg [\varphi _{\ell }^{R}(\texttt {x}_{\ell }^{\prime },\texttt {y}_{\ell }^{\prime })-\varphi _{\ell }^{R}(\texttt {x}_{\ell },\texttt {y}_{\ell })\bigg ]\nonumber \\&\quad -{\sum }_{\ell =1}^{n}\mathfrak {s}_{\mathfrak {j}\ell }^{I}\bigg [\varphi _{\ell }^{I}(\texttt {x}_{\ell }^{\prime },\texttt {y}_{\ell }^{\prime })-\varphi _{\ell }^{I}(\texttt {x}_{\ell },\texttt {y}_{\ell })\bigg ] \end{aligned}$$

2.7 $$\begin{aligned}&\quad +{\sum }_{\ell =1}^{n}\mathfrak {p}_{\mathfrak {j}\ell }^{R}\bigg [\varphi _{\ell }^{R}(\texttt {x}_{\ell {\tau }}^{\prime },\texttt {y}_{\ell {\tau }}^{\prime })-\varphi _{\ell }^{R}(\texttt {x}_{\ell {\tau }},\texttt {y}_{\ell {\tau }})\bigg ]-{\sum }_{\ell =1}^{n}\mathfrak {p}_{\mathfrak {j}\ell }^{I}\bigg [\varphi _{\ell }^{I}(\texttt {x}_{\ell {\tau }}^{\prime },\texttt {y}_{\ell {\tau }}^{\prime })-\varphi _{\ell }^{I}(\texttt {x}_{\ell {\tau }},\texttt {y}_{\ell {\tau }})\bigg ] \biggr ]. \end{aligned}$$

Which implies,

$$\begin{aligned} |\texttt {x}^{\prime }_{\mathfrak {j}}({\mathfrak {t}})-\texttt {x}_{\mathfrak {j}}({\mathfrak {t}})|&=|\Psi ^{\prime }_{\mathfrak {j}}(0)-\Psi _{\mathfrak {j}}(0)|+\frac{\mu ^{1-\gamma }}{\Gamma (\gamma )}\int _{0}^{{\mathfrak {t}}}\mathfrak {q}^{\mu -1}({\mathfrak {t}}^{\mu }-\mathfrak {q}^{\mu })^{\gamma -1}\\&\quad \times \biggl [c_{\mathfrak {j}}|(\texttt {x}^{\prime }_{\mathfrak {j}}(\mathfrak {q})-\texttt {x}_{\mathfrak {j}}(\mathfrak {q}))|+{\sum }_{\ell =1}^{n}|\mathfrak {s}_{\mathfrak {j}\ell }^{R}||\varphi _{\ell }^{R}(\texttt {x}_{\ell }^{\prime },\texttt {y}_{\ell }^{\prime })-\varphi _{\ell }^{R}(\texttt {x}_{\ell },\texttt {y}_{\ell })|\\&\quad +{\sum }_{\ell =1}^{n}|\mathfrak {s}_{\mathfrak {j}\ell }^{I}||\varphi _{\ell }^{I}(\texttt {x}_{\ell }^{\prime },\texttt {y}_{\ell }^{\prime })-\varphi _{\ell }^{I}(\texttt {x}_{\ell },\texttt {y}_{\ell })|\\&\quad +{\sum }_{\ell =1}^{n}|\mathfrak {p}_{\mathfrak {j}\ell }^{R}||\varphi _{\ell }^{R}(\texttt {x}_{\ell {\tau }}^{\prime },\texttt {y}_{\ell {\tau }}^{\prime })-\varphi _{\ell {\tau }}^{R}(\texttt {x}_{\ell {\tau }},\texttt {y}_{\ell })|+{\sum }_{\ell =1}^{n}|\mathfrak {p}_{\mathfrak {j}\ell }^{I}||\varphi _{\ell }^{I}(\texttt {x}_{\ell {\tau }}^{\prime },\texttt {y}_{\ell {\tau }}^{\prime })-\varphi _{\ell }^{I}(\texttt {x}_{\ell {\tau 
}},\texttt {y}_{\ell {\tau }})| \biggr ]\\&\precsim |\Psi ^{\prime }_{\mathfrak {j}}(0)-\Psi _{\mathfrak {j}}(0)|+c_{\mathfrak {j}}\frac{\mu ^{1-\gamma }}{\Gamma (\gamma )}\int _{0}^{{\mathfrak {t}}}\mathfrak {q}^{\mu -1}({\mathfrak {t}}^{\mu }-\mathfrak {q}^{\mu })^{\gamma -1}|(\texttt {x}^{\prime }_{\mathfrak {j}}(\mathfrak {q})-\texttt {x}_{\mathfrak {j}}(\mathfrak {q}))|d\mathfrak {q}\\&\quad +{\sum }_{\ell =1}^{n}|\mathfrak {s}_{\mathfrak {j}\ell }^{R}|\frac{\mu ^{1-\gamma }}{\Gamma (\gamma )}\int _{0}^{{\mathfrak {t}}}\mathfrak {q}^{\mu -1}({\mathfrak {t}}^{\mu }-\mathfrak {q}^{\mu })^{\gamma -1}\big [\theta _{\ell }^{RR}|\texttt {x}^{\prime }_{\ell }(\mathfrak {q})-\texttt {x}_{\ell }(\mathfrak {q})|+\theta _{\ell }^{RI}|\texttt {y}^{\prime }_{\ell }(\mathfrak {q})-\texttt {y}_{\ell }(\mathfrak {q})|\big ]d\mathfrak {q}\\&\quad +{\sum }_{\ell =1}^{n}|\mathfrak {s}_{\mathfrak {j}\ell }^{I}|\frac{\mu ^{1-\gamma }}{\Gamma (\gamma )}\int _{0}^{{\mathfrak {t}}}\mathfrak {q}^{\mu -1}({\mathfrak {t}}^{\mu }-\mathfrak {q}^{\mu })^{\gamma -1}\big [\theta _{\ell }^{IR}|\texttt {x}^{\prime }_{\ell }(\mathfrak {q})-\texttt {x}_{\ell }(\mathfrak {q})|+\theta _{\ell }^{II}|\texttt {y}^{\prime }_{\ell }(\mathfrak {q})-\texttt {y}_{\ell }(\mathfrak {q})|\big ]d\mathfrak {q}\\&\quad +{\sum }_{\ell =1}^{n}|\mathfrak {p}_{\mathfrak {j}\ell }^{R}|\frac{\mu ^{1-\gamma }}{\Gamma (\gamma )}\int _{0}^{{\mathfrak {t}}}\mathfrak {q}^{\mu -1}({\mathfrak {t}}^{\mu }-\mathfrak {q}^{\mu })^{\gamma -1}[\omega _{\ell }^{RR}|\texttt {x}^{\prime }_{\ell {\tau }}(\mathfrak {q})-\texttt {x}_{\ell {\tau }}(\mathfrak {q})|+\omega _{\ell }^{RI}|\texttt {y}^{\prime }_{\ell {\tau }}(\mathfrak {q})-\texttt {y}_{\ell {\tau }}(\mathfrak {q})|\big ]d\mathfrak {q}\\&\quad +{\sum }_{\ell =1}^{n}|\mathfrak {p}_{\mathfrak {j}\ell }^{I}|\frac{\mu ^{1-\gamma }}{\Gamma (\gamma )}\int _{0}^{{\mathfrak {t}}}\mathfrak {q}^{\mu -1}({\mathfrak {t}}^{\mu }-\mathfrak {q}^{\mu })^{\gamma -1}[\omega _{\ell }^{IR}|\texttt {x}^{\prime }_{\ell {\tau }}(\mathfrak {q})-\texttt {x}_{\ell {\tau }}(\mathfrak {q})|+\omega _{\ell }^{II}|\texttt {y}^{\prime }_{\ell {\tau }}(\mathfrak {q})-\texttt {y}_{\ell {\tau }}(\mathfrak {q})|\big ]d\mathfrak {q}\\ \end{aligned}$$

$$\begin{aligned}&\precsim |\Psi ^{\prime }_{\mathfrak {j}}(0)-\Psi _{\mathfrak {j}}(0)|+c_{\mathfrak {j}}\frac{\mu ^{1-\gamma }}{\Gamma (\gamma )}\int _{0}^{{\mathfrak {t}}}\mathfrak {q}^{\mu -1}({\mathfrak {t}}^{\mu }-\mathfrak {q}^{\mu })^{\gamma -1}|(\texttt {x}^{\prime }_{\mathfrak {j}}(\mathfrak {q})-\texttt {x}_{\mathfrak {j}}(\mathfrak {q}))|d\mathfrak {q}\\&\quad +{\sum }_{\ell =1}^{n}|\mathfrak {s}_{\mathfrak {j}\ell }^{R}|\theta _{\ell }^{RR}\frac{\mu ^{1-\gamma }}{\Gamma (\gamma )}\int _{0}^{{\mathfrak {t}}}\mathfrak {q}^{\mu -1}({\mathfrak {t}}^{\mu }-\mathfrak {q}^{\mu })^{\gamma -1}|\texttt {x}^{\prime }_{\ell }(\mathfrak {q})-\texttt {x}_{\ell }(\mathfrak {q})|d\mathfrak {q}\\&\quad +{\sum }_{\ell =1}^{n}|\mathfrak {s}_{\mathfrak {j}\ell }^{R}|\theta _{\ell }^{RI}\frac{\mu ^{1-\gamma }}{\Gamma (\gamma )}\int _{0}^{{\mathfrak {t}}}\mathfrak {q}^{\mu -1}({\mathfrak {t}}^{\mu }-\mathfrak {q}^{\mu })^{\gamma -1}|\texttt {y}^{\prime }_{\ell }(\mathfrak {q})-\texttt {y}_{\ell }(\mathfrak {q})|d\mathfrak {q}\\&\quad +{\sum }_{\ell =1}^{n}|\mathfrak {s}_{\mathfrak {j}\ell }^{I}|\theta _{\ell }^{IR}\frac{\mu ^{1-\gamma }}{\Gamma (\gamma )}\int _{0}^{{\mathfrak {t}}}\mathfrak {q}^{\mu -1}({\mathfrak {t}}^{\mu }-\mathfrak {q}^{\mu })^{\gamma -1}|\texttt {x}^{\prime }_{\ell }(\mathfrak {q})-\texttt {x}_{\ell }(\mathfrak {q})|d\mathfrak {q}\\&\quad +{\sum }_{\ell =1}^{n}|\mathfrak {s}_{\mathfrak {j}\ell }^{I}|\theta _{\ell }^{II}\frac{\mu ^{1-\gamma }}{\Gamma (\gamma )}\int _{0}^{{\mathfrak {t}}}\mathfrak {q}^{\mu -1}({\mathfrak {t}}^{\mu }-\mathfrak {q}^{\mu })^{\gamma -1}|\texttt {y}^{\prime }_{\ell }(\mathfrak {q})-\texttt {y}_{\ell }(\mathfrak {q})|d\mathfrak {q}\\&\quad +{\sum }_{\ell =1}^{n}|\mathfrak {p}_{\mathfrak {j}\ell }^{R}|\omega _{\ell }^{RR}\frac{\mu ^{1-\gamma }}{\Gamma (\gamma )}\int _{0}^{{\mathfrak {t}}}\mathfrak {q}^{\mu -1}({\mathfrak {t}}^{\mu }-\mathfrak {q}^{\mu })^{\gamma -1}|\texttt {x}^{\prime }_{\ell {\tau }}(\mathfrak {q})-\texttt {x}_{\ell {\tau }}(\mathfrak {q})|d\mathfrak {q}\\&\quad +{\sum }_{\ell =1}^{n}|\mathfrak {p}_{\mathfrak {j}\ell }^{R}|\omega _{\ell }^{RI}\frac{\mu ^{1-\gamma }}{\Gamma (\gamma )}\int _{0}^{{\mathfrak {t}}}\mathfrak {q}^{\mu -1}({\mathfrak {t}}^{\mu }-\mathfrak {q}^{\mu })^{\gamma -1}|\texttt {y}^{\prime }_{\ell {\tau }}(\mathfrak {q})-\texttt {y}_{\ell {\tau }}(\mathfrak {q})|d\mathfrak {q}\\&\quad +{\sum }_{\ell =1}^{n}|\mathfrak {p}_{\mathfrak {j}\ell }^{I}|\omega _{\ell }^{IR}\frac{\mu ^{1-\gamma }}{\Gamma (\gamma )}\int _{0}^{{\mathfrak {t}}}\mathfrak {q}^{\mu -1}({\mathfrak {t}}^{\mu }-\mathfrak {q}^{\mu })^{\gamma -1}|\texttt {x}^{\prime }_{\ell {\tau }}(\mathfrak {q})-\texttt {x}_{\ell {\tau }}(\mathfrak {q})|d\mathfrak {q} \end{aligned}$$

$$\begin{aligned}&\quad +{\sum }_{\ell =1}^{n}|\mathfrak {p}_{\mathfrak {j}\ell }^{I}|\omega _{\ell }^{II}\frac{\mu ^{1-\gamma }}{\Gamma (\gamma )}\int _{0}^{{\mathfrak {t}}}\mathfrak {q}^{\mu -1}({\mathfrak {t}}^{\mu }-\mathfrak {q}^{\mu })^{\gamma -1}|\texttt {y}^{\prime }_{\ell {\tau }}(\mathfrak {q})-\texttt {y}_{\ell {\tau }}(\mathfrak {q})|d\mathfrak {q}\\&\precsim |\Psi ^{\prime }_{\mathfrak {j}}(0)-\Psi _{\mathfrak {j}}(0)|+c_{\mathfrak {j}}\frac{\mu ^{1-\gamma }}{\Gamma (\gamma )}\int _{0}^{{\mathfrak {t}}}\mathfrak {q}^{\mu -1}({\mathfrak {t}}^{\mu }-\mathfrak {q}^{\mu })^{\gamma -1}|(\texttt {x}^{\prime }_{\mathfrak {j}}(\mathfrak {q})-\texttt {x}_{\mathfrak {j}}(\mathfrak {q}))|d\mathfrak {q}\\&\quad +{\sum }_{\ell =1}^{n}|\mathfrak {s}_{\mathfrak {j}\ell }^{R}|\theta _{\ell }^{RR}\frac{\mu ^{1-\gamma }}{\Gamma (\gamma )}\int _{0}^{{\mathfrak {t}}}\mathfrak {q}^{\mu -1}({\mathfrak {t}}^{\mu }-\mathfrak {q}^{\mu })^{\gamma -1}|\texttt {x}^{\prime }_{\ell }(\mathfrak {q})-\texttt {x}_{\ell }(\mathfrak {q})|d\mathfrak {q}\\&\quad +{\sum }_{\ell =1}^{n}|\mathfrak {s}_{\mathfrak {j}\ell }^{R}|\theta _{\ell }^{RI}\frac{\mu ^{1-\gamma }}{\Gamma (\gamma )}\int _{0}^{{\mathfrak {t}}}\mathfrak {q}^{\mu -1}({\mathfrak {t}}^{\mu }-\mathfrak {q}^{\mu })^{\gamma -1}|\texttt {y}^{\prime }_{\ell }(\mathfrak {q})-\texttt {y}_{\ell }(\mathfrak {q})|d\mathfrak {q}\\&\quad +{\sum }_{\ell =1}^{n}|\mathfrak {s}_{\mathfrak {j}\ell }^{I}|\theta _{\ell }^{IR}\frac{\mu ^{1-\gamma }}{\Gamma (\gamma )}\int _{0}^{{\mathfrak {t}}}\mathfrak {q}^{\mu -1}({\mathfrak {t}}^{\mu }-\mathfrak {q}^{\mu })^{\gamma -1}|\texttt {x}^{\prime }_{\ell }(\mathfrak {q})-\texttt {x}_{\ell }(\mathfrak {q})|d\mathfrak {q}\\&\quad +{\sum }_{\ell =1}^{n}|\mathfrak {s}_{\mathfrak {j}\ell }^{I}|\theta _{\ell }^{II}\frac{\mu ^{1-\gamma }}{\Gamma (\gamma )}\int _{0}^{{\mathfrak {t}}}\mathfrak {q}^{\mu -1}({\mathfrak {t}}^{\mu }-\mathfrak {q}^{\mu })^{\gamma -1}|\texttt {y}^{\prime }_{\ell }(\mathfrak {q})-\texttt {y}_{\ell }(\mathfrak {q})|d\mathfrak {q}\\&\quad +{\sum }_{\ell =1}^{n}|\mathfrak {p}_{\mathfrak {j}\ell }^{R}|\omega _{\ell }^{RR}\frac{\mu ^{1-\gamma }}{\Gamma (\gamma )}\int _{0}^{{\tau }}\mathfrak {q}^{\mu -1}({\mathfrak {t}}^{\mu }-\mathfrak {q}^{\mu })^{\gamma -1}|\Psi ^{\prime }_{\ell {\tau }}(\mathfrak {q})-\Psi _{\ell {\tau }}(\mathfrak {q})|d\mathfrak {q}\\&\quad +{\sum }_{\ell =1}^{n}|\mathfrak {p}_{\mathfrak {j}\ell }^{R}|\omega _{\ell }^{RR}\frac{\mu ^{1-\gamma }}{\Gamma (\gamma )}\int _{0}^{{\mathfrak {t}}}\mathfrak {q}^{\mu -1}({\mathfrak {t}}^{\mu }-\mathfrak {q}^{\mu })^{\gamma -1}|\texttt {x}^{\prime }_{\ell {\tau }}(\mathfrak {q})-\texttt {x}_{\ell {\tau }}(\mathfrak {q})|d\mathfrak {q} \end{aligned}$$

$$\begin{aligned}&\quad +{\sum }_{\ell =1}^{n}|\mathfrak {p}_{\mathfrak {j}\ell }^{R}|\omega _{\ell }^{RI}\frac{\mu ^{1-\gamma }}{\Gamma (\gamma )}\int _{0}^{{\tau }}\mathfrak {q}^{\mu -1}({\mathfrak {t}}^{\mu }-\mathfrak {q}^{\mu })^{\gamma -1}|\Theta ^{\prime }_{\ell {\tau }}(\mathfrak {q})-\Theta _{\ell {\tau }}(\mathfrak {q})|d\mathfrak {q}\\&\quad +{\sum }_{\ell =1}^{n}|\mathfrak {p}_{\mathfrak {j}\ell }^{R}|\omega _{\ell }^{RI}\frac{\mu ^{1-\gamma }}{\Gamma (\gamma )}\int _{0}^{{\mathfrak {t}}}\mathfrak {q}^{\mu -1}({\mathfrak {t}}^{\mu }-\mathfrak {q}^{\mu })^{\gamma -1}|\texttt {y}^{\prime }_{\ell {\tau }}(\mathfrak {q})-\texttt {y}_{\ell {\tau }}(\mathfrak {q})|d\mathfrak {q}\\&\quad +{\sum }_{\ell =1}^{n}|\mathfrak {p}_{\mathfrak {j}\ell }^{I}|\omega _{\ell }^{IR}\frac{\mu ^{1-\gamma }}{\Gamma (\gamma )}\int _{0}^{{\tau }}\mathfrak {q}^{\mu -1}({\mathfrak {t}}^{\mu }-\mathfrak {q}^{\mu })^{\gamma -1}|\Psi ^{\prime }_{\ell {\tau }}(\mathfrak {q})-\Psi _{\ell {\tau }}(\mathfrak {q})|d\mathfrak {q}\\&\quad +{\sum }_{\ell =1}^{n}|\mathfrak {p}_{\mathfrak {j}\ell }^{I}|\omega _{\ell }^{IR}\frac{\mu ^{1-\gamma }}{\Gamma (\gamma )}\int _{0}^{{\mathfrak {t}}}\mathfrak {q}^{\mu -1}({\mathfrak {t}}^{\mu }-\mathfrak {q}^{\mu })^{\gamma -1}|\texttt {x}^{\prime }_{\ell {\tau }}(\mathfrak {q})-\texttt {x}_{\ell {\tau }}(\mathfrak {q})|d\mathfrak {q}\\&\quad +{\sum }_{\ell =1}^{n}|\mathfrak {p}_{\mathfrak {j}\ell }^{I}|\omega _{\ell }^{II}\frac{\mu ^{1-\gamma }}{\Gamma (\gamma )}\int _{0}^{{\tau }}\mathfrak {q}^{\mu -1}({\mathfrak {t}}^{\mu }-\mathfrak {q}^{\mu })^{\gamma -1}|\Theta ^{\prime }_{\ell {\tau }}(\mathfrak {q})-\Theta _{\ell {\tau }}(\mathfrak {q})|d\mathfrak {q}\\&\quad +{\sum }_{\ell =1}^{n}|\mathfrak {p}_{\mathfrak {j}\ell }^{I}|\omega _{\ell }^{II}\frac{\mu ^{1-\gamma }}{\Gamma (\gamma )}\int _{0}^{{\mathfrak {t}}}\mathfrak {q}^{\mu -1}({\mathfrak {t}}^{\mu }-\mathfrak {q}^{\mu })^{\gamma -1}|\texttt {y}^{\prime }_{\ell {\tau }}(\mathfrak {q})-\texttt {y}_{\ell {\tau }}(\mathfrak {q})|d\mathfrak {q}\\ \end{aligned}$$

Which yields,

$$\begin{aligned}&{e }^{\mathfrak {i}({\mathfrak {t}}-1)}|\texttt 
{x}^{\prime }_{\mathfrak {j}}({\mathfrak {t}})-\texttt {x}_{\mathfrak {j}}({\mathfrak {t}})|\\&\quad \precsim {\sup }_{{\mathfrak {t}}}\{{e }^{\mathfrak {i}({\mathfrak {t}}-1)}|\Psi ^{\prime }_{\mathfrak {j}}({\mathfrak {t}})-\Psi _{\mathfrak {j}}({\mathfrak {t}})|\}+c_{\mathfrak {j}}\frac{\mu ^{1-\gamma }}{\Gamma (\gamma )}{\sup }_{{\mathfrak {t}}}\{{e }^{\mathfrak {i}({\mathfrak {t}}-1)}|(\texttt {x}^{\prime }_{\mathfrak {j}}({\mathfrak {t}})-\texttt {x}_{\mathfrak {j}}({\mathfrak {t}}))|\}\int _{0}^{{\mathfrak {t}}}\kappa ^{\mu -1}({\mathfrak {t}}^{\mu }-\kappa ^{\mu })^{\gamma -1}d\kappa \\&\qquad +[\mathfrak {a}_{1\mathfrak {j}}^{\star }+\mathfrak {a}_{3\mathfrak {j}}^{\star }]{\sum }_{\ell =1}^{n}{\sup }_{{\mathfrak {t}}}\{{e }^{\mathfrak {i}({\mathfrak {t}}-1)}|\texttt {x}^{\prime }_{\ell }({\mathfrak {t}})-\texttt {x}_{\ell }({\mathfrak {t}})|\}\frac{\mu ^{1-\gamma }}{\Gamma (\gamma )}\int _{0}^{{\mathfrak {t}}}\kappa ^{\mu -1}({\mathfrak {t}}^{\mu }-\kappa ^{\mu })^{\gamma -1}d\kappa \\&\qquad +[\mathfrak {a}_{2\mathfrak {j}}^{\star }+\mathfrak {a}_{4\mathfrak {j}}^{\star }]{\sum }_{\ell =1}^{n}{\sup }_{{\mathfrak {t}}}\{{e }^{\mathfrak {i}({\mathfrak {t}}-1)}|\texttt {y}^{\prime }_{\ell }({\mathfrak {t}})-\texttt {y}_{\ell }({\mathfrak {t}})|\}\frac{\mu ^{1-\gamma }}{\Gamma (\gamma )}\int _{0}^{{\mathfrak {t}}}\kappa ^{\mu -1}({\mathfrak {t}}^{\mu }-\kappa ^{\mu })^{\gamma -1}d\kappa \\&\qquad +[\mathfrak {b}_{1\mathfrak {j}}^{\star }+\mathfrak {b}_{3\mathfrak {j}}^{\star }]{\sum }_{\ell =1}^{n}{\sup }_{{\mathfrak {t}}}\{{e }^{\mathfrak {i}(\wp -1)}|\Psi ^{\prime }_{\ell }(\wp )-\Psi _{\ell }(\wp )|\}\frac{\mu ^{1-\gamma }}{\Gamma (\gamma )}\int _{0}^{{\tau }}\wp _{1}^{\mu -1}({\mathfrak {t}}^{\mu }-\wp _{1}^{\mu })^{\gamma -1}d\wp _{1}\\&\qquad +[\mathfrak {b}_{1\mathfrak {j}}^{\star }+\mathfrak {b}_{3\mathfrak {j}}^{\star }]{\sum }_{\ell =1}^{n}{\sup }_{{\mathfrak {t}}}\{{e }^{\mathfrak {i}(\wp -1)}|\texttt {x}^{\prime }_{\ell }(\wp )-\texttt {x}_{\ell }(\wp )|\}\frac{\mu ^{1-\gamma }}{\Gamma (\gamma )}\int _{{\tau }}^{{\mathfrak {t}}}\wp _{1}^{\mu -1}({\mathfrak {t}}^{\mu }-\wp _{1}^{\mu })^{\gamma -1}d\wp _{1}\\&\qquad +[\mathfrak {b}_{2\mathfrak {j}}^{\star }+\mathfrak {b}_{4\mathfrak {j}}^{\star }]{\sum }_{\ell =1}^{n}{\sup }_{{\mathfrak {t}}}\{{e }^{\mathfrak {i}(\wp -1)}|\Theta ^{\prime }_{\ell }(\wp )-\Theta _{\ell }(\wp )|\}\frac{\mu ^{1-\gamma }}{\Gamma (\gamma )}\int _{0}^{{\tau }}\wp _{1}^{\mu -1}({\mathfrak {t}}^{\mu }-\wp _{1}^{\mu })^{\gamma -1}d\wp _{1}\\&\qquad +[\mathfrak {b}_{2\mathfrak {j}}^{\star }+\mathfrak {b}_{4\mathfrak {j}}^{\star }]{\sum }_{\ell =1}^{n}{\sup }_{{\mathfrak {t}}}\{{e }^{\mathfrak {i}(\wp -1)}|\texttt {y}^{\prime }_{\ell }(\wp )-\texttt {y}_{\ell }(\wp )|\}\frac{\mu ^{1-\gamma }}{\Gamma (\gamma )}\int _{{\tau }}^{{\mathfrak {t}}}\wp _{1}^{\mu -1}({\mathfrak {t}}^{\mu }-\wp _{1}^{\mu })^{\gamma -1}d\wp _{1}\\&\quad \precsim {\sup }_{{\mathfrak {t}}}\{{e }^{\mathfrak {i}({\mathfrak {t}}-1)}|\Psi ^{\prime }_{\mathfrak {j}}({\mathfrak {t}})-\Psi _{\mathfrak {j}}({\mathfrak {t}})|\}+c_{\mathfrak {j}}\frac{\mu ^{1-\gamma }}{\Gamma (\gamma )}{\sup }_{{\mathfrak {t}}}\{{e }^{\mathfrak {i}({\mathfrak {t}}-1)}|(\texttt {x}^{\prime }_{\mathfrak {j}}({\mathfrak {t}})-\texttt {x}_{\mathfrak {j}}({\mathfrak {t}}))|\}\int _{0}^{{\mathfrak {t}}}\kappa ^{\mu -1}({\mathfrak {t}}^{\mu }-\kappa ^{\mu })^{\gamma -1}d\kappa \\&\qquad +[\mathfrak {a}_{1\mathfrak {j}}^{\star }+\mathfrak {a}_{3\mathfrak {j}}^{\star }]{\sum }_{\ell =1}^{n}{\sup }_{{\mathfrak {t}}}\{{e }^{\mathfrak {i}({\mathfrak {t}}-1)}|\texttt {x}^{\prime }_{\ell }({\mathfrak {t}})-\texttt {x}_{\ell }({\mathfrak {t}})|\}\frac{\mu ^{1-\gamma }}{\Gamma (\gamma )}\int _{0}^{{\mathfrak {t}}}\kappa ^{\mu -1}({\mathfrak {t}}^{\mu }-\kappa ^{\mu })^{\gamma -1}d\kappa \end{aligned}$$

$$\begin{aligned}&\qquad +[\mathfrak {a}_{2\mathfrak {j}}^{\star }+\mathfrak {a}_{4\mathfrak {j}}^{\star }]{\sum }_{\ell =1}^{n}{\sup }_{{\mathfrak {t}}}\{{e }^{\mathfrak {i}({\mathfrak {t}}-1)}|\texttt {y}^{\prime }_{\ell }({\mathfrak {t}})-\texttt {y}_{\ell }({\mathfrak {t}})|\}\frac{\mu ^{1-\gamma }}{\Gamma (\gamma )}\int _{0}^{{\mathfrak {t}}}\kappa ^{\mu -1}({\mathfrak {t}}^{\mu }-\kappa ^{\mu })^{\gamma -1}d\kappa \\&\qquad +[\mathfrak {b}_{1\mathfrak {j}}^{\star }+\mathfrak {b}_{3\mathfrak {j}}^{\star }]{\sum }_{\ell =1}^{n}{\sup }_{{\mathfrak {t}}}\{{e }^{\mathfrak {i}({\mathfrak {t}}-1)}|\Psi ^{\prime }_{\ell }(\wp )-\Psi _{\ell }(\wp )|\}\frac{\mu ^{1-\gamma }}{\Gamma (\gamma )}\int _{0}^{{\tau }}\wp ^{\mu -1}({\mathfrak {t}}^{\mu }-\wp ^{\mu })^{\gamma -1}d\wp \\&\qquad +[\mathfrak {b}_{1\mathfrak {j}}^{\star }+\mathfrak {b}_{3\mathfrak {j}}^{\star }]{\sum }_{\ell =1}^{n}{\sup }_{{\mathfrak {t}}}\{{e }^{\mathfrak {i}({\mathfrak {t}}-1)}|\texttt {x}^{\prime }_{\ell }(\wp )-\texttt {x}_{\ell }(\wp )|\}\frac{\mu ^{1-\gamma }}{\Gamma (\gamma )}\int _{{\tau }}^{{\mathfrak {t}}}\wp ^{\mu -1}({\mathfrak {t}}^{\mu }-\wp ^{\mu })^{\gamma -1}d\wp \\&\qquad +[\mathfrak {b}_{2\mathfrak {j}}^{\star }+\mathfrak {b}_{4\mathfrak {j}}^{\star }]{\sum }_{\ell =1}^{n}{\sup }_{{\mathfrak {t}}}\{{e }^{\mathfrak {i}({\mathfrak {t}}-1)}|\Theta ^{\prime }_{\ell }(\wp )-\Theta _{\ell }(\wp )|\}\frac{\mu ^{1-\gamma }}{\Gamma (\gamma )}\int _{0}^{{\tau }}\wp ^{\mu -1}({\mathfrak {t}}^{\mu }-\wp ^{\mu })^{\gamma -1}d\wp \\&\qquad +[\mathfrak {b}_{2\mathfrak {j}}^{\star }+\mathfrak {b}_{4\mathfrak {j}}^{\star }]{\sum }_{\ell =1}^{n}{\sup }_{{\mathfrak {t}}}\{{e }^{\mathfrak {i}({\mathfrak {t}}-1)}|\texttt {y}^{\prime }_{\ell }(\wp )-\texttt {y}_{\ell }(\wp )|\}\frac{\mu ^{1-\gamma }}{\Gamma (\gamma )}\int _{{\tau }}^{{\mathfrak {t}}}\wp ^{\mu -1}({\mathfrak {t}}^{\mu }-\wp ^{\mu })^{\gamma -1}d\wp \end{aligned}$$

$$\begin{aligned}&\quad \precsim {\sup }_{{\mathfrak {t}}}\{{e }^{\mathfrak {i}({\mathfrak {t}}-1)}|\Psi ^{\prime }_{\mathfrak {j}}({\mathfrak {t}})-\Psi _{\mathfrak {j}}({\mathfrak {t}})|\}+c_{\mathfrak {j}}\frac{\mu ^{1-\gamma }}{\Gamma (\gamma )}{\sup }_{{\mathfrak {t}}}\{{e }^{\mathfrak {i}({\mathfrak {t}}-1)}|(\texttt {x}^{\prime }_{\mathfrak {j}}({\mathfrak {t}})-\texttt {x}_{\mathfrak {j}}({\mathfrak {t}}))|\}\int _{0}^{{\mathfrak {t}}}\kappa ^{\mu -1}({\mathfrak {t}}^{\mu }-\kappa ^{\mu })^{\gamma -1}d\kappa \\&\qquad +[\mathfrak {a}_{1\mathfrak {j}}^{\star }+\mathfrak {a}_{3\mathfrak {j}}^{\star }]{\sum }_{\ell =1}^{n}{\sup }_{{\mathfrak {t}}}\{{e }^{\mathfrak {i}({\mathfrak {t}}-1)}|\texttt {x}^{\prime }_{\ell }({\mathfrak {t}})-\texttt {x}_{\ell }({\mathfrak {t}})|\}\frac{\mu ^{1-\gamma }}{\Gamma (\gamma )}\int _{0}^{{\mathfrak {t}}}\kappa ^{\mu -1}({\mathfrak {t}}^{\mu }-\kappa ^{\mu })^{\gamma -1}d\kappa \\&\qquad +[\mathfrak {a}_{2\mathfrak {j}}^{\star }+\mathfrak {a}_{4\mathfrak {j}}^{\star }]{\sum }_{\ell =1}^{n}{\sup }_{{\mathfrak {t}}}\{{e }^{\mathfrak {i}({\mathfrak {t}}-1)}|\texttt {y}^{\prime }_{\ell }({\mathfrak {t}})-\texttt {y}_{\ell }({\mathfrak {t}})|\}\frac{\mu ^{1-\gamma }}{\Gamma (\gamma )}\int _{0}^{{\mathfrak {t}}}\kappa ^{\mu -1}({\mathfrak {t}}^{\mu }-\kappa ^{\mu })^{\gamma -1}d\kappa \\&\qquad +[\mathfrak {b}_{1\mathfrak {j}}^{\star }+\mathfrak {b}_{3\mathfrak {j}}^{\star }]\frac{\mu ^{1-\gamma }}{\Gamma (\gamma )}{\sum }_{\ell =1}^{n}\int _{-{\tau }}^{0}(\mathfrak {q}+{\tau })^{\mu -1}[{\mathfrak {t}}^{\mu }-(\mathfrak {q}+{\tau })^{\mu }]^{\gamma -1}{e }^{\mathfrak {i}(\mathfrak {q}-1)}|\Psi ^{\prime }_{\ell }(\mathfrak {q})-\Psi _{\ell }(\mathfrak {q})|d\mathfrak {q}\\&\qquad +[\mathfrak {b}_{1\mathfrak {j}}^{\star }+\mathfrak {b}_{3\mathfrak {j}}^{\star }]\frac{\mu ^{1-\gamma }}{\Gamma (\gamma )}{\sum }_{\ell =1}^{n}\int _{0}^{{\mathfrak {t}}-{\tau }}(\mathfrak {q}+{\tau })^{\mu -1}[{\mathfrak {t}}^{\mu }-(\mathfrak {q}+{\tau })^{\mu }]^{\gamma -1}{e }^{\mathfrak {i}(\mathfrak {q}-1)}|\texttt {x}^{\prime }_{\ell }(\mathfrak {q})-\texttt {x}_{\ell }(\mathfrak {q})|d\mathfrak {q}\\&\qquad +[\mathfrak {b}_{2\mathfrak {j}}^{\star }+\mathfrak {b}_{4\mathfrak {j}}^{\star }]\frac{\mu ^{1-\gamma }}{\Gamma (\gamma )}{\sum }_{\ell =1}^{n}\int _{-{\tau }}^{0}(\mathfrak {q}+{\tau })^{\mu -1}[{\mathfrak {t}}^{\mu }-(\mathfrak {q}+{\tau })^{\mu }]^{\gamma -1}{e }^{\mathfrak {i}(\mathfrak {q}-1)}|\Theta ^{\prime }_{\ell }(\mathfrak {q})-\Theta _{\ell }(\mathfrak {q})|d\mathfrak {q}\\&\qquad +[\mathfrak {b}_{2\mathfrak {j}}^{\star }+\mathfrak {b}_{4\mathfrak {j}}^{\star }]\frac{\mu ^{1-\gamma }}{\Gamma (\gamma )}{\sum }_{\ell =1}^{n}\int _{0}^{{\mathfrak {t}}-{\tau }}(\mathfrak {q}+{\tau })^{\mu -1}[{\mathfrak {t}}^{\mu }-(\mathfrak {q}+{\tau })^{\mu }]^{\gamma -1}{e }^{\mathfrak {i}(\mathfrak {q}-1)}|\texttt {y}^{\prime }_{\ell }(\mathfrak {q})-\texttt {y}_{\ell }(\mathfrak {q})|d\mathfrak {q} \end{aligned}$$

$$\begin{aligned}&\quad \precsim {\sup }_{{\mathfrak {t}}}\{{e }^{\mathfrak {i}({\mathfrak {t}}-1)}|\Psi ^{\prime }_{\mathfrak {j}}({\mathfrak {t}})-\Psi _{\mathfrak {j}}({\mathfrak {t}})|\}+c_{\mathfrak {j}}\frac{\mu ^{1-\gamma }}{\Gamma (\gamma )}{\sup }_{{\mathfrak {t}}}\{{e }^{\mathfrak {i}({\mathfrak {t}}-1)}|(\texttt {x}^{\prime }_{\mathfrak {j}}({\mathfrak {t}})-\texttt {x}_{\mathfrak {j}}({\mathfrak {t}}))|\}\int _{0}^{{\mathfrak {t}}}\kappa ^{\mu -1}({\mathfrak {t}}^{\mu }-\kappa ^{\mu })^{\gamma -1}d\kappa \\&\qquad +[\mathfrak {a}_{1\mathfrak {j}}^{\star }+\mathfrak {a}_{3\mathfrak {j}}^{\star }]{\sum }_{\ell =1}^{n}{\sup }_{{\mathfrak {t}}}\{{e }^{\mathfrak {i}({\mathfrak {t}}-1)}|\texttt {x}^{\prime }_{\ell }({\mathfrak {t}})-\texttt {x}_{\ell }({\mathfrak {t}})|\}\frac{\mu ^{1-\gamma }}{\Gamma (\gamma )}\int _{0}^{{\mathfrak {t}}}\kappa ^{\mu -1}({\mathfrak {t}}^{\mu }-\kappa ^{\mu })^{\gamma -1}d\kappa \\&\qquad +[\mathfrak {a}_{2\mathfrak {j}}^{\star }+\mathfrak {a}_{4\mathfrak {j}}^{\star }]{\sum }_{\ell =1}^{n}{\sup }_{{\mathfrak {t}}}\{{e }^{\mathfrak {i}({\mathfrak {t}}-1)}|\texttt {y}^{\prime }_{\ell }({\mathfrak {t}})-\texttt {y}_{\ell }({\mathfrak {t}})|\}\frac{\mu 
^{1-\gamma }}{\Gamma (\gamma )}\int _{0}^{{\mathfrak {t}}}\kappa ^{\mu -1}({\mathfrak {t}}^{\mu }-\kappa ^{\mu })^{\gamma -1}d\kappa \\&\qquad +[\mathfrak {b}_{1\mathfrak {j}}^{\star }+\mathfrak {b}_{3\mathfrak {j}}^{\star }]{\sum }_{\ell =1}^{n}{e }^{\mathfrak {i}({\mathfrak {t}}-1)}|\Psi ^{\prime }_{\ell }({\mathfrak {t}})-\Psi _{\ell }({\mathfrak {t}})|\frac{\mu ^{1-\gamma }}{\mu \Gamma (\gamma )}\int _{{\mathfrak {t}}^{\mu }-{\tau }^{\mu }}^{{\mathfrak {t}}^{\mu }}{z}^{\gamma -1}d{z}\\&\qquad +[\mathfrak {b}_{1\mathfrak {j}}^{\star }+\mathfrak {b}_{3\mathfrak {j}}^{\star }]{\sum }_{\ell =1}^{n}{e }^{\mathfrak {i}({\mathfrak {t}}-1)}|\texttt {x}^{\prime }_{\ell }({\mathfrak {t}})-\texttt {x}_{\ell }({\mathfrak {t}})|\frac{\mu ^{1-\gamma }}{\mu \Gamma (\gamma )}\int _{0}^{{\mathfrak {t}}^{\mu }-{\tau }^{\mu }}{z}^{\gamma -1}d{z}\\&\qquad +[\mathfrak {b}_{2\mathfrak {j}}^{\star }+\mathfrak {b}_{4\mathfrak {j}}^{\star }]\frac{\mu ^{1-\gamma }}{\mu \Gamma (\gamma )}{\sum }_{\ell =1}^{n}{e }^{\mathfrak {i}({\mathfrak {t}}-1)}|\Theta ^{\prime }_{\ell }({\mathfrak {t}})-\Theta _{\ell }({\mathfrak {t}})|\int _{{\mathfrak {t}}^{\mu }-{\tau }^{\mu }}^{{\mathfrak {t}}^{\mu }}{z}^{\gamma -1}d{z}\\&\qquad +[\mathfrak {b}_{2\mathfrak {j}}^{\star }+\mathfrak {b}_{4\mathfrak {j}}^{\star }]\frac{\mu ^{1-\gamma }}{\mu \Gamma (\gamma )}{\sum }_{\ell =1}^{n}{e }^{\mathfrak {i}({\mathfrak {t}}-1)}|\texttt {y}^{\prime }_{\ell }({\mathfrak {t}})-\texttt {y}_{\ell }({\mathfrak {t}})|\int _{0}^{{\mathfrak {t}}^{\mu }-{\tau }^{\mu }}{z}^{\gamma -1}d{z} \end{aligned}$$

$$\begin{aligned}&\precsim {\sup }_{{\mathfrak {t}}}\{{e }^{\mathfrak {i}({\mathfrak {t}}-1)}|\Psi ^{\prime }_{\mathfrak {j}}({\mathfrak {t}})-\Psi _{\mathfrak {j}}({\mathfrak {t}})|\}+\frac{\mu ^{1-\gamma }}{\Gamma (\gamma )}\Biggl [c_{\mathfrak {j}}{\sup }_{{\mathfrak {t}}}\{{e }^{\mathfrak {i}({\mathfrak {t}}-1)}|(\texttt {x}^{\prime }_{\mathfrak {j}}({\mathfrak {t}})-\texttt {x}_{\mathfrak {j}}({\mathfrak {t}}))|\}\frac{{\mathfrak {t}}^{\gamma \mu }}{\gamma \mu }\\&\qquad +[\mathfrak {a}_{1\mathfrak {j}}^{\star }+\mathfrak {a}_{3\mathfrak {j}}^{\star }]{\sum }_{\ell =1}^{n}{\sup }_{{\mathfrak {t}}}\{{e }^{\mathfrak {i}({\mathfrak {t}}-1)}|\texttt {x}^{\prime }_{\ell }({\mathfrak {t}})-\texttt {x}_{\ell }({\mathfrak {t}})|\}\frac{{\mathfrak {t}}^{\gamma \mu }}{\gamma \mu }\\&\qquad +[\mathfrak {a}_{2\mathfrak {j}}^{\star }+\mathfrak {a}_{4\mathfrak {j}}^{\star }]{\sum }_{\ell =1}^{n}{\sup }_{{\mathfrak {t}}}\{{e }^{\mathfrak {i}({\mathfrak {t}}-1)}|\texttt {y}^{\prime }_{\ell }({\mathfrak {t}})-\texttt {y}_{\ell }({\mathfrak {t}})|\}\frac{{\mathfrak {t}}^{\gamma \mu }}{\gamma \mu }\\&\qquad +[\mathfrak {b}_{1\mathfrak {j}}^{\star }+\mathfrak {b}_{3\mathfrak {j}}^{\star }]{\sum }_{\ell =1}^{n}{\sup }_{{\mathfrak {t}}}\{{e }^{\mathfrak {i}({\mathfrak {t}}-1)}|\Psi ^{\prime }_{\ell }({\mathfrak {t}})-\Psi _{\ell }({\mathfrak {t}})|\}\biggl (\frac{{\mathfrak {t}}^{\gamma \mu }}{\gamma \mu }-\frac{({\mathfrak {t}}^{\mu }-{\tau }^{\mu })^{\gamma }}{\gamma \mu }\biggr )\\&\qquad +[\mathfrak {b}_{1\mathfrak {j}}^{\star }+\mathfrak {b}_{3\mathfrak {j}}^{\star }]{\sum }_{\ell =1}^{n}{\sup }_{{\mathfrak {t}}}\{{e }^{\mathfrak {i}({\mathfrak {t}}-1)}|\texttt {x}^{\prime }_{\ell }({\mathfrak {t}})-\texttt {x}_{\ell }({\mathfrak {t}})|\}\biggl (\frac{({\mathfrak {t}}^{\mu }-{\tau }^{\mu })^{\gamma }}{\gamma \mu }\biggr )\\&\qquad +[\mathfrak {b}_{2\mathfrak {j}}^{\star }+\mathfrak {b}_{4\mathfrak {j}}^{\star }]{\sum }_{\ell =1}^{n}{\sup }_{{\mathfrak {t}}}\{{e }^{\mathfrak {i}({\mathfrak {t}}-1)}|\Theta ^{\prime }_{\ell }({\mathfrak {t}})-\Theta _{\ell }({\mathfrak {t}})|\}\biggl (\frac{{\mathfrak {t}}^{\gamma \mu }}{\gamma \mu }-\frac{({\mathfrak {t}}^{\mu }-{\tau }^{\mu })^{\gamma }}{\gamma \mu }\biggr )\\&\qquad +[\mathfrak {b}_{2\mathfrak {j}}^{\star }+\mathfrak {b}_{4\mathfrak {j}}^{\star }]{\sum }_{\ell =1}^{n}{\sup }_{{\mathfrak {t}}}\{{e }^{\mathfrak {i}({\mathfrak {t}}-1)}|\texttt {y}^{\prime }_{\ell }({\mathfrak {t}})-\texttt {y}_{\ell }({\mathfrak {t}})|\}\biggl (\frac{({\mathfrak {t}}^{\mu }-{\tau }^{\mu })^{\gamma }}{\gamma \mu }\biggr )\Biggr ]\\&\precsim {\sup }_{{\mathfrak {t}}}\{{e }^{\mathfrak {i}({\mathfrak {t}}-1)}|\Psi ^{\prime }_{\mathfrak {j}}({\mathfrak {t}})-\Psi _{\mathfrak {j}}({\mathfrak {t}})|\}+\frac{{\mathfrak {t}}^{\gamma \mu }}{\mu ^{\gamma }\Gamma (\gamma +1)}c_{\mathfrak {j}}{\sup }_{{\mathfrak {t}}}\{{e }^{\mathfrak {i}({\mathfrak {t}}-1)}|(\texttt {x}^{\prime }_{\mathfrak {j}}({\mathfrak {t}})-\texttt {x}_{\mathfrak {j}}({\mathfrak {t}}))|\} \end{aligned}$$

$$\begin{aligned}&\qquad +\frac{{\mathfrak {t}}^{\gamma \mu }}{\mu ^{\gamma }\Gamma (\gamma +1)}[\mathfrak {a}_{1\mathfrak {j}}^{\star }+\mathfrak {a}_{3\mathfrak {j}}^{\star }]||\texttt {x}^{\prime }_{\ell }({\mathfrak {t}})-\texttt {x}_{\ell }({\mathfrak {t}})|| +\frac{{\mathfrak {t}}^{\gamma \mu }}{\mu ^{\gamma }\Gamma (\gamma +1)}[\mathfrak {a}_{2\mathfrak {j}}^{\star }+\mathfrak {a}_{4\mathfrak {j}}^{\star }]||\texttt {y}^{\prime }({\mathfrak {t}})-\texttt {y}({\mathfrak {t}})||\\&\qquad +\frac{{\mathfrak {t}}^{\gamma \mu }}{\mu ^{\gamma }\Gamma (\gamma +1)}[\mathfrak {b}_{1\mathfrak {j}}^{\star }+\mathfrak {b}_{3\mathfrak {j}}^{\star }]||\Psi ^{\prime }({\mathfrak {t}})-\Psi ({\mathfrak {t}})|| +\frac{({\mathfrak {t}}^{\mu }-{\tau }^{\mu })^{\gamma }}{\mu ^{\gamma }\Gamma (\gamma +1)}[\mathfrak {b}_{1\mathfrak {j}}^{\star }+\mathfrak {b}_{3\mathfrak {j}}^{\star }]||\Psi ^{\prime }({\mathfrak {t}})-\Psi ({\mathfrak {t}})||\\&\qquad +\frac{({\mathfrak {t}}^{\mu }-{\tau }^{\mu })^{\gamma }}{\mu ^{\gamma }\Gamma (\gamma +1)}[\mathfrak {b}_{1\mathfrak {j}}^{\star }+\mathfrak {b}_{3\mathfrak {j}}^{\star }]||\texttt {x}^{\prime }({\mathfrak {t}})-\texttt {x}({\mathfrak {t}})|| +\frac{{\mathfrak {t}}^{\gamma \mu }}{\mu ^{\gamma }\Gamma (\gamma +1)}[\mathfrak {b}_{2\mathfrak {j}}^{\star }+\mathfrak {b}_{4\mathfrak {j}}^{\star }]||\Theta ^{\prime }({\mathfrak {t}})-\Theta ({\mathfrak {t}})||\\&\qquad +\frac{({\mathfrak {t}}^{\mu }-{\tau }^{\mu })^{\gamma }}{\mu ^{\gamma }\Gamma (\gamma +1)}[\mathfrak {b}_{2\mathfrak {j}}^{\star }+\mathfrak {b}_{4\mathfrak {j}}^{\star }]||\Theta ^{\prime }({\mathfrak {t}})-\Theta ({\mathfrak {t}})|| +\frac{({\mathfrak {t}}^{\mu }-{\tau }^{\mu })^{\gamma }}{\mu ^{\gamma }\Gamma (\gamma +1)}[\mathfrak {b}_{2\mathfrak {j}}^{\star }+\mathfrak {b}_{4\mathfrak {j}}^{\star }]||\texttt {y}^{\prime }({\mathfrak {t}})-\texttt {y}({\mathfrak {t}})||\\ \end{aligned}$$

Using the equation aforementioned, it is simple to determine that

2.8 $$\begin{aligned} ||\texttt {x}^{\prime }({\mathfrak {t}})-\texttt {x}({\mathfrak {t}})||&= {\sum }_{\mathfrak {j}=1}^{n}{\sup }_{{\mathfrak {t}}}\{{e }^{\mathfrak {i}({\mathfrak {t}}-1)}|\texttt {x}^{\prime }_{\mathfrak {j}}({\mathfrak {t}})-\texttt {x}_{\mathfrak {j}}({\mathfrak {t}})|\}\nonumber \\&\precsim \bigl [c_{\max }+||\mathfrak {a}_{1}^{\star }+\mathfrak {a}_{3}^{\star }||+||\mathfrak {b}_{1}^{\star }+\mathfrak {b}_{3}^{\star }||\bigr ]||\texttt {x}^{\prime }({\mathfrak {t}})-\texttt {x}({\mathfrak {t}})||\nonumber \\&\quad +\bigl [||\mathfrak {a}_{2}^{\star }+\mathfrak {a}_{4}^{\star }||+||\mathfrak {b}_{2}^{\star }+\mathfrak {b}_{4}^{\star }||\bigr ]||\texttt {y}^{\prime }({\mathfrak {t}})-\texttt {y}({\mathfrak {t}})||\nonumber \\&\quad +\bigl [1+2||\mathfrak {b}_{1}^{\star }+\mathfrak {b}_{3}^{\star }||\bigr ]||\Psi ^{\prime }({\mathfrak {t}})-\Psi ({\mathfrak {t}})||+\bigl [2||\mathfrak {b}_{2}^{\star }+\mathfrak {b}_{4}^{\star }||\bigr ]||\Theta ^{\prime }({\mathfrak {t}})-\Theta ({\mathfrak {t}})||\nonumber \\ ||\texttt {x}^{\prime }({\mathfrak {t}})-\texttt {x}({\mathfrak {t}})||&\precsim \frac{||\mathfrak {a}_{2}^{\star }+\mathfrak {a}_{4}^{\star }||+||\mathfrak {b}_{2}^{\star }+\mathfrak {b}_{4}^{\star }||}{1-\Big [c_{\max }+||\mathfrak {a}_{1}^{\star }+\mathfrak {a}_{3}^{\star }||+||\mathfrak {b}_{1}^{\star }+\mathfrak {b}_{3}^{\star }||\Big ]}||\texttt {y}^{\prime }({\mathfrak {t}})-\texttt {y}({\mathfrak {t}})||\nonumber \\&\quad +\frac{1+2||\mathfrak {b}_{1}^{\star }+\mathfrak {b}_{3}^{\star }||}{1-\Big [c_{\max }+||\mathfrak {a}_{1}^{\star }+\mathfrak {a}_{3}^{\star }||+||\mathfrak {b}_{1}^{\star }+\mathfrak {b}_{3}^{\star }||\Big ]}||\Psi ^{\prime }({\mathfrak {t}})-\Psi ({\mathfrak {t}})||\nonumber \\&\quad +\frac{2||\mathfrak {b}_{2}^{\star }+\mathfrak {b}_{4}^{\star }||}{1-\Big [c_{\max }+||\mathfrak {a}_{1}^{\star }+\mathfrak {a}_{3}^{\star }||+||\mathfrak {b}_{1}^{\star }+\mathfrak {b}_{3}^{\star }||\Big ]}||\Theta ^{\prime }({\mathfrak {t}})-\Theta ({\mathfrak {t}})||\nonumber \\&\precsim \biggl (\frac{1}{1-1-\Big [c_{\max }+||\mathfrak {a}_{1}^{\star }+\mathfrak {a}_{3}^{\star }||+||\mathfrak {b}_{1}^{\star }+\mathfrak {b}_{3}^{\star }||\Big ]}\biggr )\biggl (\Big [||\mathfrak {a}_{2}^{\star }+\mathfrak {a}_{4}^{\star }||+||\mathfrak {b}_{2}^{\star }+\mathfrak {b}_{4}^{\star }||\Big ]||\texttt {y}^{\prime }({\mathfrak {t}})-\texttt {y}({\mathfrak {t}})||\nonumber \\&\quad \Big [1+2||\mathfrak {b}_{1}^{\star }+\mathfrak {b}_{3}^{\star }||\Big ]||\Psi ^{\prime }({\mathfrak {t}})-\Psi ({\mathfrak {t}})||+ \Big [2||\mathfrak {b}_{2}^{\star }+\mathfrak {b}_{4}^{\star }||\Big ]||\Theta ^{\prime }({\mathfrak {t}})-\Theta ({\mathfrak {t}})||\biggr ) \end{aligned}$$

Now consider,

$$\begin{aligned} ^{C }\mathscr {D}_{{\mathfrak {t}}_{0}}^{\gamma ,\mu }{} \texttt {y}_{\mathfrak {j}}({\mathfrak {t}})&=-c_{\mathfrak {j}}{} \texttt {y}_{\mathfrak {j}}({\mathfrak {t}})+{\sum }_{\ell =1}^{n}\mathfrak {s}_{\mathfrak {j}\ell }^{I}\varphi _{\ell }^{R}(\texttt {x}_{\ell },\texttt {y}_{\ell })+{\sum }_{\ell =1}^{n}\mathfrak {s}_{\mathfrak {j}\ell }^{R}\varphi _{\ell }^{I}(\texttt {x}_{\ell },\texttt {y}_{\ell })\nonumber \\&\quad +{\sum }_{\ell =1}^{n}\mathfrak {p}_{\mathfrak 
{j}\ell }^{I}\varphi _{\ell }^{R}(\texttt {x}_{\ell {\tau }},\texttt {y}_{\ell {\tau }})+{\sum }_{\ell =1}^{n}\mathfrak {p}_{\mathfrak {j}\ell }^{R}\varphi _{\ell }^{I}(\texttt {x}_{\ell {\tau }},\texttt {y}_{\ell {\tau }})+\Upsilon _{\mathfrak {j}}^{I} \end{aligned}$$

which implies

2.9 $$\begin{aligned}&^{C }\mathscr {D}_{{\mathfrak {t}}_{0}}^{\gamma ,\mu }(\texttt {y}^{\prime }_{\mathfrak {j}}({\mathfrak {t}})-\texttt {y}_{\mathfrak {j}}({\mathfrak {t}}))\nonumber \\&=-c_{\mathfrak {j}}(\texttt {y}^{\prime }_{\mathfrak {j}}({\mathfrak {t}})-\texttt {y}_{\mathfrak {j}}({\mathfrak {t}}))+{\sum }_{\ell =1}^{n}\mathfrak {s}_{\mathfrak {j}\ell }^{I}\bigg [\varphi _{\ell }^{R}(\texttt {x}_{\ell }^{\prime },\texttt {y}_{\ell }^{\prime })-\varphi _{\ell }^{R}(\texttt {x}_{\ell },\texttt {y}_{\ell })\bigg ]-{\sum }_{\ell =1}^{n}\mathfrak {s}_{\mathfrak {j}\ell }^{R}\bigg [\varphi _{\ell }^{I}(\texttt {x}_{\ell }^{\prime },\texttt {y}_{\ell }^{\prime })-\varphi _{\ell }^{I}(\texttt {x}_{\ell },\texttt {y}_{\ell })\bigg ]\nonumber \\&\quad +{\sum }_{\ell =1}^{n}\mathfrak {p}_{\mathfrak {j}\ell }^{I}\bigg [\varphi _{\ell }^{R}(\texttt {x}_{\ell {\tau }}^{\prime },\texttt {y}_{\ell {\tau }}^{\prime })-\varphi _{\ell }^{R}(\texttt {x}_{\ell {\tau }},\texttt {y}_{\ell {\tau }})\bigg ]-{\sum }_{\ell =1}^{n}\mathfrak {p}_{\mathfrak {j}\ell }^{R}\bigg [\varphi _{\ell }^{I}(\texttt {x}_{\ell {\tau }}^{\prime },\texttt {y}_{\ell {\tau }}^{\prime })-\varphi _{\ell }^{I}(\texttt {x}_{\ell {\tau }},\texttt {y}_{\ell {\tau }})\bigg ] \end{aligned}$$

The integral equation underneath is equal to Eq. (2.9) :

$$\begin{aligned}&\texttt {y}^{\prime }_{\mathfrak {j}}({\mathfrak {t}})-\texttt {y}_{\mathfrak {j}}({\mathfrak {t}})\\&=\Theta ^{\prime }_{\mathfrak {j}}(0)-\Theta _{\mathfrak {j}}(0)+\frac{\mu ^{1-\gamma }}{\Gamma (\gamma )}\int _{0}^{{\mathfrak {t}}}\mathfrak {q}^{\mu -1}({\mathfrak {t}}^{\mu }-\mathfrak {q}^{\mu })^{\gamma -1}\\&\quad \times \Biggl (-c_{\mathfrak {j}}(\texttt {y}^{\prime }_{\mathfrak {j}}(\mathfrak {q})-\texttt {y}_{\mathfrak {j}}(\mathfrak {q}))+{\sum }_{\ell =1}^{n}\mathfrak {s}_{\mathfrak {j}\ell }^{I}\bigg [\varphi _{\ell }^{R}(\texttt {x}_{\ell }^{\prime },\texttt {y}_{\ell }^{\prime })-\varphi _{\ell }^{R}(\texttt {x}_{\ell },\texttt {y}_{\ell })\bigg ]-{\sum }_{\ell =1}^{n}\mathfrak {s}_{\mathfrak {j}\ell }^{R}\bigg [\varphi _{\ell }^{I}(\texttt {x}_{\ell }^{\prime },\texttt {y}_{\ell }^{\prime })-\varphi _{\ell }^{I}(\texttt {x}_{\ell },\texttt {y}_{\ell })\bigg ]\\&\quad +{\sum }_{\ell =1}^{n}\mathfrak {p}_{\mathfrak {j}\ell }^{I}\bigg [\varphi _{\ell }^{R}(\texttt {x}_{\ell {\tau }}^{\prime },\texttt {y}_{\ell {\tau }}^{\prime })-\varphi _{\ell }^{R}(\texttt {x}_{\ell {\tau }},\texttt {y}_{\ell {\tau }})\bigg ]-{\sum }_{\ell =1}^{n}\mathfrak {p}_{\mathfrak {j}\ell }^{R}\bigg [\varphi _{\ell }^{I}(\texttt {x}_{\ell {\tau }}^{\prime },\texttt {y}_{\ell {\tau }}^{\prime })-\varphi _{\ell }^{I}(\texttt {x}_{\ell {\tau }},\texttt {y}_{\ell {\tau }})\bigg ]\Biggr )d\mathfrak {q} \end{aligned}$$

$$\begin{aligned}&\texttt {y}^{\prime }_{\mathfrak {j}}({\mathfrak {t}})-\texttt {y}_{\mathfrak {j}}({\mathfrak {t}})\\&\quad \precsim |\Theta ^{\prime }_{\mathfrak {j}}(0)-\Theta _{\mathfrak {j}}(0)|+\frac{\mu ^{1-\gamma }}{\Gamma (\gamma )}\int _{0}^{{\mathfrak {t}}}\mathfrak {q}^{\mu -1}({\mathfrak {t}}^{\mu }-\mathfrak {q}^{\mu })^{\gamma -1}\\&\qquad \times \Biggl (c_{\mathfrak {j}}|\texttt {y}^{\prime }_{\mathfrak {j}}(\mathfrak {q})-\texttt {y}_{\mathfrak {j}}(\mathfrak {q})|+{\sum }_{\ell =1}^{n}|\mathfrak {s}_{\mathfrak {j}\ell }^{I}||\varphi _{\ell }^{R}(\texttt {x}_{\ell }^{\prime },\texttt {y}_{\ell }^{\prime })-\varphi _{\ell }^{R}(\texttt {x}_{\ell },\texttt {y}_{\ell })|+{\sum }_{\ell =1}^{n}|\mathfrak {s}_{\mathfrak {j}\ell }^{R}||\varphi _{\ell }^{I}(\texttt {x}_{\ell }^{\prime },\texttt {y}_{\ell }^{\prime })-\varphi _{\ell }^{I}(\texttt {x}_{\ell },\texttt {y}_{\ell })|\\&\qquad +{\sum }_{\ell =1}^{n}|\mathfrak {p}_{\mathfrak {j}\ell }^{I}||\varphi _{\ell }^{R}(\texttt {x}_{\ell {\tau }}^{\prime },\texttt {y}_{\ell {\tau }}^{\prime })-\varphi _{\ell }^{R}(\texttt {x}_{\ell {\tau }},\texttt {y}_{\ell {\tau }})|+{\sum }_{\ell =1}^{n}|\mathfrak {p}_{\mathfrak {j}\ell }^{R}||\varphi _{\ell }^{I}(\texttt {x}_{\ell {\tau }}^{\prime },\texttt {y}_{\ell {\tau }}^{\prime })-\varphi _{\ell }^{I}(\texttt {x}_{\ell {\tau }},\texttt {y}_{\ell {\tau }})|\Biggr )d\mathfrak {q}\\&\quad \precsim |\Theta ^{\prime }_{\mathfrak {j}}(0)-\Theta _{\mathfrak {j}}(0)|+ \frac{c_{\mathfrak {j}}\mu ^{1-\gamma }}{\Gamma (\gamma )}\int _{0}^{{\mathfrak {t}}}\mathfrak {q}^{\mu -1}({\mathfrak {t}}^{\mu }-\mathfrak {q}^{\mu })^{\gamma -1}|\texttt {y}^{\prime }_{\mathfrak {j}}(\mathfrak {q})-\texttt {y}_{\mathfrak {j}}(\mathfrak {q})|d\mathfrak {q}\\&\qquad +{\sum }_{\ell =1}^{n}|\mathfrak {s}_{\mathfrak {j}\ell }^{I}|\frac{\mu ^{1-\gamma }}{\Gamma (\gamma )}\int _{0}^{{\mathfrak {t}}}\mathfrak {q}^{\mu -1}({\mathfrak {t}}^{\mu }-\mathfrak {q}^{\mu })^{\gamma -1}\big [\theta _{\ell }^{RR}|\texttt {x}^{\prime }_{\ell }(\mathfrak {q})-\texttt {x}_{\ell }(\mathfrak {q})|+\theta _{\ell }^{RI}|\texttt {y}^{\prime }_{\ell }(\mathfrak {q})-\texttt {y}_{\ell }(\mathfrak {q})|\big ]d\mathfrak {q}\\&\qquad +{\sum }_{\ell =1}^{n}|\mathfrak {s}_{\mathfrak {j}\ell }^{R}|\frac{\mu ^{1-\gamma }}{\Gamma (\gamma )}\int _{0}^{{\mathfrak {t}}}\mathfrak {q}^{\mu -1}({\mathfrak {t}}^{\mu }-\mathfrak {q}^{\mu })^{\gamma -1}\big [\theta _{\ell }^{IR}|\texttt {x}^{\prime }_{\ell }(\mathfrak {q})-\texttt {x}_{\ell }(\mathfrak {q})|+\theta _{\ell }^{II}|\texttt {y}^{\prime }_{\ell }(\mathfrak {q})-\texttt {y}_{\ell }(\mathfrak {q})|\big ]d\mathfrak {q}\\&\qquad +{\sum }_{\ell =1}^{n}|\mathfrak {p}_{\mathfrak {j}\ell }^{I}|\frac{\mu ^{1-\gamma }}{\Gamma (\gamma )}\int _{0}^{{\mathfrak {t}}}\mathfrak {q}^{\mu -1}({\mathfrak {t}}^{\mu }-\mathfrak {q}^{\mu })^{\gamma -1}[\omega _{\ell }^{RR}|\texttt {x}^{\prime }_{\ell {\tau }}(\mathfrak {q})-\texttt {x}_{\ell {\tau }}(\mathfrak {q})|+\omega _{\ell }^{RI}|\texttt {y}^{\prime }_{\ell {\tau }}(\mathfrak {q})-\texttt {y}_{\ell {\tau }}(\mathfrak {q})|\big ]d\mathfrak {q} \end{aligned}$$

$$\begin{aligned}&\qquad +{\sum }_{\ell =1}^{n}|\mathfrak {p}_{\mathfrak {j}\ell }^{R}|\frac{\mu ^{1-\gamma }}{\Gamma (\gamma )}\int _{0}^{{\mathfrak {t}}}\mathfrak {q}^{\mu -1}({\mathfrak {t}}^{\mu }-\mathfrak {q}^{\mu })^{\gamma -1}[\omega _{\ell }^{IR}|\texttt {x}^{\prime }_{\ell {\tau }}(\mathfrak {q})-\texttt {x}_{\ell {\tau }}(\mathfrak {q})|+\omega _{\ell }^{II}|\texttt {y}^{\prime }_{\ell {\tau }}(\mathfrak {q})-\texttt {y}_{\ell {\tau }}(\mathfrak {q})|\big ]d\mathfrak {q}\\&\quad \precsim |\Theta ^{\prime }_{\mathfrak {j}}(0)-\Theta _{\mathfrak {j}}(0)|+\frac{\mu ^{1-\gamma }}{\Gamma (\gamma )}\int _{0}^{{\mathfrak {t}}}\mathfrak {q}^{\mu -1}({\mathfrak {t}}^{\mu }-\mathfrak {q}^{\mu })^{\gamma -1}\\&\qquad \times \Biggl (c_{\mathfrak {j}}|\texttt {y}^{\prime }_{\mathfrak {j}}(\mathfrak {q})-\texttt {y}_{\mathfrak {j}}(\mathfrak {q})|+{\sum }_{\ell =1}^{n}|\mathfrak {s}_{\mathfrak {j}\ell }^{I}||\varphi _{\ell }^{R}(\texttt {x}_{\ell }^{\prime },\texttt {y}_{\ell }^{\prime })-\varphi _{\ell }^{R}(\texttt {x}_{\ell },\texttt {y}_{\ell })|+{\sum }_{\ell =1}^{n}|\mathfrak {s}_{\mathfrak {j}\ell }^{R}||\varphi _{\ell }^{I}(\texttt {x}_{\ell }^{\prime },\texttt {y}_{\ell }^{\prime })-\varphi _{\ell }^{I}(\texttt {x}_{\ell },\texttt {y}_{\ell })|\\&\qquad +{\sum }_{\ell =1}^{n}|\mathfrak {p}_{\mathfrak {j}\ell }^{I}||\varphi _{\ell }^{R}(\texttt {x}_{\ell {\tau }}^{\prime },\texttt {y}_{\ell {\tau }}^{\prime })-\varphi _{\ell }^{R}(\texttt {x}_{\ell {\tau }},\texttt {y}_{\ell {\tau }})|+{\sum }_{\ell =1}^{n}|\mathfrak {p}_{\mathfrak {j}\ell }^{R}||\varphi _{\ell }^{I}(\texttt {x}_{\ell {\tau }}^{\prime },\texttt {y}_{\ell {\tau }}^{\prime })-\varphi _{\ell }^{I}(\texttt {x}_{\ell {\tau }},\texttt {y}_{\ell {\tau }})|\Biggr )d\mathfrak {q} \end{aligned}$$

$$\begin{aligned}&\quad \precsim |\Theta ^{\prime }_{\mathfrak {j}}(0)-\Theta _{\mathfrak {j}}(0)|+ \frac{c_{\mathfrak {j}}\mu ^{1-\gamma }}{\Gamma (\gamma )}\int _{0}^{{\mathfrak {t}}}\mathfrak {q}^{\mu -1}({\mathfrak {t}}^{\mu }-\mathfrak {q}^{\mu })^{\gamma -1}|\texttt {y}^{\prime }_{\mathfrak {j}}(\mathfrak {q})-\texttt {y}_{\mathfrak {j}}(\mathfrak {q})|d\mathfrak {q}\\&\qquad +{\sum }_{\ell =1}^{n}|\mathfrak {s}_{\mathfrak {j}\ell }^{I}|\theta _{\ell }^{RR}\frac{\mu ^{1-\gamma }}{\Gamma (\gamma )}\int _{0}^{{\mathfrak {t}}}\mathfrak {q}^{\mu -1}({\mathfrak {t}}^{\mu }-\mathfrak {q}^{\mu })^{\gamma -1}|\texttt {x}^{\prime }_{\ell }(\mathfrak {q})-\texttt {x}_{\ell }(\mathfrak {q})|d\mathfrak {q}\\&\qquad +{\sum }_{\ell =1}^{n}|\mathfrak {s}_{\mathfrak {j}\ell }^{I}|\theta _{\ell }^{RI}\frac{\mu ^{1-\gamma }}{\Gamma (\gamma )}\int _{0}^{{\mathfrak {t}}}\mathfrak {q}^{\mu -1}({\mathfrak {t}}^{\mu }-\mathfrak {q}^{\mu })^{\gamma -1}|\texttt {y}^{\prime }_{\ell }(\mathfrak {q})-\texttt {y}_{\ell }(\mathfrak {q})|d\mathfrak {q}\\&\qquad +{\sum }_{\ell =1}^{n}|\mathfrak {s}_{\mathfrak {j}\ell }^{R}|\theta _{\ell }^{IR}\frac{\mu ^{1-\gamma }}{\Gamma (\gamma )}\int _{0}^{{\mathfrak {t}}}\mathfrak {q}^{\mu -1}({\mathfrak {t}}^{\mu }-\mathfrak {q}^{\mu })^{\gamma -1}|\texttt {x}^{\prime }_{\ell }(\mathfrak {q})-\texttt {x}_{\ell }(\mathfrak {q})|d\mathfrak {q}\\&\qquad +{\sum }_{\ell =1}^{n}|\mathfrak {s}_{\mathfrak {j}\ell }^{R}|\theta _{\ell }^{II}\frac{\mu ^{1-\gamma }}{\Gamma (\gamma )}\int _{0}^{{\mathfrak {t}}}\mathfrak {q}^{\mu -1}({\mathfrak {t}}^{\mu }-\mathfrak {q}^{\mu })^{\gamma -1}|\texttt {y}^{\prime }_{\ell }(\mathfrak {q})-\texttt {y}_{\ell }(\mathfrak {q})|d\mathfrak {q}\\&\qquad +{\sum 
}_{\ell =1}^{n}|\mathfrak {p}_{\mathfrak {j}\ell }^{I}|\omega _{\ell }^{RR}\frac{\mu ^{1-\gamma }}{\Gamma (\gamma )}\int _{0}^{{\mathfrak {t}}}\mathfrak {q}^{\mu -1}({\mathfrak {t}}^{\mu }-\mathfrak {q}^{\mu })^{\gamma -1}|\texttt {x}^{\prime }_{\ell {\tau }}(\mathfrak {q})-\texttt {x}_{\ell {\tau }}(\mathfrak {q})|d\mathfrak {q}\\&\qquad +{\sum }_{\ell =1}^{n}|\mathfrak {p}_{\mathfrak {j}\ell }^{I}|\omega _{\ell }^{RI}\frac{\mu ^{1-\gamma }}{\Gamma (\gamma )}\int _{0}^{{\mathfrak {t}}}\mathfrak {q}^{\mu -1}({\mathfrak {t}}^{\mu }-\mathfrak {q}^{\mu })^{\gamma -1}|\texttt {y}^{\prime }_{\ell {\tau }}(\mathfrak {q})-\texttt {y}_{\ell {\tau }}(\mathfrak {q})|d\mathfrak {q}\\&\qquad +{\sum }_{\ell =1}^{n}|\mathfrak {p}_{\mathfrak {j}\ell }^{R}|\omega _{\ell }^{IR}\frac{\mu ^{1-\gamma }}{\Gamma (\gamma )}\int _{0}^{{\mathfrak {t}}}\mathfrak {q}^{\mu -1}({\mathfrak {t}}^{\mu }-\mathfrak {q}^{\mu })^{\gamma -1}|\texttt {x}^{\prime }_{\ell {\tau }}(\mathfrak {q})-\texttt {x}_{\ell {\tau }}(\mathfrak {q})|d\mathfrak {q}\\&\qquad +{\sum }_{\ell =1}^{n}|\mathfrak {p}_{\mathfrak {j}\ell }^{R}|\omega _{\ell }^{II}\frac{\mu ^{1-\gamma }}{\Gamma (\gamma )}\int _{0}^{{\mathfrak {t}}}\mathfrak {q}^{\mu -1}({\mathfrak {t}}^{\mu }-\mathfrak {q}^{\mu })^{\gamma -1}|\texttt {y}^{\prime }_{\ell {\tau }}(\mathfrak {q})-\texttt {y}_{\ell {\tau }}(\mathfrak {q})|d\mathfrak {q}\\&\quad \precsim |\Theta ^{\prime }_{\mathfrak {j}}(0)-\Theta _{\mathfrak {j}}(0)|+ \frac{c_{\mathfrak {j}}\mu ^{1-\gamma }}{\Gamma (\gamma )}\int _{0}^{{\mathfrak {t}}}\mathfrak {q}^{\mu -1}({\mathfrak {t}}^{\mu }-\mathfrak {q}^{\mu })^{\gamma -1}|\texttt {y}^{\prime }_{\mathfrak {j}}(\mathfrak {q})-\texttt {y}_{\mathfrak {j}}(\mathfrak {q})|d\mathfrak {q} \end{aligned}$$

$$\begin{aligned}&\qquad +{\sum }_{\ell =1}^{n}|\mathfrak {s}_{\mathfrak {j}\ell }^{I}|\theta _{\ell }^{RR}\frac{\mu ^{1-\gamma }}{\Gamma (\gamma )}\int _{0}^{{\mathfrak {t}}}\mathfrak {q}^{\mu -1}({\mathfrak {t}}^{\mu }-\mathfrak {q}^{\mu })^{\gamma -1}|\texttt {x}^{\prime }_{\ell }(\mathfrak {q})-\texttt {x}_{\ell }(\mathfrak {q})|d\mathfrak {q}\\&\qquad +{\sum }_{\ell =1}^{n}|\mathfrak {s}_{\mathfrak {j}\ell }^{I}|\theta _{\ell }^{RI}\frac{\mu ^{1-\gamma }}{\Gamma (\gamma )}\int _{0}^{{\mathfrak {t}}}\mathfrak {q}^{\mu -1}({\mathfrak {t}}^{\mu }-\mathfrak {q}^{\mu })^{\gamma -1}|\texttt {y}^{\prime }_{\ell }(\mathfrak {q})-\texttt {y}_{\ell }(\mathfrak {q})|d\mathfrak {q}\\&\qquad +{\sum }_{\ell =1}^{n}|\mathfrak {s}_{\mathfrak {j}\ell }^{R}|\theta _{\ell }^{IR}\frac{\mu ^{1-\gamma }}{\Gamma (\gamma )}\int _{0}^{{\mathfrak {t}}}\mathfrak {q}^{\mu -1}({\mathfrak {t}}^{\mu }-\mathfrak {q}^{\mu })^{\gamma -1}|\texttt {x}^{\prime }_{\ell }(\mathfrak {q})-\texttt {x}_{\ell }(\mathfrak {q})|d\mathfrak {q}\\&\qquad +{\sum }_{\ell =1}^{n}|\mathfrak {s}_{\mathfrak {j}\ell }^{R}|\theta _{\ell }^{II}\frac{\mu ^{1-\gamma }}{\Gamma (\gamma )}\int _{0}^{{\mathfrak {t}}}\mathfrak {q}^{\mu -1}({\mathfrak {t}}^{\mu }-\mathfrak {q}^{\mu })^{\gamma -1}|\texttt {y}^{\prime }_{\ell }(\mathfrak {q})-\texttt {y}_{\ell }(\mathfrak {q})|d\mathfrak {q}\\&\qquad +{\sum }_{\ell =1}^{n}|\mathfrak {p}_{\mathfrak {j}\ell }^{I}|\omega _{\ell }^{RR}\frac{\mu ^{1-\gamma }}{\Gamma (\gamma )}\int _{0}^{{\tau }}\mathfrak {q}^{\mu -1}({\mathfrak {t}}^{\mu }-\mathfrak {q}^{\mu })^{\gamma -1}|\Psi ^{\prime }_{\ell {\tau }}(\mathfrak {q})-\Psi _{\ell {\tau }}(\mathfrak {q})|d\mathfrak {q}\\&\qquad +{\sum }_{\ell =1}^{n}|\mathfrak {p}_{\mathfrak {j}\ell }^{I}|\omega _{\ell }^{RR}\frac{\mu ^{1-\gamma }}{\Gamma (\gamma )}\int _{{\tau }}^{{\mathfrak {t}}}\mathfrak {q}^{\mu -1}({\mathfrak {t}}^{\mu }-\mathfrak {q}^{\mu })^{\gamma -1}|\texttt {x}^{\prime }_{\ell {\tau }}(\mathfrak {q})-\texttt {x}_{\ell {\tau }}(\mathfrak {q})|d\mathfrak {q}\\&\qquad +{\sum }_{\ell =1}^{n}|\mathfrak {p}_{\mathfrak {j}\ell }^{I}|\omega _{\ell }^{RI}\frac{\mu ^{1-\gamma }}{\Gamma (\gamma )}\int _{0}^{{\tau }}\mathfrak {q}^{\mu -1}({\mathfrak {t}}^{\mu }-\mathfrak {q}^{\mu })^{\gamma -1}|\Theta ^{\prime }_{\ell {\tau }}(\mathfrak {q})-\Theta _{\ell {\tau }}(\mathfrak {q})|d\mathfrak {q}\\&\qquad +{\sum }_{\ell =1}^{n}|\mathfrak {p}_{\mathfrak {j}\ell }^{I}|\omega _{\ell }^{RI}\frac{\mu ^{1-\gamma }}{\Gamma (\gamma )}\int _{{\tau }}^{{\mathfrak {t}}}\mathfrak {q}^{\mu -1}({\mathfrak {t}}^{\mu }-\mathfrak {q}^{\mu })^{\gamma -1}|\texttt {y}^{\prime }_{\ell {\tau }}(\mathfrak {q})-\texttt {y}_{\ell {\tau }}(\mathfrak {q})|d\mathfrak {q}\\&\qquad +{\sum }_{\ell =1}^{n}|\mathfrak {p}_{\mathfrak {j}\ell }^{R}|\omega _{\ell }^{IR}\frac{\mu ^{1-\gamma }}{\Gamma (\gamma )}\int _{0}^{{\tau }}\mathfrak {q}^{\mu -1}({\mathfrak {t}}^{\mu }-\mathfrak {q}^{\mu })^{\gamma -1}|\Psi ^{\prime }_{\ell {\tau }}(\mathfrak {q})-\Psi _{\ell {\tau }}(\mathfrak {q})|d\mathfrak {q}\\&\qquad +{\sum }_{\ell =1}^{n}|\mathfrak {p}_{\mathfrak {j}\ell }^{R}|\omega _{\ell }^{IR}\frac{\mu ^{1-\gamma }}{\Gamma (\gamma )}\int _{{\tau }}^{{\mathfrak {t}}}\mathfrak {q}^{\mu -1}({\mathfrak {t}}^{\mu }-\mathfrak {q}^{\mu })^{\gamma -1}|\texttt {x}^{\prime }_{\ell {\tau }}(\mathfrak {q})-\texttt {x}_{\ell {\tau }}(\mathfrak {q})|d\mathfrak {q}\\&\qquad +{\sum }_{\ell =1}^{n}|\mathfrak {p}_{\mathfrak {j}\ell }^{R}|\omega _{\ell }^{II}\frac{\mu ^{1-\gamma }}{\Gamma (\gamma )}\int _{0}^{{\tau }}\mathfrak {q}^{\mu -1}({\mathfrak {t}}^{\mu }-\mathfrak {q}^{\mu })^{\gamma -1}|\Theta ^{\prime }_{\ell {\tau }}(\mathfrak {q})-\Theta _{\ell {\tau }}(\mathfrak {q})|d\mathfrak {q}\\&\qquad +{\sum }_{\ell =1}^{n}|\mathfrak {p}_{\mathfrak {j}\ell }^{I}|\omega _{\ell }^{II}\frac{\mu ^{1-\gamma }}{\Gamma (\gamma )}\int _{{\tau }}^{{\mathfrak {t}}}\mathfrak {q}^{\mu -1}({\mathfrak {t}}^{\mu }-\mathfrak {q}^{\mu })^{\gamma -1}|\texttt {y}^{\prime }_{\ell {\tau }}(\mathfrak {q})-\texttt {y}_{\ell {\tau }}(\mathfrak {q})|d\mathfrak {q}\\ \end{aligned}$$

Which yields,

$$\begin{aligned}&{e }^{\mathfrak {i}({\mathfrak {t}}-1)}|\texttt {y}^{\prime }_{\mathfrak {j}}({\mathfrak {t}})-\texttt {y}_{\mathfrak {j}}({\mathfrak {t}})|\\&\quad \precsim {\sup }_{{\mathfrak {t}}}\{{e }^{\mathfrak {i}({\mathfrak {t}}-1)}|\Theta ^{\prime }_{\mathfrak {j}}({\mathfrak {t}})-\Theta _{\mathfrak {j}}({\mathfrak {t}})|\}+c_{\mathfrak {j}}\frac{\mu ^{1-\gamma }}{\Gamma (\gamma )}{\sup }_{{\mathfrak {t}}}\{{e }^{\mathfrak {i}({\mathfrak {t}}-1)}|(\texttt {y}^{\prime }_{\mathfrak {j}}({\mathfrak {t}})-\texttt {y}_{\mathfrak {j}}({\mathfrak {t}}))|\}\int _{0}^{{\mathfrak {t}}}\kappa ^{\mu -1}({\mathfrak {t}}^{\mu }-\kappa ^{\mu })^{\gamma -1}d\kappa \\&\qquad +[\mathfrak {q}_{1\mathfrak {j}}^{\star }+\mathfrak {q}_{3\mathfrak {j}}^{\star }]{\sum }_{\ell =1}^{n}{\sup }_{{\mathfrak {t}}}\{{e }^{\mathfrak {i}({\mathfrak {t}}-1)}|\texttt {x}^{\prime }_{\ell }({\mathfrak {t}})-\texttt {x}_{\ell }({\mathfrak {t}})|\}\frac{\mu ^{1-\gamma }}{\Gamma (\gamma )}\int _{0}^{{\mathfrak {t}}}\kappa ^{\mu -1}({\mathfrak {t}}^{\mu }-\kappa ^{\mu })^{\gamma -1}d\kappa \\&\qquad +[\mathfrak {q}_{2\mathfrak {j}}^{\star }+\mathfrak {q}_{4\mathfrak {j}}^{\star }]{\sum }_{\ell =1}^{n}{\sup }_{{\mathfrak {t}}}\{{e }^{\mathfrak {i}({\mathfrak {t}}-1)}|\texttt {y}^{\prime }_{\ell }({\mathfrak {t}})-\texttt {y}_{\ell }({\mathfrak {t}})|\}\frac{\mu ^{1-\gamma }}{\Gamma (\gamma )}\int _{0}^{{\mathfrak {t}}}\kappa ^{\mu -1}({\mathfrak {t}}^{\mu }-\kappa ^{\mu })^{\gamma -1}d\kappa \\&\qquad +[{\mathfrak {t}}_{1\mathfrak {j}}^{\star }+{\mathfrak {t}}_{3\mathfrak {j}}^{\star }]{\sum }_{\ell =1}^{n}\frac{\mu ^{1-\gamma }}{\Gamma (\gamma )}\int _{0}^{{\tau }}\wp ^{\mu -1}({\mathfrak {t}}^{\mu }-\wp ^{\mu })^{\gamma -1}{e }^{\mathfrak {i}(\wp -1)}|\Psi ^{\prime }_{\ell }(\wp )-\Psi _{\ell }(\wp )|d\wp \\&\qquad +[{\mathfrak {t}}_{1\mathfrak {j}}^{\star }+{\mathfrak {t}}_{3\mathfrak {j}}^{\star }]{\sum }_{\ell =1}^{n}\frac{\mu ^{1-\gamma }}{\Gamma (\gamma )}\int _{{\tau }}^{{\mathfrak {t}}}\wp ^{\mu -1}({\mathfrak {t}}^{\mu }-\wp ^{\mu })^{\gamma -1}{e }^{\mathfrak {i}(\wp -1)}|\texttt {x}^{\prime }_{\ell }(\wp )-\texttt {x}_{\ell }(\wp )|d\wp \\&\qquad +[{\mathfrak {t}}_{2\mathfrak {j}}^{\star }+{\mathfrak {t}}_{4\mathfrak {j}}^{\star }]{\sum }_{\ell =1}^{n}\frac{\mu ^{1-\gamma }}{\Gamma (\gamma )}\int _{{\tau }}^{{\mathfrak {t}}}\wp ^{\mu -1}({\mathfrak {t}}^{\mu }-\wp ^{\mu })^{\gamma -1}{e }^{\mathfrak {i}(\wp -1)}|\texttt {y}^{\prime }_{\ell }(\wp )-\texttt {y}_{\ell }(\wp )|d\wp \\&\qquad +[{\mathfrak {t}}_{2\mathfrak {j}}^{\star }+{\mathfrak {t}}_{4\mathfrak {j}}^{\star }]{\sum }_{\ell =1}^{n}\frac{\mu ^{1-\gamma }}{\Gamma (\gamma )}\int _{0}^{{\tau }}\wp ^{\mu -1}({\mathfrak {t}}^{\mu }-\wp ^{\mu })^{\gamma -1}{e }^{\mathfrak {i}(\wp -1)}|\Theta ^{\prime }_{\ell }(\wp )-\Theta _{\ell }(\wp )|d\wp \\&\quad \precsim {\sup }_{{\mathfrak {t}}}\{{e }^{\mathfrak {i}({\mathfrak {t}}-1)}|\Theta ^{\prime }_{\mathfrak {j}}({\mathfrak {t}})-\Theta _{\mathfrak {j}}({\mathfrak {t}})|\}+c_{\mathfrak {j}}\frac{\mu ^{1-\gamma }}{\Gamma (\gamma )}{\sup }_{{\mathfrak {t}}}\{{e }^{\mathfrak {i}({\mathfrak {t}}-1)}|(\texttt {y}^{\prime }_{\mathfrak {j}}({\mathfrak {t}})-\texttt {y}_{\mathfrak {j}}({\mathfrak {t}}))|\}\int _{0}^{{\mathfrak {t}}}\kappa ^{\mu -1}({\mathfrak {t}}^{\mu }-\kappa ^{\mu })^{\gamma -1}d\kappa \\&\qquad +[\mathfrak {q}_{1\mathfrak {j}}^{\star }+\mathfrak {q}_{3\mathfrak {j}}^{\star }]{\sum }_{\ell =1}^{n}{\sup }_{{\mathfrak {t}}}\{{e }^{\mathfrak {i}({\mathfrak {t}}-1)}|\texttt {x}^{\prime }_{\ell }({\mathfrak {t}})-\texttt {x}_{\ell }({\mathfrak {t}})|\}\frac{\mu ^{1-\gamma }}{\Gamma (\gamma )}\int _{0}^{{\mathfrak {t}}}\kappa ^{\mu -1}({\mathfrak {t}}^{\mu }-\kappa ^{\mu })^{\gamma -1}d\kappa \end{aligned}$$

$$\begin{aligned}&\qquad +[\mathfrak {q}_{2\mathfrak {j}}^{\star }+\mathfrak {q}_{4\mathfrak {j}}^{\star }]{\sum }_{\ell =1}^{n}{\sup }_{{\mathfrak {t}}}\{{e }^{\mathfrak {i}({\mathfrak {t}}-1)}|\texttt {y}^{\prime }_{\ell }({\mathfrak {t}})-\texttt {y}_{\ell }({\mathfrak {t}})|\}\frac{\mu ^{1-\gamma }}{\Gamma (\gamma )}\int 
_{0}^{{\mathfrak {t}}}\kappa ^{\mu -1}({\mathfrak {t}}^{\mu }-\kappa ^{\mu })^{\gamma -1}d\kappa \\&\qquad +[{\mathfrak {t}}_{1\mathfrak {j}}^{\star }+{\mathfrak {t}}_{3\mathfrak {j}}^{\star }]{\sum }_{\ell =1}^{n}{\sup }_{{\mathfrak {t}}}\{{e }^{\mathfrak {i}({\mathfrak {t}}-1)}|\Psi ^{\prime }_{\ell }({\mathfrak {t}})-\Psi _{\ell }({\mathfrak {t}})|\}\frac{\mu ^{1-\gamma }}{\Gamma (\gamma )}\int _{0}^{{\tau }}\wp ^{\mu -1}({\mathfrak {t}}^{\mu }-\wp ^{\mu })^{\gamma -1}d\wp \\&\qquad +[{\mathfrak {t}}_{1\mathfrak {j}}^{\star }+{\mathfrak {t}}_{3\mathfrak {j}}^{\star }]{\sum }_{\ell =1}^{n}{\sup }_{{\mathfrak {t}}}\{{e }^{\mathfrak {i}({\mathfrak {t}}-1)}|\texttt {x}^{\prime }_{\ell }({\mathfrak {t}})-\texttt {x}_{\ell }({\mathfrak {t}})|\}\frac{\mu ^{1-\gamma }}{\Gamma (\gamma )}\int _{{\tau }}^{{\mathfrak {t}}}\wp ^{\mu -1}({\mathfrak {t}}^{\mu }-\wp ^{\mu })^{\gamma -1}d\wp \\&\qquad +[{\mathfrak {t}}_{2\mathfrak {j}}^{\star }+{\mathfrak {t}}_{4\mathfrak {j}}^{\star }]{\sum }_{\ell =1}^{n}{\sup }_{{\mathfrak {t}}}\{{e }^{\mathfrak {i}({\mathfrak {t}}-1)}|\Theta ^{\prime }_{\ell }({\mathfrak {t}})-\Theta _{\ell }({\mathfrak {t}})|\}\frac{\mu ^{1-\gamma }}{\Gamma (\gamma )}\int _{0}^{{\tau }}\wp ^{\mu -1}({\mathfrak {t}}^{\mu }-\wp ^{\mu })^{\gamma -1}d\wp \\&\qquad +[{\mathfrak {t}}_{2\mathfrak {j}}^{\star }+{\mathfrak {t}}_{4\mathfrak {j}}^{\star }]{\sum }_{\ell =1}^{n}{\sup }_{{\mathfrak {t}}}\{{e }^{\mathfrak {i}({\mathfrak {t}}-1)}|\texttt {y}^{\prime }_{\ell }({\mathfrak {t}})-\texttt {y}_{\ell }({\mathfrak {t}})|\}\frac{\mu ^{1-\gamma }}{\Gamma (\gamma )}\int _{{\tau }}^{{\mathfrak {t}}}\wp ^{\mu -1}({\mathfrak {t}}^{\mu }-\wp ^{\mu })^{\gamma -1}d\wp \\&\quad \precsim {\sup }_{{\mathfrak {t}}}\{{e }^{\mathfrak {i}({\mathfrak {t}}-1)}|\Theta ^{\prime }_{\mathfrak {j}}({\mathfrak {t}})-\Theta _{\mathfrak {j}}({\mathfrak {t}})|\}+c_{\mathfrak {j}}\frac{\mu ^{1-\gamma }}{\Gamma (\gamma )}{\sup }_{{\mathfrak {t}}}\{{e }^{\mathfrak {i}({\mathfrak {t}}-1)}|(\texttt {y}^{\prime }_{\mathfrak {j}}({\mathfrak {t}})-\texttt {y}_{\mathfrak {j}}({\mathfrak {t}}))|\}\int _{0}^{{\mathfrak {t}}}\kappa ^{\mu -1}({\mathfrak {t}}^{\mu }-\kappa ^{\mu })^{\gamma -1}d\kappa \\&\qquad +[\mathfrak {q}_{1\mathfrak {j}}^{\star }+\mathfrak {q}_{3\mathfrak {j}}^{\star }]{\sum }_{\ell =1}^{n}{\sup }_{{\mathfrak {t}}}\{{e }^{\mathfrak {i}({\mathfrak {t}}-1)}|\texttt {x}^{\prime }_{\ell }({\mathfrak {t}})-\texttt {x}_{\ell }({\mathfrak {t}})|\}\frac{\mu ^{1-\gamma }}{\Gamma (\gamma )}\int _{0}^{{\mathfrak {t}}}\kappa ^{\mu -1}({\mathfrak {t}}^{\mu }-\kappa ^{\mu })^{\gamma -1}d\kappa \\&\qquad +[\mathfrak {q}_{2\mathfrak {j}}^{\star }+\mathfrak {q}_{4\mathfrak {j}}^{\star }]{\sum }_{\ell =1}^{n}{\sup }_{{\mathfrak {t}}}\{{e }^{\mathfrak {i}({\mathfrak {t}}-1)}|\texttt {y}^{\prime }_{\ell }({\mathfrak {t}})-\texttt {y}_{\ell }({\mathfrak {t}})|\}\frac{\mu ^{1-\gamma }}{\Gamma (\gamma )}\int _{0}^{{\mathfrak {t}}}\kappa ^{\mu -1}({\mathfrak {t}}^{\mu }-\kappa ^{\mu })^{\gamma -1}d\kappa \\&\qquad +[{\mathfrak {t}}_{1\mathfrak {j}}^{\star }+{\mathfrak {t}}_{3\mathfrak {j}}^{\star }]\frac{\mu ^{1-\gamma }}{\Gamma (\gamma )}{\sum }_{\ell =1}^{n}\int _{-{\tau }}^{0}(\mathfrak {q}+{\tau })^{\mu -1}[{\mathfrak {t}}^{\mu }-(\mathfrak {q}+{\tau })^{\mu }]^{\gamma -1}{e }^{\mathfrak {i}(\mathfrak {q}-1)}|(\Psi ^{\prime }_{\ell }(\mathfrak {q})-\Psi _{\ell }(\mathfrak {q}))|d\mathfrak {q}\\&\qquad +[{\mathfrak {t}}_{1\mathfrak {j}}^{\star }+{\mathfrak {t}}_{3\mathfrak {j}}^{\star }]\frac{\mu ^{1-\gamma }}{\Gamma (\gamma )}{\sum }_{\ell =1}^{n}\int _{0}^{{\mathfrak {t}}-{\tau }}(\mathfrak {q}+{\tau })^{\mu -1}[{\mathfrak {t}}^{\mu }-(\mathfrak {q}+{\tau })^{\mu }]^{\gamma -1}{e }^{\mathfrak {i}(\mathfrak {q}-1)}|(\texttt {x}^{\prime }_{\ell }(\mathfrak {q})-\texttt {x}_{\ell }(\mathfrak {q}))|d\mathfrak {q} \end{aligned}$$

$$\begin{aligned}&\qquad +[{\mathfrak {t}}_{2\mathfrak {j}}^{\star }+{\mathfrak {t}}_{4\mathfrak {j}}^{\star }]\frac{\mu ^{1-\gamma }}{\Gamma (\gamma )}{\sum }_{\ell =1}^{n}\int _{-{\tau }}^{0}(\mathfrak {q}+{\tau })^{\mu -1}[{\mathfrak {t}}^{\mu }-(\mathfrak {q}+{\tau })^{\mu }]^{\gamma -1}{e }^{\mathfrak {i}(\mathfrak {q}-1)}|(\Theta ^{\prime }_{\ell }(\mathfrak {q})-\Theta _{\ell }(\mathfrak {q}))|d\mathfrak {q}\\&\qquad +[{\mathfrak {t}}_{2\mathfrak {j}}^{\star }+{\mathfrak {t}}_{4\mathfrak {j}}^{\star }]\frac{\mu ^{1-\gamma }}{\Gamma (\gamma )}{\sum }_{\ell =1}^{n}\int _{0}^{{\mathfrak {t}}-{\tau }}(\mathfrak {q}+{\tau })^{\mu -1}[{\mathfrak {t}}^{\mu }-(\mathfrak {q}+{\tau })^{\mu }]^{\gamma -1}{e }^{\mathfrak {i}(\mathfrak {q}-1)}|(\texttt {y}^{\prime }_{\ell }(\mathfrak {q})-\texttt {y}_{\ell }(\mathfrak {q}))|d\mathfrak {q}\\&\quad \precsim {\sup }_{{\mathfrak {t}}}\{{e }^{\mathfrak {i}({\mathfrak {t}}-1)}|\Theta ^{\prime }_{\mathfrak {j}}({\mathfrak {t}})-\Theta _{\mathfrak {j}}({\mathfrak {t}})|\}+c_{\mathfrak {j}}\frac{\mu ^{1-\gamma }}{\Gamma (\gamma )}{\sup }_{{\mathfrak {t}}}\{{e }^{\mathfrak {i}({\mathfrak {t}}-1)}|(\texttt {y}^{\prime }_{\mathfrak {j}}({\mathfrak {t}})-\texttt {y}_{\mathfrak {j}}({\mathfrak {t}}))|\}\int _{0}^{{\mathfrak {t}}}\kappa ^{\mu -1}({\mathfrak {t}}^{\mu }-\kappa ^{\mu })^{\gamma -1}d\kappa \\&\qquad +[\mathfrak {q}_{1\mathfrak {j}}^{\star }+\mathfrak {q}_{3\mathfrak {j}}^{\star }]{\sum }_{\ell =1}^{n}{\sup }_{{\mathfrak {t}}}\{{e }^{\mathfrak {i}({\mathfrak {t}}-1)}|\texttt {x}^{\prime }_{\ell }({\mathfrak {t}})-\texttt {x}_{\ell }({\mathfrak {t}})|\}\frac{\mu ^{1-\gamma }}{\Gamma (\gamma )}\int _{0}^{{\mathfrak {t}}}\kappa ^{\mu -1}({\mathfrak {t}}^{\mu }-\kappa ^{\mu })^{\gamma -1}d\kappa \\&\qquad +[\mathfrak {q}_{2\mathfrak {j}}^{\star }+\mathfrak {q}_{4\mathfrak {j}}^{\star }]{\sum }_{\ell =1}^{n}{\sup }_{{\mathfrak {t}}}\{{e }^{\mathfrak {i}({\mathfrak {t}}-1)}|\texttt {y}^{\prime }_{\ell }({\mathfrak {t}})-\texttt {y}_{\ell }({\mathfrak {t}})|\}\frac{\mu ^{1-\gamma }}{\Gamma (\gamma )}\int _{0}^{{\mathfrak {t}}}\kappa ^{\mu -1}({\mathfrak {t}}^{\mu }-\kappa ^{\mu })^{\gamma -1}d\kappa \\&\qquad +[{\mathfrak {t}}_{1\mathfrak {j}}^{\star }+{\mathfrak {t}}_{3\mathfrak {j}}^{\star }]\frac{\mu ^{1-\gamma }}{\Gamma (\gamma )}{\sum }_{\ell =1}^{n}{e }^{\mathfrak {i}({\mathfrak {t}}-1)}|(\Psi ^{\prime }_{\ell }({\mathfrak {t}})-\Psi _{\ell }({\mathfrak {t}}))|\int _{{\mathfrak {t}}^{\mu }-{\tau }^{\mu }}^{{\mathfrak {t}}^{\mu }}{z}^{\gamma -1}d{z}\\&\qquad +[{\mathfrak {t}}_{1\mathfrak {j}}^{\star }+{\mathfrak {t}}_{3\mathfrak {j}}^{\star }]\frac{\mu ^{1-\gamma }}{\Gamma (\gamma )}{\sum }_{\ell =1}^{n}{e }^{\mathfrak {i}({\mathfrak {t}}-1)}|(\texttt {x}^{\prime }_{\ell }({\mathfrak {t}})-\texttt {x}_{\ell }({\mathfrak {t}}))|\int _{0}^{{\mathfrak {t}}^{\mu }-{\tau }^{\mu }}{z}^{\gamma -1}d{z}\\&\qquad +[{\mathfrak {t}}_{2\mathfrak {j}}^{\star }+{\mathfrak {t}}_{4\mathfrak {j}}^{\star }]\frac{\mu ^{1-\gamma }}{\Gamma (\gamma )}{\sum }_{\ell =1}^{n}{e }^{\mathfrak {i}({\mathfrak {t}}-1)}|(\Theta ^{\prime }_{\ell }({\mathfrak {t}})-\Theta _{\ell }({\mathfrak {t}}))|\int _{{\mathfrak {t}}^{\mu }-{\tau }^{\mu }}^{{\mathfrak {t}}^{\mu }}{z}^{\gamma -1}d{z}\\&\qquad +[{\mathfrak {t}}_{2\mathfrak {j}}^{\star }+{\mathfrak {t}}_{4\mathfrak {j}}^{\star }]\frac{\mu ^{1-\gamma }}{\Gamma (\gamma )}{\sum }_{\ell =1}^{n}{e }^{\mathfrak {i}({\mathfrak {t}}-1)}|(\texttt {y}^{\prime }_{\ell }({\mathfrak {t}})-\texttt {y}_{\ell }({\mathfrak {t}}))|\int _{0}^{{\mathfrak {t}}^{\mu }-{\tau }^{\mu }}{z}^{\gamma -1}d{z} \end{aligned}$$

$$\begin{aligned}&\quad \precsim {\sup }_{{\mathfrak {t}}}\{{e }^{\mathfrak {i}({\mathfrak {t}}-1)}|\Theta ^{\prime }_{\mathfrak {j}}({\mathfrak {t}})-\Theta _{\mathfrak {j}}({\mathfrak {t}})|\}+\frac{\mu ^{1-\gamma }}{\Gamma (\gamma )}\Biggl [c_{\mathfrak {j}}{\sup }_{{\mathfrak {t}}}\{{e }^{\mathfrak {i}({\mathfrak {t}}-1)}|(\texttt {y}^{\prime }_{\mathfrak {j}}({\mathfrak {t}})-\texttt {y}_{\mathfrak {j}}({\mathfrak {t}}))|\}\frac{{\mathfrak {t}}^{\gamma \mu }}{\gamma \mu }\\&\qquad +[\mathfrak {q}_{1\mathfrak {j}}^{\star }+\mathfrak {q}_{3\mathfrak {j}}^{\star }]{\sum }_{\ell =1}^{n}{\sup }_{{\mathfrak {t}}}\{{e }^{\mathfrak {i}({\mathfrak {t}}-1)}|\texttt {x}^{\prime }_{\ell }({\mathfrak {t}})-\texttt {x}_{\ell }({\mathfrak {t}})|\}\frac{{\mathfrak {t}}^{\gamma \mu }}{\gamma \mu }+[\mathfrak {q}_{2\mathfrak {j}}^{\star }+\mathfrak {q}_{4\mathfrak {j}}^{\star }]{\sum }_{\ell =1}^{n}{\sup }_{{\mathfrak {t}}}\{{e }^{\mathfrak {i}({\mathfrak {t}}-1)}|\texttt {y}^{\prime }_{\ell }({\mathfrak {t}})-\texttt {y}_{\ell }({\mathfrak {t}})|\}\frac{{\mathfrak {t}}^{\gamma \mu }}{\gamma \mu }\\&\qquad +[{\mathfrak {t}}_{1\mathfrak {j}}^{\star }+{\mathfrak {t}}_{3\mathfrak {j}}^{\star }]{\sum }_{\ell =1}^{n}{\sup }_{{\mathfrak {t}}}\{{e }^{\mathfrak {i}({\mathfrak {t}}-1)}|\Psi ^{\prime }_{\ell }({\mathfrak {t}})-\Psi _{\ell }({\mathfrak {t}})|\}\biggl (\frac{{\mathfrak {t}}^{\gamma \mu }}{\gamma \mu }-\frac{({\mathfrak {t}}^{\mu }-{\tau }^{\mu })^{\gamma }}{\gamma \mu }\biggr )\\&\qquad +[{\mathfrak {t}}_{1\mathfrak {j}}^{\star }+{\mathfrak {t}}_{3\mathfrak {j}}^{\star }]{\sum }_{\ell =1}^{n}{\sup }_{{\mathfrak {t}}}\{{e }^{\mathfrak {i}({\mathfrak {t}}-1)}|\texttt {x}^{\prime }_{\ell }({\mathfrak {t}})-\texttt {x}_{\ell }({\mathfrak {t}})|\}\biggl (\frac{({\mathfrak {t}}^{\mu }-{\tau }^{\mu })^{\gamma }}{\gamma \mu }\biggr )\\&\qquad +[{\mathfrak {t}}_{2\mathfrak {j}}^{\star }+{\mathfrak {t}}_{4\mathfrak {j}}^{\star }]{\sum }_{\ell =1}^{n}{\sup }_{{\mathfrak {t}}}\{{e }^{\mathfrak {i}({\mathfrak {t}}-1)}|\Theta ^{\prime }_{\ell }({\mathfrak {t}})-\Theta _{\ell }({\mathfrak {t}})|\}\biggl (\frac{{\mathfrak {t}}^{\gamma \mu }}{\gamma \mu }-\frac{({\mathfrak {t}}^{\mu }-{\tau }^{\mu })^{\gamma }}{\gamma \mu }\biggr )\\&\qquad +[{\mathfrak {t}}_{2\mathfrak {j}}^{\star }+{\mathfrak {t}}_{4\mathfrak {j}}^{\star }]{\sum }_{\ell =1}^{n}{\sup }_{{\mathfrak {t}}}\{{e }^{\mathfrak {i}({\mathfrak {t}}-1)}|\texttt {y}^{\prime }_{\ell }({\mathfrak {t}})-\texttt {y}_{\ell }({\mathfrak {t}})|\}\biggl (\frac{({\mathfrak {t}}^{\mu }-{\tau }^{\mu })^{\gamma 
}}{\gamma \mu }\biggr )\Biggr ]\\&\quad \precsim {\sup }_{{\mathfrak {t}}}\{{e }^{\mathfrak {i}({\mathfrak {t}}-1)}|\Theta ^{\prime }_{\mathfrak {j}}({\mathfrak {t}})-\Theta _{\mathfrak {j}}({\mathfrak {t}})|\}+\frac{{\mathfrak {t}}^{\gamma \mu }}{\mu ^{\gamma }\Gamma (\gamma +1)}c_{\mathfrak {j}}{\sup }_{{\mathfrak {t}}}\{{e }^{\mathfrak {i}({\mathfrak {t}}-1)}|\texttt {y}^{\prime }_{\mathfrak {j}}({\mathfrak {t}})-\texttt {y}_{\mathfrak {j}}({\mathfrak {t}})|\}\\&\qquad +\frac{{\mathfrak {t}}^{\gamma \mu }}{\mu ^{\gamma }\Gamma (\gamma +1)}[\mathfrak {q}_{1\mathfrak {j}}^{\star }+\mathfrak {q}_{3\mathfrak {j}}^{\star }]||\texttt {x}^{\prime }({\mathfrak {t}})-\texttt {x}({\mathfrak {t}})||++\frac{{\mathfrak {t}}^{\gamma \mu }}{\mu ^{\gamma }\Gamma (\gamma +1)}[\mathfrak {q}_{2\mathfrak {j}}^{\star }+\mathfrak {q}_{4\mathfrak {j}}^{\star }]||\texttt {y}^{\prime }({\mathfrak {t}})-\texttt {y}({\mathfrak {t}})||\\&\qquad +\frac{{\mathfrak {t}}^{\gamma \mu }}{\mu ^{\gamma }\Gamma (\gamma +1)}[{\mathfrak {t}}_{1\mathfrak {j}}^{\star }+{\mathfrak {t}}_{3\mathfrak {j}}^{\star }]||\Psi ^{\prime }({\mathfrak {t}})-\Psi ({\mathfrak {t}})||+\frac{({\mathfrak {t}}^{\mu }-{\tau }^{\mu })^{\gamma }}{\mu ^{\gamma }\Gamma (\gamma +1)}[{\mathfrak {t}}_{1\mathfrak {j}}^{\star }+{\mathfrak {t}}_{3\mathfrak {j}}^{\star }]||\Psi ^{\prime }({\mathfrak {t}})-\Psi ({\mathfrak {t}})||\\&\qquad +\frac{({\mathfrak {t}}^{\mu }-{\tau }^{\mu })^{\gamma }}{\mu ^{\gamma }\Gamma (\gamma +1)}[{\mathfrak {t}}_{1\mathfrak {j}}^{\star }+{\mathfrak {t}}_{3\mathfrak {j}}^{\star }]||\texttt {x}^{\prime }({\mathfrak {t}})-\texttt {x}({\mathfrak {t}})||\\&\qquad +\frac{{\mathfrak {t}}^{\gamma \mu }}{\mu ^{\gamma }\Gamma (\gamma +1)}[{\mathfrak {t}}_{2\mathfrak {j}}^{\star }+{\mathfrak {t}}_{4\mathfrak {j}}^{\star }]||\Theta ^{\prime }({\mathfrak {t}})-\Theta ({\mathfrak {t}})||+\frac{({\mathfrak {t}}^{\mu }-{\tau }^{\mu })^{\gamma }}{\mu ^{\gamma }\Gamma (\gamma +1)}[{\mathfrak {t}}_{2\mathfrak {j}}^{\star }+{\mathfrak {t}}_{4\mathfrak {j}}^{\star }]||\Theta ^{\prime }({\mathfrak {t}})-\Theta ({\mathfrak {t}})||\\&\qquad +\frac{({\mathfrak {t}}^{\mu }-{\tau }^{\mu })^{\gamma }}{\mu ^{\gamma }\Gamma (\gamma +1)}[{\mathfrak {t}}_{2\mathfrak {j}}^{\star }+{\mathfrak {t}}_{4\mathfrak {j}}^{\star }]||\texttt {y}^{\prime }({\mathfrak {t}})-\texttt {y}({\mathfrak {t}})|| \end{aligned}$$

2.10 $$\begin{aligned} ||\texttt {y}^{\prime }({\mathfrak {t}})-\texttt {y}({\mathfrak {t}})||&={\sum }_{\mathfrak {j}=1}^{n}{\sup }_{{\mathfrak {t}}}\{{e }^{\mathfrak {i}({\mathfrak {t}}-1)}|\texttt {y}^{\prime }_{\mathfrak {j}}({\mathfrak {t}})-\texttt {y}_{\mathfrak {j}}({\mathfrak {t}})|\}\nonumber \\&\precsim \Big [c_{\max }+||\mathfrak {q}_{2}^{\star }+\mathfrak {q}_{4}^{\star }||+||{\mathfrak {t}}_{2}^{\star }+{\mathfrak {t}}_{4}^{\star }||\Big ]||\texttt {y}^{\prime }({\mathfrak {t}})-\texttt {y}({\mathfrak {t}})||\nonumber \\&\qquad +\Big [||\mathfrak {q}_{1}^{\star }+|\mathfrak {q}_{3}^{\star }||+||{\mathfrak {t}}_{1}^{\star }+{\mathfrak {t}}_{3}^{\star }||\Big ]||\texttt {x}^{\prime }({\mathfrak {t}})-\texttt {x}({\mathfrak {t}})||\nonumber \\&\qquad +\Big [1+||{\mathfrak {t}}_{2}^{\star }+{\mathfrak {t}}_{4}^{\star }||+||{\mathfrak {t}}_{2}^{\star }+{\mathfrak {t}}_{4}^{\star }||\Big ]||\Theta ^{\prime }({\mathfrak {t}})-\Theta ({\mathfrak {t}})||\nonumber \\&\qquad +\Big [||{\mathfrak {t}}_{1}^{\star }+{\mathfrak {t}}_{3}^{\star }||+||{\mathfrak {t}}_{1}^{\star }+{\mathfrak {t}}_{3}^{\star }||\Big ]||\Psi ^{\prime }({\mathfrak {t}})-\Psi ({\mathfrak {t}})|| \end{aligned}$$

From Eq. (2.10), one can easily obtain that

2.11 $$\begin{aligned} ||\texttt {y}^{\prime }({\mathfrak {t}})-\texttt {y}({\mathfrak {t}})||&\precsim \frac{||\mathfrak {q}_{1}^{\star }+|\mathfrak {q}_{3}^{\star }||+||{\mathfrak {t}}_{1}^{\star }+{\mathfrak {t}}_{3}^{\star }||}{1-\Big [c_{\max }+||\mathfrak {q}_{2}^{\star }+\mathfrak {q}_{4}^{\star }||+||{\mathfrak {t}}_{2}^{\star }+{\mathfrak {t}}_{4}^{\star }||\Big ]}||\texttt {x}^{\prime }({\mathfrak {t}})-\texttt {x}({\mathfrak {t}})||\nonumber \\&\ \ \ \ +\frac{||1+2||{\mathfrak {t}}_{2}^{\star }+{\mathfrak {t}}_{4}^{\star }||}{1-\Big [c_{\max }+||\mathfrak {q}_{2}^{\star }+\mathfrak {q}_{4}^{\star }||+||{\mathfrak {t}}_{2}^{\star }+{\mathfrak {t}}_{4}^{\star }||\Big ]}||\Theta ^{\prime }({\mathfrak {t}})-\Theta ({\mathfrak {t}})||\nonumber \\&\ \ \ \ +\frac{||2||{\mathfrak {t}}_{1}^{\star }+{\mathfrak {t}}_{3}^{\star }||}{1-\Big [c_{\max }+||\mathfrak {q}_{2}^{\star }+\mathfrak {q}_{4}^{\star }||+||{\mathfrak {t}}_{2}^{\star }+{\mathfrak {t}}_{4}^{\star }||\Big ]}||\Psi ^{\prime }({\mathfrak {t}})-\Psi ({\mathfrak {t}})|| \end{aligned}$$

From (2.8) and (2.11), the following can be expressed in the formulation:

2.12 $$\begin{aligned}{} & {} ||\texttt {x}^{\prime }({\mathfrak {t}})-\texttt {x}({\mathfrak {t}})||\precsim \frac{1}{\mathscr {U}_{1}}\{\mathscr {U}_{2}||\texttt {y}^{\prime }({\mathfrak {t}})-\texttt {y}({\mathfrak {t}})||+\mathscr {U}_{3}||\Psi ^{\prime }({\mathfrak {t}})-\Psi ({\mathfrak {t}})||+\mathscr {U}_{4}||\Theta ^{\prime }({\mathfrak {t}})-\Theta ({\mathfrak {t}})||\} \end{aligned}$$

2.13 $$\begin{aligned}{} & {} ||\texttt {y}^{\prime }({\mathfrak {t}})-\texttt {y}({\mathfrak {t}})||\precsim \frac{1}{\mathscr {V}_{1}}\{\mathscr {V}_{2}||\texttt {x}^{\prime }({\mathfrak {t}})-\texttt {x}({\mathfrak {t}})||+\mathscr {V}_{3}||\Theta ^{\prime }({\mathfrak {t}})-\Theta ({\mathfrak {t}})||+\mathscr {V}_{4}||\Psi ^{\prime }({\mathfrak {t}})-\Psi ({\mathfrak {t}})||\} \end{aligned}$$

where,

$$\begin{aligned}{} & {} \mathscr {U}_{1}=1-\Big [c_{\max }+||\mathfrak {a}_{1}^{\star }+\mathfrak {a}_{3}^{\star }||+||\mathfrak {b}_{1}^{\star }+\mathfrak {b}_{3}^{\star }||\Big ];\\{} & {} \mathscr {U}_{2}=||\mathfrak {a}_{2}^{\star }+\mathfrak {a}_{4}^{\star }||+||\mathfrak {b}_{2}^{\star }+\mathfrak {b}_{4}^{\star }||;\\{} & {} \mathscr {U}_{3}=1+2||\mathfrak {b}_{1}^{\star }+\mathfrak {b}_{3}^{\star }||;\\{} & {} \mathscr {U}_{4}=2||\mathfrak {b}_{2}^{\star }+\mathfrak {b}_{4}^{\star }||;\\{} & {} \mathscr {U}_{1}=1-\Big [c_{\max }+||\mathfrak {q}_{2}^{\star }+\mathfrak {q}_{4}^{\star }||+||{\mathfrak {t}}_{2}^{\star }+{\mathfrak {t}}_{4}^{\star }||\Big ];\\{} & {} \mathscr {U}_{2}=||\mathfrak {q}_{1}^{\star }+\mathfrak {q}_{3}^{\star }||+||{\mathfrak {t}}_{1}^{\star }+{\mathfrak {t}}_{3}^{\star }||;\\{} & {} \mathscr {U}_{3}=1+2||{\mathfrak {t}}_{2}^{\star }+{\mathfrak {t}}_{4}^{\star }||;\\{} & {} \mathscr {U}_{4}=2||{\mathfrak {t}}_{1}^{\star }+{\mathfrak {t}}_{3}^{\star }||. \end{aligned}$$

Equations (2.12) and (2.13) can be written in the following form:

2.14 $$\begin{aligned}{} & {} ||\texttt {x}^{\prime }({\mathfrak {t}})-\texttt {x}({\mathfrak {t}})||\precsim \frac{\mathscr {U}_{2}}{\mathscr {U}_{1}}||\texttt {y}^{\prime }({\mathfrak {t}})-\texttt {y}({\mathfrak {t}})||+\frac{\mathscr {U}_{3}}{\mathscr {U}_{1}}||\Psi ^{\prime }({\mathfrak {t}})-\Psi ({\mathfrak {t}})||+\frac{\mathscr {U}_{4}}{\mathscr {U}_{1}}||\Theta ^{\prime }({\mathfrak {t}})-\Theta ({\mathfrak {t}})|| \end{aligned}$$

2.15 $$\begin{aligned}{} & {} ||\texttt {y}^{\prime }({\mathfrak {t}})-\texttt {y}({\mathfrak {t}})||\precsim \frac{\mathscr {V}_{2}}{\mathscr {V}_{1}}||\texttt {x}^{\prime }({\mathfrak {t}})-\texttt {x}({\mathfrak {t}})||+\frac{\mathscr {V}_{3}}{\mathscr {V}_{1}}||\Theta ^{\prime }({\mathfrak {t}})-\Theta ({\mathfrak {t}})||+\frac{\mathscr {V}_{4}}{\mathscr {V}_{1}}||\Psi ^{\prime }({\mathfrak {t}})-\Psi ({\mathfrak {t}})|| \end{aligned}$$

By substituting (2.15) in (2.14), we get,

$$\begin{aligned} ||\texttt {x}^{\prime }({\mathfrak {t}})-\texttt {x}({\mathfrak {t}})||&\precsim \frac{\mathscr {U}_{2}}{\mathscr {U}_{1}}\biggl (\frac{\mathscr {V}_{2}}{\mathscr {V}_{1}}||\texttt {x}^{\prime }({\mathfrak {t}})-\texttt {x}({\mathfrak {t}})||+\frac{\mathscr {V}_{3}}{\mathscr {V}_{1}}||\Theta ^{\prime }({\mathfrak {t}})-\Theta ({\mathfrak {t}})||+\frac{\mathscr {V}_{4}}{\mathscr {V}_{1}}||\Psi ^{\prime }({\mathfrak {t}})-\Psi ({\mathfrak {t}})|| \biggr )\\&\quad +\frac{\mathscr {U}_{3}}{\mathscr {U}_{1}}||\Psi ^{\prime }({\mathfrak {t}})-\Psi ({\mathfrak {t}})||+\frac{\mathscr {U}_{4}}{\mathscr {U}_{1}}||\Theta ^{\prime }({\mathfrak {t}})-\Theta ({\mathfrak {t}})||\\&\quad \precsim \frac{\mathscr {U}_{2}\mathscr {V}_{2}}{\mathscr {U}_{1}\mathscr {V}_{1}}||\texttt {x}^{\prime }({\mathfrak {t}})-\texttt {x}({\mathfrak {t}})||+\biggl [\frac{\mathscr {U}_{2}\mathscr {V}_{3}}{\mathscr {U}_{1}\mathscr {V}_{1}}+\frac{\mathscr {U}_{4}}{\mathscr {U}_{1}}\biggr ]||\Theta ^{\prime }({\mathfrak {t}})-\Theta ({\mathfrak {t}})||+\biggl [\frac{\mathscr {U}_{2}\mathscr {V}_{4}}{\mathscr {U}_{1}\mathscr {V}_{1}}+\frac{\mathscr {U}_{3}}{\mathscr {U}_{1}}\biggr ]||\Psi ^{\prime }({\mathfrak {t}})-\Psi ({\mathfrak {t}})|| \end{aligned}$$

This gives,

2.16 $$\begin{aligned} ||\texttt {x}^{\prime }({\mathfrak {t}})-\texttt 
{x}({\mathfrak {t}})||\precsim \Biggl (\frac{\frac{\mathscr {U}_{2}\mathscr {V}_{3}}{\mathscr {U}_{1}\mathscr {V}_{1}}+\frac{\mathscr {U}_{4}}{\mathscr {U}_{1}}}{1-\frac{\mathscr {U}_{2}\mathscr {V}_{2}}{\mathscr {U}_{1}\mathscr {V}_{1}}}\Biggr )||\Theta ^{\prime }({\mathfrak {t}})-\Theta ({\mathfrak {t}})||+\Biggl (\frac{\frac{\mathscr {U}_{2}\mathscr {V}_{4}}{\mathscr {U}_{1}\mathscr {V}_{1}}+\frac{\mathscr {U}_{3}}{\mathscr {U}_{1}}}{1-\frac{\mathscr {U}_{2}\mathscr {V}_{2}}{\mathscr {U}_{1}\mathscr {V}_{1}}}\Biggr )||\Psi ^{\prime }({\mathfrak {t}})-\Psi ({\mathfrak {t}})|| \end{aligned}$$

Similarly, by substituting (2.14) in (2.15), we get,

$$\begin{aligned} ||\texttt {y}^{\prime }({\mathfrak {t}})-\texttt {y}({\mathfrak {t}})||&\precsim \frac{\mathscr {V}_{2}}{\mathscr {V}_{1}}\biggl (\frac{\mathscr {U}_{2}}{\mathscr {U}_{1}}||\texttt {y}^{\prime }({\mathfrak {t}})-\texttt {y}({\mathfrak {t}})||+\frac{\mathscr {U}_{3}}{\mathscr {U}_{1}}||\Psi ^{\prime }({\mathfrak {t}})-\Psi ({\mathfrak {t}})||+\frac{\mathscr {U}_{4}}{\mathscr {U}_{1}}||\Theta ^{\prime }({\mathfrak {t}})-\Theta ({\mathfrak {t}})|| \biggr )\\&\quad +\frac{\mathscr {V}_{3}}{\mathscr {V}_{1}}||\Theta ^{\prime }({\mathfrak {t}})-\Theta ({\mathfrak {t}})||+\frac{\mathscr {V}_{4}}{\mathscr {V}_{1}}||\Psi ^{\prime }({\mathfrak {t}})-\Psi ({\mathfrak {t}})||\\&\precsim \frac{\mathscr {V}_{2}\mathscr {U}_{2}}{\mathscr {V}_{1}\mathscr {U}_{1}}||\texttt {y}^{\prime }({\mathfrak {t}})-\texttt {y}({\mathfrak {t}})||+\biggl [\frac{\mathscr {V}_{2}\mathscr {U}_{3}}{\mathscr {U}_{1}\mathscr {V}_{1}}+\frac{\mathscr {V}_{4}}{\mathscr {V}_{1}}\biggr ]||\Psi ^{\prime }({\mathfrak {t}})-\Psi ({\mathfrak {t}})||+\biggl [\frac{\mathscr {V}_{2}\mathscr {U}_{4}}{\mathscr {U}_{1}\mathscr {V}_{1}}+\frac{\mathscr {V}_{3}}{\mathscr {V}_{1}}\biggr ]||\Theta ^{\prime }({\mathfrak {t}})-\Theta ({\mathfrak {t}})|| \end{aligned}$$

This gives,

2.17 $$\begin{aligned} ||\texttt {y}^{\prime }({\mathfrak {t}})-\texttt {y}({\mathfrak {t}})||\precsim \Biggl (\frac{\frac{\mathscr {V}_{2}\mathscr {U}_{3}}{\mathscr {V}_{1}\mathscr {U}_{1}}+\frac{\mathscr {V}_{4}}{\mathscr {V}_{1}}}{1-\frac{\mathscr {V}_{2}\mathscr {U}_{2}}{\mathscr {V}_{1}\mathscr {U}_{1}}}\Biggr )||\Psi ^{\prime }({\mathfrak {t}})-\Psi ({\mathfrak {t}})||+\Biggl (\frac{\frac{\mathscr {V}_{2}\mathscr {U}_{4}}{\mathscr {V}_{1}\mathscr {U}_{1}}+\frac{\mathscr {V}_{3}}{\mathscr {V}_{1}}}{1-\frac{\mathscr {V}_{2}\mathscr {U}_{2}}{\mathscr {V}_{1}\mathscr {U}_{1}}}\Biggr )||\Theta ^{\prime }({\mathfrak {t}})-\Theta ({\mathfrak {t}})|| \end{aligned}$$

If we consider

$$\begin{aligned}{} & {} ||\Psi ^{\prime }({\mathfrak {t}})-\Psi ({\mathfrak {t}})||\precsim \frac{\eta _{1}}{2\Biggl (\frac{\frac{\mathscr {U}_{2}\mathscr {V}_{4}}{\mathscr {U}_{1}\mathscr {V}_{1}}+\frac{\mathscr {U}_{3}}{\mathscr {U}_{1}}}{1-\frac{\mathscr {U}_{2}\mathscr {V}_{2}}{\mathscr {U}_{1}\mathscr {V}_{1}}}\Biggr )}=\frac{\eta _{1}}{2\vartheta _{1}}\\{} & {} ||\Theta ^{\prime }({\mathfrak {t}})-\Theta ({\mathfrak {t}})||\precsim \frac{\eta _{1}}{2\Biggl (\frac{\frac{\mathscr {U}_{2}\mathscr {V}_{3}}{\mathscr {U}_{1}\mathscr {V}_{1}}+\frac{\mathscr {U}_{4}}{\mathscr {U}_{1}}}{1-\frac{\mathscr {U}_{2}\mathscr {V}_{2}}{\mathscr {U}_{1}\mathscr {V}_{1}}}\Biggr )}=\frac{\eta _{1}}{2\vartheta _{2}} \end{aligned}$$

where,

$$\begin{aligned} \vartheta _{1}=\Biggl (\frac{\frac{\mathscr {U}_{2}\mathscr {V}_{4}}{\mathscr {U}_{1}\mathscr {V}_{1}}+\frac{\mathscr {U}_{3}}{\mathscr {U}_{1}}}{1-\frac{\mathscr {U}_{2}\mathscr {V}_{2}}{\mathscr {U}_{1}\mathscr {V}_{1}}}\Biggr ) \ \ \text {and} \ \ \vartheta _{2}=\Biggl (\frac{\frac{\mathscr {U}_{2}\mathscr {V}_{3}}{\mathscr {U}_{1}\mathscr {V}_{1}}+\frac{\mathscr {U}_{4}}{\mathscr {U}_{1}}}{1-\frac{\mathscr {U}_{2}\mathscr {V}_{2}}{\mathscr {U}_{1}\mathscr {V}_{1}}}\Biggr ). \end{aligned}$$

Therefore, Eq. (2.16) becomes

2.18 $$\begin{aligned} ||\texttt {x}^{\prime }({\mathfrak {t}})-\texttt {x}({\mathfrak {t}})||\precsim \eta _{1}. \end{aligned}$$

Similarly, if we consider

$$\begin{aligned}{} & {} ||\Psi ^{\prime }({\mathfrak {t}})-\Psi ({\mathfrak {t}})||\precsim \frac{\eta _{2}}{2\Biggl (\frac{\frac{\mathscr {V}_{2}\mathscr {U}_{3}}{\mathscr {V}_{1}\mathscr {U}_{1}}+\frac{\mathscr {V}_{4}}{\mathscr {V}_{1}}}{1-\frac{\mathscr {V}_{2}\mathscr {U}_{2}}{\mathscr {V}_{1}\mathscr {U}_{1}}}\Biggr )}=\frac{\eta _{2}}{2\vartheta _{3}}\\{} & {} ||\Theta ^{\prime }({\mathfrak {t}})-\Theta ({\mathfrak {t}})||\precsim \frac{\eta _{2}}{2\Biggl (\frac{\frac{\mathscr {V}_{2}\mathscr {U}_{4}}{\mathscr {V}_{1}\mathscr {U}_{1}}+\frac{\mathscr {V}_{3}}{\mathscr {V}_{1}}}{1-\frac{\mathscr {V}_{2}\mathscr {U}_{2}}{\mathscr {V}_{1}\mathscr {U}_{1}}}\Biggr )}=\frac{\eta _{2}}{2\vartheta _{4}} \end{aligned}$$

where,

$$\begin{aligned} \vartheta _{3}=\Biggl (\frac{\frac{\mathscr {V}_{2}\mathscr {U}_{3}}{\mathscr {V}_{1}\mathscr {U}_{1}}+\frac{\mathscr {V}_{4}}{\mathscr {V}_{1}}}{1-\frac{\mathscr {V}_{2}\mathscr {U}_{2}}{\mathscr {V}_{1}\mathscr {U}_{1}}}\Biggr ) \ \ \text {and} \ \ \vartheta _{4}=\Biggl (\frac{\frac{\mathscr {V}_{2}\mathscr {U}_{4}}{\mathscr {V}_{1}\mathscr {U}_{1}}+\frac{\mathscr {V}_{3}}{\mathscr {V}_{1}}}{1-\frac{\mathscr {V}_{2}\mathscr {U}_{2}}{\mathscr {V}_{1}\mathscr {U}_{1}}}\Biggr ). \end{aligned}$$

Therefore, Eq. (2.17) becomes

2.19 $$\begin{aligned} ||\texttt {y}^{\prime }({\mathfrak {t}})-\texttt {y}({\mathfrak {t}})||\precsim \eta _{2}. \end{aligned}$$

From Eqs. (2.18) and (2.19), we say that for all $\eta =\max \{\eta _{1},\eta _{2}\}>0$ then there exists a $\vartheta =\frac{\eta }{\max \{\eta _{5},\eta _{6}\}>0}$, where $\eta _{5}=\max \{\eta _{1},\eta _{3}\}$, $\eta _{6}=\max \{\eta _{2},\eta _{4}\}$, such that $||{z}^{\prime }({\mathfrak {t}})-{z}({\mathfrak {t}})||\precsim \eta$ when $||\varkappa ({\mathfrak {t}})-\lambda ({\mathfrak {t}})||<\vartheta$. It demonstrates the uniform stability of the solution ${z}({\mathfrak {t}})$. $\square$

### Existence of unique equilibrium point

Let $\mathscr {M}=\mathcal {C}([-{\tau },0],\mathbb {C}^{n})$, where $\mathcal {C}$ is the set of all continuous functions defined on $[-{\tau },0]$.

Define the mapping ${d}:\mathscr {M}\times \mathscr {M}\rightarrow \mathbb {C}$ as

$$\begin{aligned} {d}({z}({\mathfrak {t}}),{z}^{\prime }({\mathfrak {t}}))=|{z}({\mathfrak {t}})-{z}^{\prime }({\mathfrak {t}})|+\mathfrak {i}|{z}({\mathfrak {t}})-{z}^{\prime }({\mathfrak {t}})|. \end{aligned}$$

Clearly $(\mathscr {M},{d})$ is a complete complex-valued metric space. Define $\mathscr {W}:\mathscr {M}\rightarrow \mathscr {M}$ as follows:

$$\begin{aligned} \mathscr {W}\rho (\kappa )={\sum }_{\mathfrak {j}=1}^{n}\mathfrak {s}_{\rho \mathfrak {j}}\varphi _{\mathfrak {j}}\bigg (\frac{\kappa _{\mathfrak {j}}}{c_{\mathfrak {j}}}\bigg )+{\sum }_{\mathfrak {j}=1}^{n}\mathfrak {p}_{\rho \mathfrak {j}}\varphi _{\mathfrak {j}}\bigg (\frac{\kappa _{\mathfrak {j}}}{c_{\mathfrak {j}}}\bigg )+\Upsilon _{\rho }, \end{aligned}$$

where $\rho =1,2,3\ldots n$ and, for $\kappa =(\kappa _{1},\kappa _{2},\kappa _{3},\ldots \kappa _{n})^{\mathbb {T}}.$

#### Theorem 2.2

There exist a unique equilibrium point for the system (1.1) if the following conditions satisfied, which is uniformly stable.

(1).: Assumption $$(\mathcal {A})$$, $$(\mathcal {B})$$, and $$(\mathcal {C})$$;
(2).: $$||\mathfrak {a}^{\star }||+||\mathfrak {b}^{\star }||<c_{\min }$$.

#### Proof

Consider the two vectors $\kappa =(\kappa _{1},\kappa _{2},\kappa _{3},\ldots \kappa _{n})^{\mathbb {T}}$ and $\upsilon =(\upsilon _{1},\upsilon _{2},\upsilon _{3},\ldots \upsilon _{n})^{\mathbb {T}}$, where $\kappa \ne \upsilon$. Now consider,

$$\begin{aligned}&|\mathscr {W}\kappa -\mathscr {W}\upsilon |+\mathfrak {i}|\mathscr {W}\kappa -\mathscr {W}\upsilon |\\&={\sum }_{\ell =1}^{n}|\mathscr {W}_{\ell }(\kappa )-\mathscr {W}_{\ell }(\upsilon )| +\mathfrak {i}{\sum }_{\ell =1}^{n}|\mathscr {W}_{\ell }(\kappa )-\mathscr {W}_{\ell }(\upsilon )|\\&={\sum }_{\ell =1}^{n}\bigg |{\sum }_{\mathfrak {j}=1}^{n}\mathfrak {s}_{\ell \mathfrak {j}}\varphi _{\mathfrak {j}}\bigg (\frac{\kappa _{\mathfrak {j}}}{c_{\mathfrak {j}}}\bigg )+{\sum }_{\mathfrak {j}=1}^{n}\mathfrak {p}_{\ell \mathfrak {j}}\varphi _{\mathfrak {j}}\bigg (\frac{\kappa _{\mathfrak {j}}}{c_{\mathfrak {j}}}\bigg )-{\sum }_{\mathfrak {j}=1}^{n}\mathfrak {s}_{\ell \mathfrak {j}}\varphi _{\mathfrak {j}}\bigg (\frac{\upsilon _{\mathfrak {j}}}{c_{\mathfrak {j}}}\bigg )-{\sum }_{\mathfrak {j}=1}^{n}\mathfrak {p}_{\ell \mathfrak {j}}\varphi _{\mathfrak {j}}\bigg (\frac{\upsilon _{\mathfrak {j}}}{c_{\mathfrak {j}}}\bigg )\bigg |\\&\quad +\mathfrak {i}{\sum }_{\ell =1}^{n}\bigg |{\sum }_{\mathfrak {j}=1}^{n}\mathfrak {s}_{\ell \mathfrak {j}}\varphi _{\mathfrak {j}}\bigg (\frac{\kappa _{\mathfrak {j}}}{c_{\mathfrak {j}}}\bigg )+{\sum }_{\mathfrak {j}=1}^{n}\mathfrak {p}_{\ell \mathfrak {j}}\varphi _{\mathfrak {j}}\bigg (\frac{\kappa _{\mathfrak {j}}}{c_{\mathfrak {j}}}\bigg )-{\sum }_{\mathfrak {j}=1}^{n}\mathfrak {s}_{\ell \mathfrak {j}}\varphi _{\mathfrak {j}}\bigg (\frac{\upsilon _{\mathfrak {j}}}{c_{\mathfrak {j}}}\bigg )-{\sum }_{\mathfrak {j}=1}^{n}\mathfrak {p}_{\ell \mathfrak {j}}\varphi _{\mathfrak {j}}\bigg (\frac{\upsilon _{\mathfrak {j}}}{c_{\mathfrak {j}}}\bigg )\bigg |\\&={\sum }_{\ell =1}^{n}\bigg |{\sum }_{\mathfrak {j}=1}^{n}\mathfrak {s}_{\ell \mathfrak {j}}\bigg [\varphi _{\mathfrak {j}}\bigg (\frac{\kappa _{\mathfrak {j}}}{c_{\mathfrak {j}}}\bigg )-\varphi _{\mathfrak {j}}\bigg (\frac{\upsilon _{\mathfrak {j}}}{c_{\mathfrak {j}}}\bigg )\bigg ]+{\sum }_{\mathfrak {j}=1}^{n}\mathfrak {p}_{\ell \mathfrak {j}}\bigg [\varphi _{\mathfrak {j}}\bigg (\frac{\kappa _{\mathfrak {j}}}{c_{\mathfrak {j}}}\bigg )-\varphi _{\mathfrak {j}}\bigg (\frac{\upsilon _{\mathfrak {j}}}{c_{\mathfrak {j}}}\bigg )\bigg ]\bigg |\\&\quad +\mathfrak {i}{\sum }_{\ell =1}^{n}\bigg |{\sum }_{\mathfrak {j}=1}^{n}\mathfrak {s}_{\ell \mathfrak {j}}\bigg [\varphi _{\mathfrak {j}}\bigg (\frac{\kappa _{\mathfrak {j}}}{c_{\mathfrak {j}}}\bigg )-\varphi _{\mathfrak {j}}\bigg (\frac{\upsilon _{\mathfrak {j}}}{c_{\mathfrak {j}}}\bigg )\bigg ]+{\sum }_{\mathfrak {j}=1}^{n}\mathfrak {p}_{\ell \mathfrak {j}}\bigg [\varphi _{\mathfrak {j}}\bigg (\frac{\kappa _{\mathfrak {j}}}{c_{\mathfrak {j}}}\bigg )-\varphi _{\mathfrak {j}}\bigg (\frac{\upsilon _{\mathfrak {j}}}{c_{\mathfrak {j}}}\bigg )\bigg ]\bigg |\\&\precsim {\sum }_{\ell =1}^{n} \bigg [{\sum }_{\mathfrak {j}=1}^{n}\frac{\mathfrak {s}_{\ell \mathfrak {j}}\theta _{\mathfrak {j}}}{c_{\mathfrak {j}}}|\kappa _{\mathfrak {j}}-\upsilon _{\mathfrak {j}}|+{\sum }_{\mathfrak {j}=1}^{n}\frac{\mathfrak {p}_{\ell \mathfrak {j}}\theta _{\mathfrak {j}}}{c_{\mathfrak {j}}}|\kappa _{\mathfrak {j}}-\upsilon _{\mathfrak {j}}|\bigg ]\\&\quad +\mathfrak {i}{\sum }_{\ell =1}^{n} \bigg [{\sum }_{\mathfrak {j}=1}^{n}\frac{\mathfrak {s}_{\ell \mathfrak {j}}\theta _{\mathfrak {j}}}{c_{\mathfrak {j}}}|\kappa _{\mathfrak {j}}-\upsilon _{\mathfrak {j}}|+{\sum }_{\mathfrak {j}=1}^{n}\frac{\mathfrak {p}_{\ell \mathfrak {j}}\theta _{\mathfrak {j}}}{c_{\mathfrak {j}}}|\kappa _{\mathfrak {j}}-\upsilon _{\mathfrak {j}}|\bigg ]\\&\precsim {\sum }_{\ell =1}^{n}\bigg ({\sum }_{\mathfrak {j}=1}^{n}\frac{\mathfrak {s}_{\ell \mathfrak {j}}\theta _{\mathfrak {j}}+\mathfrak {p}_{\ell \mathfrak {j}}\theta _{\mathfrak {j}}}{c_{\mathfrak {j}}}|\kappa _{\mathfrak {j}}-\upsilon _{\mathfrak {j}}|\bigg )+\mathfrak {i}{\sum }_{\ell =1}^{n}\bigg ({\sum }_{\mathfrak {j}=1}^{n}\frac{\mathfrak {s}_{\ell \mathfrak {j}}\theta _{\mathfrak {j}}+\mathfrak {p}_{\ell \mathfrak {j}}\theta _{\mathfrak {j}}}{c_{\mathfrak {j}}}|\kappa _{\mathfrak {j}}-\upsilon _{\mathfrak {j}}|\bigg )\\&\precsim \bigg (\frac{||\mathfrak {a}^{\star }||+||\mathfrak {b}^{\star }||}{c_{\min }}\bigg [{\sum }_{\mathfrak {j}=1}^{n}|\kappa _{\mathfrak {j}}-\upsilon _{\mathfrak {j}}|\bigg ]\bigg )+\mathfrak {i}\bigg (\frac{||\mathfrak {a}^{\star }||+||\mathfrak {b}^{\star }||}{c_{\min }}\bigg [{\sum }_{\mathfrak {j}=1}^{n}|\kappa _{\mathfrak {j}}-\upsilon _{\mathfrak {j}}|\bigg ]\bigg )\\&\precsim \frac{||\mathfrak {a}^{\star }||+||\mathfrak {b}^{\star }||}{c_{\min }}\big [|\kappa -\upsilon |+\mathfrak {i}|\kappa -\upsilon |\big ]\\&=\delta {d}(\kappa ,\upsilon ), \end{aligned}$$

which yields ${d}(\mathscr {W}\kappa ,\mathscr {W}\upsilon )\precsim \delta {d}(\kappa ,\upsilon )$, where $\delta =\frac{||\mathfrak {a}^{\star }||+||\mathfrak {b}^{\star }||}{c_{\min }}$.

Hence $\mathscr {W}$ is a contractive mapping on $\mathbb {C}^{n}$. Hence by using Theorem.1.2, there will be a unique fixed point $\kappa ^{\star }\in \mathbb {C}^{n}$ in such a way that $\mathscr {W}(\kappa ^{\star })=\kappa ^{\star }$. Therefore,

$$\begin{aligned} \kappa ^{\star }_{\mathfrak {j}}={\sum }_{\ell =1}^{n}\mathfrak {s}_{\mathfrak {j}\ell }\varphi _{\ell }\bigg (\frac{\kappa _{\mathfrak {j}}^{\star }}{c_{\mathfrak {j}}}\bigg )+{\sum }_{\ell =1}^{n}\mathfrak {p}_{\mathfrak {j}\ell }\varphi _{\ell }\bigg (\frac{\upsilon _{\mathfrak {j}}^{\star }}{c_{\mathfrak {j}}}\bigg )+\Upsilon _{\mathfrak {j}}, \ \mathfrak {j}=1,2,3\ldots n \end{aligned}$$

Consider $$c_{\mathfrak {j}}{z}_{\mathfrak {j}}^{\star }=\kappa _{\mathfrak {j}}^{\star }, \ \mathfrak {j}=1,2,3\ldots n$$ then

$$\begin{aligned} -c_{\mathfrak {j}}{z}_{\mathfrak {j}}^{\star }+{\sum }_{\ell =1}^{n}\mathfrak {s}_{\mathfrak {j}\ell }\varphi _{\ell }\big ({z}_{\ell }^{\star }\big )+{\sum }_{\ell =1}^{n}\mathfrak {p}_{\mathfrak {j}\ell }\varphi _{\ell }\big ({z}_{\ell }^{\star }\big )+\Upsilon _{\mathfrak {j}}=0, \ \mathfrak {j}=1,2,3\ldots n \end{aligned}$$

Thus (1.1) has a unique equillibrium point ${z}^{\star }$. Moreover ${z}^{\star }$ is uniformly stable followed by using above Theorem.2.1. $\square$

## Numerical simulations

In this part, two numerical examples are provided to illustrate and validate the presented theoretical results.

### Example 3.1

Consider a fractional-order CVNN with time delays is presented as follows:

3.1 $$\begin{aligned} ^{C }\mathscr {D}_{0}^{\gamma ,\mu }{z}_{k}({\mathfrak {t}})=-c_{k}{z}_{k}({\mathfrak {t}})+{\sum }_{\mathfrak {j}=1}^{2}\mathfrak {s}_{k\mathfrak {j}}\varphi _{\mathfrak {j}}({z}_{\mathfrak {j}}({\mathfrak {t}}))+{\sum }_{\mathfrak {j}=1}^{2}\mathfrak {p}_{k\mathfrak {j}}\varphi _{\mathfrak {j}}({z}_{\mathfrak {j}}({\mathfrak {t}}-{\tau }))+\Upsilon _{k}; \ k=1,2. \end{aligned}$$

**Figure 1:**
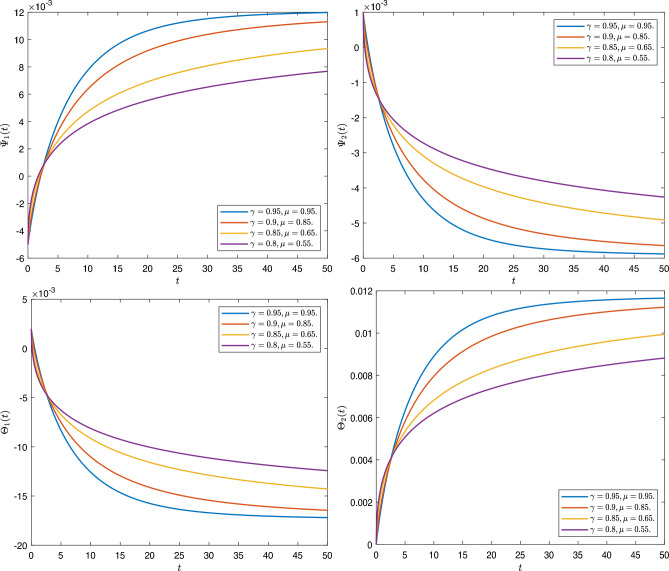
The trajectories of the real and imaginary parts of ${z}_{k},\,k=1,\,2$ for Example 3.1 with the initial condition $\vartheta _1$ and various values of $\gamma$ and $\mu .$.

**Figure 2:**
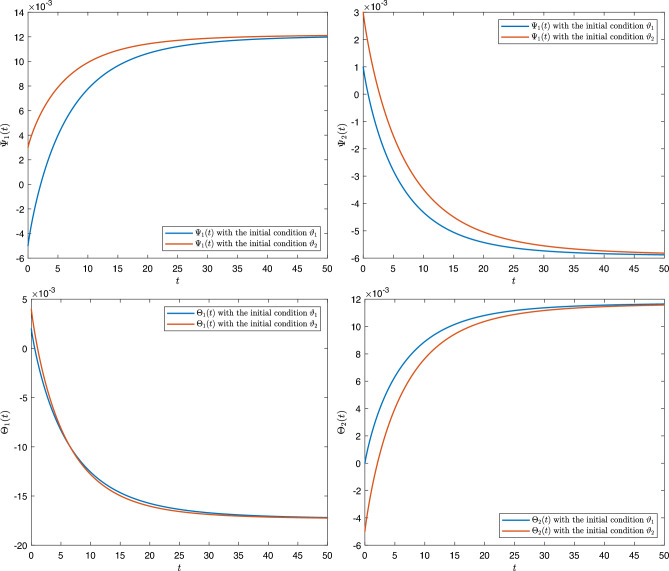
The trajectories of the real and imaginary parts of ${z}_{k},\,k=1,\,2$ for Example 3.1 with the initial conditions $\vartheta _1$ and $\vartheta _2$ at $\gamma = \mu = 0.95.$.

Let us assume that the parameters of the given system are chosen as ${\tau }=1,\,c_{1}=0.15,\,c_{2}=0.14,\,\mathfrak {s}_{11}=-0.03-0.04\mathfrak {i},\,\mathfrak {s}_{12}=0.05-0.04\mathfrak {i},\,\mathfrak {s}_{21}=0.04+0.007\mathfrak {i},\,\mathfrak {s}_{22}=-0.006+0.02\mathfrak {i},\,\mathfrak {p}_{11}=0.03-0.08\mathfrak {i},\, \mathfrak {p}_{12}=0.2-0.02\mathfrak {i},\,\mathfrak {p}_{21}=0.02+0.03\mathfrak {i},\,\mathfrak {p}_{22}=-0.02+0.06\mathfrak {i},\,\Upsilon _{1}=0.002-0.003\mathfrak {i},\,\Upsilon _{2}=-0.001+0.002\mathfrak {i},{z}_{k}({\mathfrak {t}})= \Psi _{k}({\mathfrak {t}})+\mathfrak {i}\Theta _{k}({\mathfrak {t}}),\,\varphi _{k}({z}_{k})=0.2\tanh (\Psi _{k})+0.2\tanh (\Theta _{k})\mathfrak {i},$ and the function in terms of the delay term is defined as $\varphi _{k}({z}_{k})=0.1\tanh (\Psi _{k})+0.1\tanh (\Theta _{k})\mathfrak {i},\,k=1,\,2.$ The presented system was solved using various values for $\gamma ,\,\mu$ as well as two initial conditions $$\vartheta _1= \begin{bmatrix} -0.005+0.002\mathfrak {i}\\ 0.001 \\ \end{bmatrix},$$ and $$\vartheta _2= \begin{bmatrix} 0.003+0.004\mathfrak {i}\\ 0.003-0.005\mathfrak {i} \\ \end{bmatrix}.$$ Figure 1, shows the time trajectories of system (3.1) with various values of $\gamma$ and $\mu$ at the initial condition $\vartheta _1.$ It is evident that the solutions, for all given values of $\gamma$ and $\mu ,$ converge to an equilibrium point that is consistent with the obtained theoretical results. Table 1, displays the equilibrium points at different values of $\gamma$ and $\mu$ for ${z}_{k}({\mathfrak {t}}),\,k=1,\,2.$ Moreover, Fig. 2 exhibits the time trajectories for the components of ${z}_{k}(t),\,k=1,\,2,$ with two initial conditions $\vartheta _1$ and $\vartheta _2$ at $\gamma =\mu =0.95.$

**Table 1:** The equilibrium points for system (3.1) at various values of $\gamma$ and $\mu .$.

$\gamma$	$\mu$	Equilibrium points as real and imaginary parts for ${z}_{1}$ and ${z}_{2},$ respectively.
0.95	0.95	(0.0120, − 0.0172) and (− 0.0059, 0.0117)
0.9	0.85	(0.0113, − 0.0165) and (− 0.0056, 0.0112)
0.85	0.65	(0.0077, − 0.0124) and (− 0.0043, 0.0088)
0.8	0.55	(0.0077, − 0.0122) and (− 0.0042, 0.0085)

### Example 3.2

Let the fractional-order CVNNs is described as follows:

3.2 $$\begin{aligned} ^{C }\mathscr {D}_{0}^{\gamma ,\mu }{z}({\mathfrak {t}})=-\mathscr {C}{z}({\mathfrak {t}})+\mathcal {G}\varphi ({z}({\mathfrak {t}}))+\mathcal {H}\varphi ({z}({\mathfrak {t}}-{\tau }))+\Upsilon , \end{aligned}$$

where $${\tau }=1,{z}_{k}({\mathfrak {t}})= \Psi _{k}({\mathfrak {t}})+\mathfrak {i}\Theta _{k}({\mathfrak {t}}),\,\varphi ({z}_{k})=\frac{{z}_{k}}{1+|{z}_{k}|},k=1,\,2,3,$$ and

$$\begin{aligned} \mathscr {C} =\begin{bmatrix} 1 &{} 0 &{} 0\\ 0 &{} 2&{} 0\\ 0 &{} 0&{} 2\\ \end{bmatrix}, \quad \mathcal {G} = \begin{bmatrix} 2-5\mathfrak {i} &{} 2+\mathfrak {i} &{} 2+\mathfrak {i} \\ 3 &{} 1+\mathfrak {i}&{} 0\\ 1-\mathfrak {i} &{} 0&{} 1+\mathfrak {i}\\ \end{bmatrix}, \end{aligned}$$

$$\begin{aligned} \mathcal {H} = \begin{bmatrix} 1.6+0.5\mathfrak {i} &{} -1+0.5\mathfrak {i} &{} 2+0.1\mathfrak {i} \\ 1-0.5\mathfrak {i} &{} -0.5+1.5\mathfrak {i}&{} 1+0.5\mathfrak {i}\\ -3+0.5\mathfrak {i} &{} 3.5+\mathfrak {i}&{} 0.2+0.1\mathfrak {i}\\ \end{bmatrix}, \Upsilon = [0\,\,0\,\,0]^{T}. \end{aligned}$$

The initial condition is given by

$$\begin{aligned} \vartheta _1= \begin{bmatrix} 1+0.3\mathfrak {i}\\ 0.5+0.4\mathfrak {i} \\ 0.15+0.3\mathfrak {i} \end{bmatrix}. \end{aligned}$$

**Figure 3:**
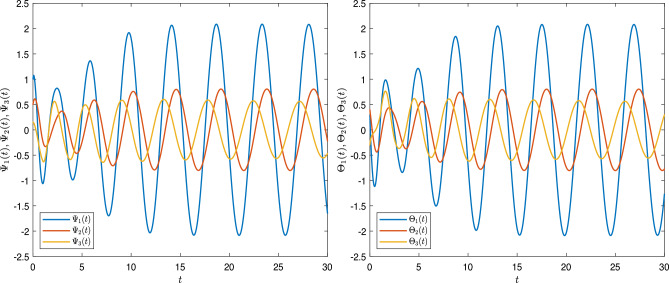
The trajectories of the real and imaginary parts of ${z}_{k},\,k=1,\,2,\,3$ for Example 3.2 at $\gamma = 0.95,\,\mu = 0.9.$.

**Figure 4:**
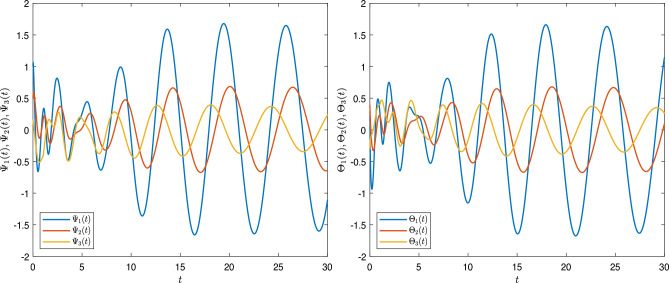
The trajectories of the real and imaginary parts of ${z}_{k},\,k=1,\,2,\,3$ for Example 3.2 at $\gamma = 0.8,\,\mu = 0.75.$.

**Figure 5:**
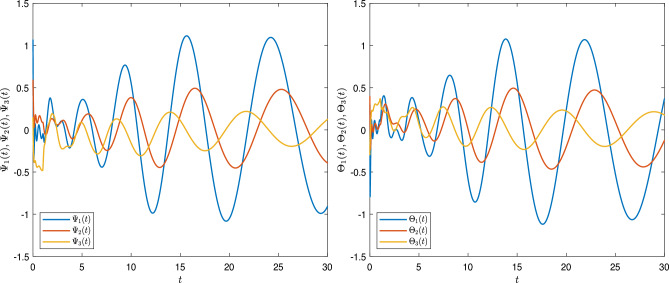
The trajectories of the real and imaginary parts of ${z}_{k},\,k=1,\,2,\,3$ for Example 3.2 at $\gamma = 0.65,\,\mu = 0.5.$.

**Figure 6:**
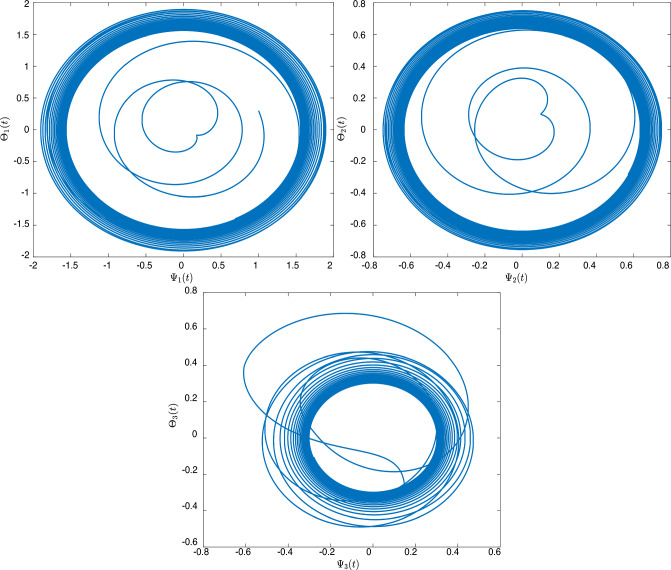
Chaotic attractor behaviors of ${z}_{k},\,k=1,\,2,\,3$ for Example 3.2 at $\gamma = 0.9,\,\mu = 0.8.$.

**Figure 7:**
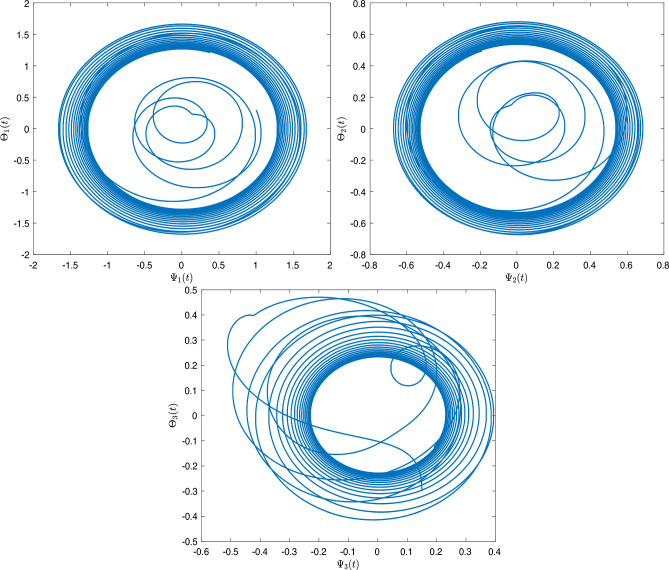
Chaotic attractor behaviors of ${z}_{k},\,k=1,\,2,\,3$ for Example 3.2 at $\gamma = 0.8,\,\mu = 0.75.$.

Figures 3, 4 and 5 represent the time trajectory for the components of ${z}_{k},k=1,2,3$ at $\gamma = 0.95,\,\mu = 0.9$, $\gamma = 0.8,\,\mu = 0.75$ and $\gamma = 0.65,\,\mu = 0.5,$ respectively. The numerical simulations of the real against imaginary parts are shown in Figs. 6 and 7. It is clear from these results that the system (3.2) displays chaotic behavior.

### Example 3.3

Let the fractional-order CVNNs is described as follows:

3.3 $$\begin{aligned} { ^{C }\mathscr {D}_{0}^{\gamma ,\mu }{z}({\mathfrak {t}})=-\mathscr {C}{z}({\mathfrak {t}})+\mathcal {G}\varphi ({z}({\mathfrak {t}}))+\mathcal {H}\hat{\varphi }({z}({\mathfrak {t}}-{\tau }))+\Upsilon , } \end{aligned}$$

where $${\tau }=0.5,{z}_{k}({\mathfrak {t}})= \Psi _{k}({\mathfrak {t}})+\mathfrak {i}\Theta _{k}({\mathfrak {t}}),$$

$$\begin{aligned} \varphi ({z}_{k})= & {} \frac{1-\exp (-\Psi _{k})}{1+\exp (-\Psi _{k})} + \mathfrak {i} \frac{1}{1+\exp (-\Theta _{k})},\,k=1,\,2,\\ \hat{\varphi }({z}_{k})= & {} \frac{1-\exp (-\Theta _{k})}{1+\exp (-\Theta _{k})} + \mathfrak {i} \frac{1}{1+\exp (-\Psi _{k})},\,k=1,\,2, \end{aligned}$$

and

$$\begin{aligned} {\mathscr {C} =\begin{bmatrix} 8 &{} 0 \\ 0 &{} 6\\ \end{bmatrix}, \quad \mathcal {G} = \begin{bmatrix} 2+3\mathfrak {i} &{} 3-\mathfrak {i} \\ 4-2 \mathfrak {i}&{} 1+2\mathfrak {i} \\ \end{bmatrix}, \mathcal {H} = \begin{bmatrix} -1+2\mathfrak {i} &{} 2+\mathfrak {i} \\ 3-4\mathfrak {i} &{} -3+2\mathfrak {i}\\ \end{bmatrix}, \Upsilon = [-3+\mathfrak {i}\,\,2+4\mathfrak {i}]^{T}.} \end{aligned}$$

**Figure 8:**
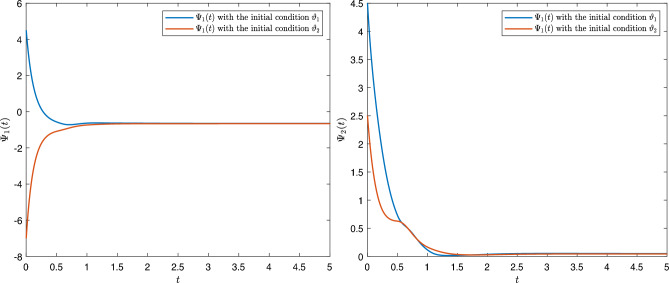
The trajectories of the real parts of ${z}_{k},\,k=1,\,2$ for Example 3.3 at $\gamma = 0.98,\,\mu = 1.$.

**Figure 9:**
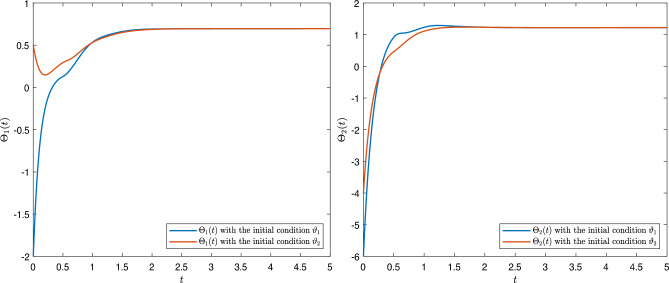
The trajectories of the imaginary parts of ${z}_{k},\,k=1,\,2$ for Example 3.3 at $\gamma = 0.98,\,\mu = 1.$.

Figures 8 and 9 show the time trajectory for the real and imaginary components of ${z}_{k},k=1,2$ at $\gamma = 0.98,\,\mu = 1,$ and the following two initial conditions

$$\begin{aligned} {\vartheta _1= \begin{bmatrix} 4.5-2\mathfrak {i}\\ 4.5-6\mathfrak {i} \\ \end{bmatrix}, \text {and} \quad \vartheta _2= \begin{bmatrix} -7+0.5\mathfrak {i}\\ 2.5-4\mathfrak {i} \\ \end{bmatrix}.} \end{aligned}$$

It is evident that the solutions converge to an equilibrium point that is consistent with the obtained theoretical results. Table 2, shows the equilibrium points at various values of $\gamma$ and $\mu$ for ${z}_{k}({\mathfrak {t}}),\,k=1,\,2.$ Moreover, we compared the values for the obtained equilibrium points for our results at $\gamma =0.98$ and $\mu =1$ with those obtained in^18^. It’s worth mentioning that the numerical method is based on a decomposition formula for the generalized Caputo derivative, for more details, see^22,23^.

**Table 2:** The equilibrium points for system (3.3) at various values of $\gamma$ and $\mu .$.

$\gamma$	$\mu$	Equilibrium points for our results	Equilibrium points in^18^
0.98	1	(− 0.6506, 0.0491) and (0.6958, 1.2216)	(− 0.6542, 0.0432) and (0.6979, 1.2275)
0.9	0.8	(− 0.6309, 0.0815) and (0.6843, 1.1897)	–
0.85	0.75	(− 0.6154, 0.1070) and (0.6752, 1.1645)	–

### Remark 3.1

- In recent past, many authors have demonstrated the various stability results of fractional order neural networks in Banach spaces making use of the contractive map. (check^18,19^, for more info)
  - As compared to the above results, we have used the complex-valued metric space to demonstrate the uniform stability of the problem.
- In order to investigate the stability of the equilibrium point and demonstrate its existence and uniqueness for generalized Caputo fractional-order, the authors of^20,21^ have taken into consideration real-valued neural networks.
  - As compared to the above results, we first utilized complex-valued neural networks in the setting of generalized Caputo fractional-order.

## Open Questions

Prove or disprove the following:

- Synchronization and uniform stability of neural networks having complex valued fractional order;
- Finite time stability of neural networks having Atangana-Baleanu fractional derivative with complex order^43^;
- Extend our results to complex orders.

## Conclusion

The uniform stability of CVNNs under the setting of generalized Caputo fractional-order with time delays in complex-valued metric spaces is investigated. The results of CVNNs are also developed in the context of fractional-order with time delay in complex-valued metric spaces, which produces fixed-point and unique equilibrium-point results. Additionally, numerical examples are given to support and illustrate the theoretical results. Our findings are significant because they open up novel possibilities for studying neural systems and chaotic theory. The findings of this work provide additional research directions for:

- Finite-time projective synchronization of generalized Caputo fractional-order complex-valued neural networks;
- Feedback synchronization of the generalized Caputo fractional-order and application to computational topology;
- Physics-informed neural networks for fractional-order model of system identification.

## Data Availability

The data sets used and/or analyzed during the current study available from the corresponding author on reasonable request.
